# Reconstructing the single-cell spatiotemporal dynamics of glioblastoma invasion

**DOI:** 10.1038/s41467-026-78908-9

**Published:** 2026-11-02

**Authors:** Hitesh Bhagavanbhai Mangukiya, Madeleine Skeppås, André Lasses Armatowski, Irem Uppman, Linnea Hallin, Filippa Kruse, Clara Nordquist, Soumi Kundu, Maria Berglund, Adam A. Malik, Cecilia Krona, Rebecka Jörnsten, Sven Nelander

**Affiliations:** 1 Department of Immunology, Genetics and Pathology, Uppsala University, Uppsala, Sweden; 2 Department of Mathematical Sciences, Chalmers University of Technology and University of Gothenburg, Gothenburg, Sweden; 3 Science for Life Laboratory (SciLifeLab), Uppsala University, Uppsala, Sweden; 4 Uppsala University Future Institutes (UUniFI), Uppsala University, Uppsala, Sweden

**Keywords:** CNS cancer, Cancer microenvironment, Cell invasion, Image processing, Time-lapse imaging

## Abstract

Glioblastoma invasion into healthy brain tissue remains a major barrier to effective treatment, but the dynamic single-cell behaviors underlying this process remain difficult to measure across patient models. Here, we introduce GlioTrace, an ex vivo imaging and computational framework for real-time tracking of glioblastoma invasion in patient-derived xenograft brain slices. Combining whole-specimen confocal microscopy with machine learning-based morphology classification and probabilistic modeling of state transitions, GlioTrace reconstructs how tumor cells switch between invasion phenotypes and how these dynamics are shaped by local tissue context and therapy. Across six patient-derived models, GlioTrace reveals that invasion plasticity is patient-specific, spatially organized, and modulated by vascular proximity and microglial interaction. Targeted therapies selectively alter phenotype composition and transition probabilities, whereas standard-of-care treatment enriches a highly motile, transition-connected branching state consistent with a treatment-tolerant invasive phenotype. Thus, GlioTrace makes glioblastoma invasion a measurable dynamic process for comparing patient-specific treatment responses in living brain tissue.

## Abstract (web summary)

Glioblastoma cells can spread through surrounding brain tissue by adopting different migratory states, but the dynamics of these transitions are difficult to measure. Here, the authors develop GlioTrace, a single-cell imaging and computational framework that tracks tumor invasion in patient-derived brain slice models and shows that invasion behaviors are patient-specific, influenced by local tissue interactions, and differentially altered by targeted therapies and standard-of-care treatments.

## Introduction

Glioblastoma (GBM) is one of the most aggressive and treatment-resistant cancers^1^, marked by rapid growth and widespread infiltration into surrounding brain tissue. Standard therapies, such as the Stupp protocol^2^, target the tumor mass but cannot eliminate invasive cells that have spread beyond resectable margins—a primary driver of recurrence and treatment failure. Extensive dissemination, especially into the brainstem is linked to poorer survival outcomes^3–7^. Conversely, greater surgical removal of invasive cells correlates with longer survival^8,9^. Understanding and targeting these invasive properties is therefore a critical priority.

GBM invasion is shaped by a complex interplay of cellular and microenvironmental factors. First, as GBM cells invade the brain at the tumor periphery, they engage in intricate interactions with normal brain structures^10^, including co-opting blood vessels as migratory scaffolds^11–13^, penetrating white matter tracts^14^, and even forming synaptic contacts with neurons^15–17^. Second, GBM cells exist in multiple transcriptional states that resemble normal brain cell types and their precursors^18–22^. These states are differentially distributed across the tumor, raising the possibility that distinct cellular states support distinct invasion behaviors. Third, GBM cells demonstrate extensive plasticity, dynamically altering their morphology and differentiation over time. According to the early go-or-grow model, GBM cells switch between migratory and proliferative states^23,24^, though more recent lineage-tracing studies suggest a more intricate hierarchy of cell state transitions, with stem-like cells at the apex^19,21,25^. Collectively, these findings highlight that GBM invasion is a dynamic and multifaceted process, yet many aspects remain poorly understood. This raises two key questions: whether plastic transitions and cell-cell interactions can be therapeutically modulated, and to what extent invasion programs vary across patients. Addressing these questions requires long-term, single-cell resolution observations across large populations of invading cells within intact brain tissue, a scale and duration that current experimental approaches cannot readily achieve.

In this work, we introduce GlioTrace, a scalable platform for real-time quantitative analysis of GBM invasion using patient-derived glioma cell culture xenograft (PDCX) brain slices. By combining whole-specimen confocal imaging with computational single-cell image analysis, including convolutional neural networks and covariate Hidden Markov Models, GlioTrace enables continuous monitoring of thousands of tumor cells within intact brain tissue, capturing invasion phenotypes, morphological transitions, and patient-specific dynamics. Compared with conventional intravital approaches, GlioTrace is designed to increase observational scale and duration while remaining compatible with standard high-content microscopy infrastructure. Applying GlioTrace to six patient-derived models, we identify distinct invasion modes, including perivascular translocation, diffuse locomotion, and a highly motile branching phenotype that frequently transitions between states. Using Hidden Markov Models, we infer a hierarchy of morphological states and show that pharmacological perturbation selectively modulates specific invasion phenotypes in spatially and temporally distinct ways. Together, these findings reveal that GBM invasion is a heterogeneous and plastic process with patient-specific vulnerabilities that can be quantified and targeted. GlioTrace runs on standard high-content microscopes and the accompanying analysis software is freely available. We expect the method to deepen our understanding of GBM invasion and expedite the discovery of tailored anti-invasion therapies for GBM.

## Results

### Whole-specimen monitoring of viable glioblastoma brain slices linked to automated analysis

**Fig. 1:**
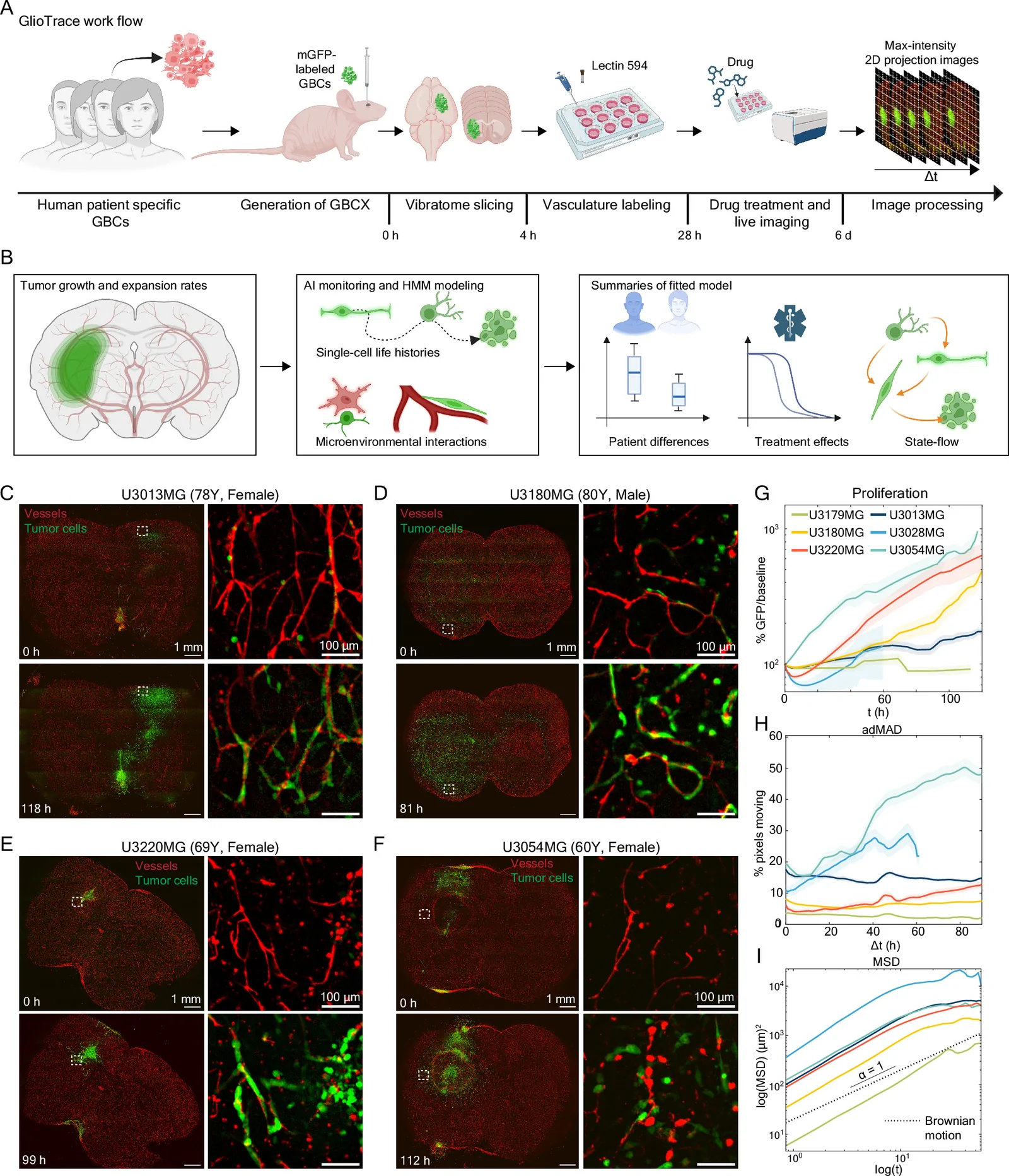
Overview and characterization of glioblastoma PDCX tumors in slice culture. **A** Experimental workflow for patient-derived glioblastoma xenograft (PDCX) brain slice culture. (Created in BioRender. Nelander, S. (2026) https://BioRender.com/dzgmfn6). **B** GlioTrace framework integrating imaging, cell tracking, and computational modeling. (Created in BioRender. Nelander, S. (2026) https://BioRender.com/dzgmfn6). Representative confocal images showing perivascular migration of bulky glioblastoma cells (**C**) (*n* = 39 slices from 16 mice), diffusely growing glioblastoma cells showing both perivascular and diffuse migration (**D**) (*n* = 18 slices from 6 mice), and additional examples of perivascular migration in bulky glioblastoma tumors (**E**, **F**) (*n* = 24 slices from 11 mice) at the first and last time points. Tumor cells are labeled with GFP (green) and vasculature with TL–DyLight 594 (red). **G** Quantification of tumor growth in different PDCX slices measured as the percent increase in GFP signal relative to baseline (*n* = 58 slices from 35 mice). Data are presented as mean values ± SEM (bold lines and shaded bands, respectively). **H** Quantification of cell movement measured as the percent spatial rearrangement of the GFP signal between consecutive frames (*n* = 56 slices from 34 mice). Data are presented as mean values ± SEM (bold lines and shaded bands, respectively). **I** Log–log plot of mean squared displacement (MSD), where α represents the slope of the curve. A slope of 1 indicates Brownian motion (dotted line) (*n* = 56 slices from 34 mice). The bold line represents the mean. Source data are provided as a Source data file.

GlioTrace combines long-term whole-specimen imaging of glioblastoma brain slices with automated computational analysis (Fig. 1). The first component is robust imaging of 300-micron-thick brain slices from established PDCX tumors grown in mouse hosts (Fig. 1A). Organotypic approaches that seed exogenous tumor cells onto pre-cut naive brain slices^26–31^ provide tractable in vitro models but do not recapitulate the native tumor architecture established during orthotopic growth. Intravital imaging through cranial windows^32,33^ enables single-cell resolution in living animals but is restricted to superficial cortical fields and cannot provide the full cross-sectional sampling needed for population-level phenotyping. GlioTrace addresses these limitations by imaging slices from mice bearing orthotopically established PDCX tumors, ensuring that tumor cells are situated within native brain architecture and preserving tumor-stroma spatial relationships.

Importantly, this approach enables direct scalable observation of tumor cells situated deep within the brain parenchyma, including regions inaccessible to conventional cranial window intravital imaging, while retaining the spatial organization of tumor cells across anatomical structures. Tumor cells are marked by GFP, and the tissue-penetrant conjugate Tomato Lectin – DyLight 594 (TL–DyLight 594), selective for endothelial cells and microglia/macrophages, is applied to provide microenvironmental context (Supplementary Fig. 1A, B and Supplementary Movie 1A). Slice integrity was confirmed by H&E and STEM121 immunohistochemistry staining comparison with corresponding in vivo PDCX tumors (Supplementary Figs. 1E and 2). Cell death and proliferation status, further validated by cleaved caspase-3 and Ki67 immunofluorescence staining and longitudinal single-cell transcriptomic profiling as described below, demonstrated substantial stability of PDCX slice cultures (Supplementary Fig. 3A–J). Over 4–6 days, GlioTrace captures the whole specimen at 10X resolution across approximately 100 time points at intervals of less than 2 h, generating 9000 × 9000 pixel videos sufficient to resolve slender cellular protrusions (Supplementary Dataset 1, sheet 1). This imaging regime yields whole-slice longitudinal datasets at a scale sufficient for tracking and classifying thousands of individual tumor cells per experiment, which is essential for robust estimation of phenotype composition and state-transition structure. The second component of GlioTrace is a computational pipeline that stabilizes and denoises imaging data, detects and tracks individual cells, classifies morphological states, models state transitions, and estimates patient-specific differences and treatment effects (Fig. 1B). Specifically, the pipeline couples cell tracking to morphological classification and microenvironmental featurization, stratifying each cell at each time point into one of five morphological classes corresponding to four distinct migration behaviors and one low-motility phenotype. Transitions between these states are modeled using a covariate Hidden Markov Model in which the CNN posterior probabilities serve as observations and local microenvironmental features are incorporated as covariates on the transition probabilities. The framework additionally provides image stabilization, whole-slice growth statistics, and regional and single-cell migration metrics. The approach is scalable to six whole-brain PDCX slices in parallel over 5 days, covering thousands of cells.

### GlioTrace detects divergent glioblastoma invasion dynamics across patient-specific models

Using GlioTrace, we analyzed PDCX brain slices from six patient cases drawn from the HGCC Phenobank collection, spanning proneural, mesenchymal, and classical transcriptional subtypes. Four models—U3013MG (78 y female, proneural), U3028MG (72 y female, proneural), U3054MG (60 y female, mesenchymal), and U3220MG (69 y female, mesenchymal) — exhibited prominent bulky and perivascular invasion patterns (Fig. 1C, E, F and Supplementary Fig. 1C). The remaining two—U3180MG (80 y male, classical) and U3179MG (62 y male, classical)—demonstrated diffuse invasion with extensive white matter involvement (Fig. 1D and Supplementary Fig. 1D). Notably, U3220MG, derived from a gliosarcoma, displayed strong subventricular and meningeal growth, while U3054MG, derived from a recurrent tumor, showed pronounced perivascular invasion (Fig. 1E, F). Key genomic and clinical annotations for all six models, including major driver alterations and MGMT promoter methylation status, are provided in Supplementary Dataset 1, sheet 2.

Time-lapse sequences confirmed these distinctions dynamically. Cells in bulky and perivascular models formed advancing edges with both individual and collective stream-like migration along vascular routes (Supplementary Movie 1B–E), while cells in diffusely growing models showed sporadic vessel interactions and predominantly individual migration (Supplementary Movie 1F–G). U3179MG cells migrated as discrete entities regardless of vessel contact (Supplementary Movie 1G).

To quantify these differences systematically, we measured three complementary metrics. Tumor growth, assessed from GFP signal intensity, was confirmed in five of six models, with U3220MG and U3054MG showing the most rapid expansion (Fig. 1G). Population-level spatial rearrangement, quantified using the adjusted mean absolute deviation (adMAD; “Methods”), was highest in U3054MG and U3028MG (Fig. 1H). Single-cell tracking further revealed that bulky tumor models exhibited anomalous, directed diffusion with larger MSD intercepts, while diffusely growing models showed slower but still directed movement (Fig. 1I; “Methods”). Together, these metrics establish a consistent patient-specific ranking of invasion dynamics that is reproducible across slices and mice.

### Longitudinal single-cell profiling confirms slice microenvironment stability and reveals diverse tumor cell states

**Fig. 2:**
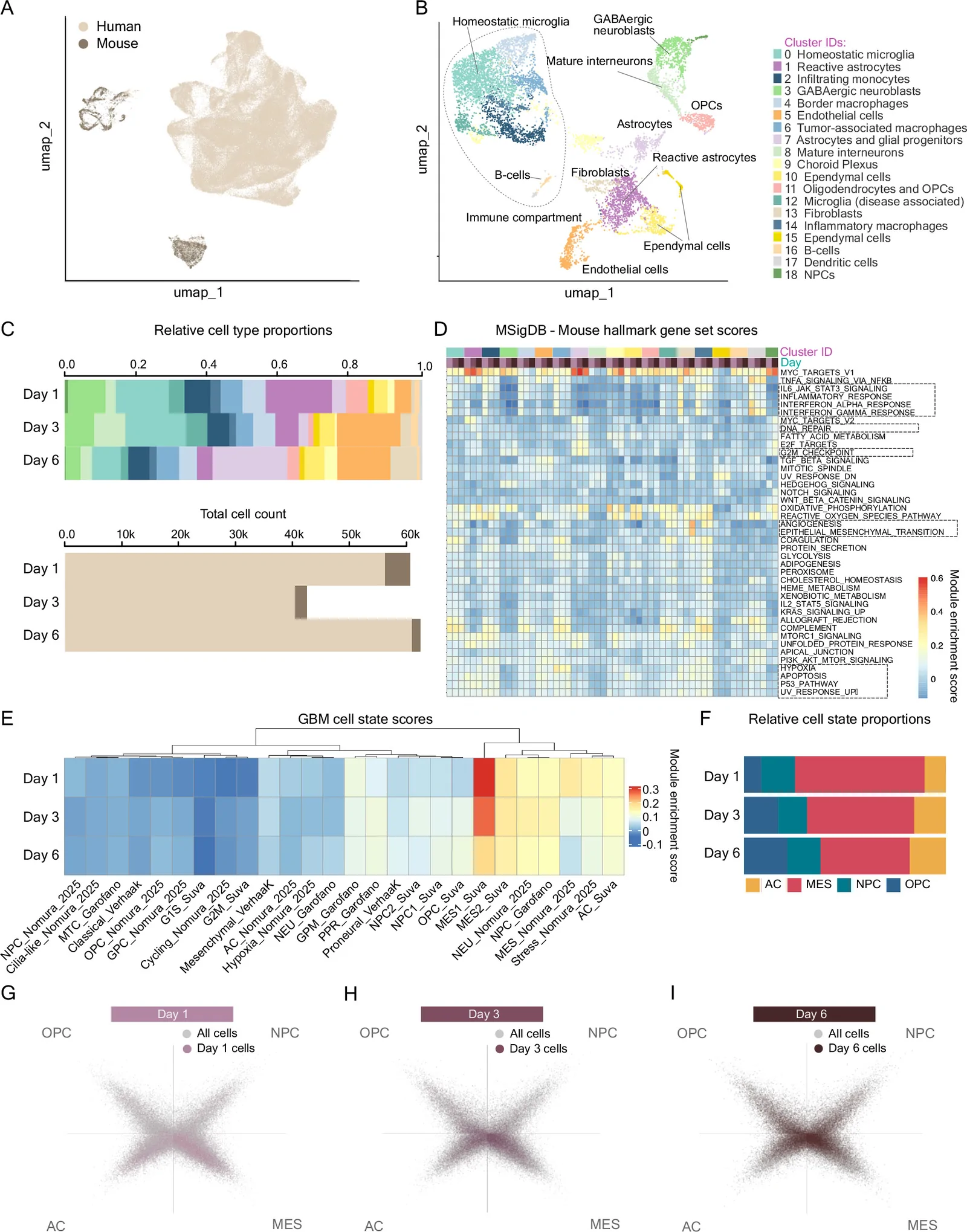
Single-cell transcriptomic characterization of PDCX brain slices over time. **A** UMAP projection of single-cell RNA sequencing data showing separation of human tumor cells and mouse brain cells (*n* = 166,216 cells isolated from 24 slices of 4 mice). **B** UMAP of mouse-derived cells annotated by cell type based on transcriptional profiles. **C** Relative mouse cell type proportions and total cell counts across time points (Day 1, Day 3, Day 6). **D** Heatmap of MSigDB hallmark gene set scores across mouse cell clusters and time points. **E** GBM cell state scores across time points, based on published glioblastoma transcriptional programs. **F** Relative proportions of glioblastoma transcriptional states (AC – astrocyte-like, MES – mesenchymal-like, NPC – neural progenitor cell-like, OPC – oligodendrocyte precursor cell-like) across time points. Projection of tumor cells onto the GBM transcriptional state landscape highlighting cells from Day 1 (**G**), Day 3 (**H**), and Day 6 (**I**). The data come from 24 slices (4 slices pooled for each time point for control and treatment groups) from 4 individual mouse brains. Source data are provided as a Source data file.

To characterize the cellular composition of the slice microenvironment and the transcriptional states of tumor cells during the imaging window, we performed single-cell RNA sequencing on U3013MG PDCX slices at days 1, 3, and 6 post-sectioning (Fig. 2A). Unsupervised clustering of non-malignant mouse cells resolved 19 clusters corresponding to the major brain cell populations, including neurons, oligodendrocyte-lineage cells, astrocytes, microglia, endothelial cells, and perivascular stromal cells (Fig. 2B, C). Hallmark gene set scoring across clusters showed no substantial transcriptional differences between time points, and cell-death programs were not upregulated at day 3 or day 6 (Fig. 2D). Immunostaining for GFAP, β-tubulin, CD11b, CD13, and CD31 together with cleaved caspase 3 confirmed that astrocytes, neurons, myeloid cells, pericytes, and endothelial cells retained normal morphology with minimal apoptotic activity throughout the experiment (Supplementary Fig. 4A–E). Together, these transcriptomic and immunostaining data validate that the slice microenvironment remains compositionally intact and transcriptionally stable across the imaging window used for GlioTrace, supporting its use for longitudinal observation of tumor–microenvironment interactions. Analysis of the human tumor cells showed that multiple coexisting transcriptional states (OPC-like, NPC-like, AC-like, and MES-like) were present throughout the experiment (Fig. 2E–G). Tumor cells were predominantly MES-like at day 1 and shifted toward NPC-like and OPC-like states by day 6, likely reflecting the progressive invasion of tumor cells into the surrounding slice tissue (Fig. 2F–I). Altogether, the presence of multiple coexisting transcriptional states within a single model mirrors the heterogeneity observed in primary glioblastoma and provides a transcriptional context for the diverse invasion phenotypes that we next characterize at single-cell resolution.

### GlioTrace reveals patterns of phenotypic state switching and migration route change

**Fig. 3:**
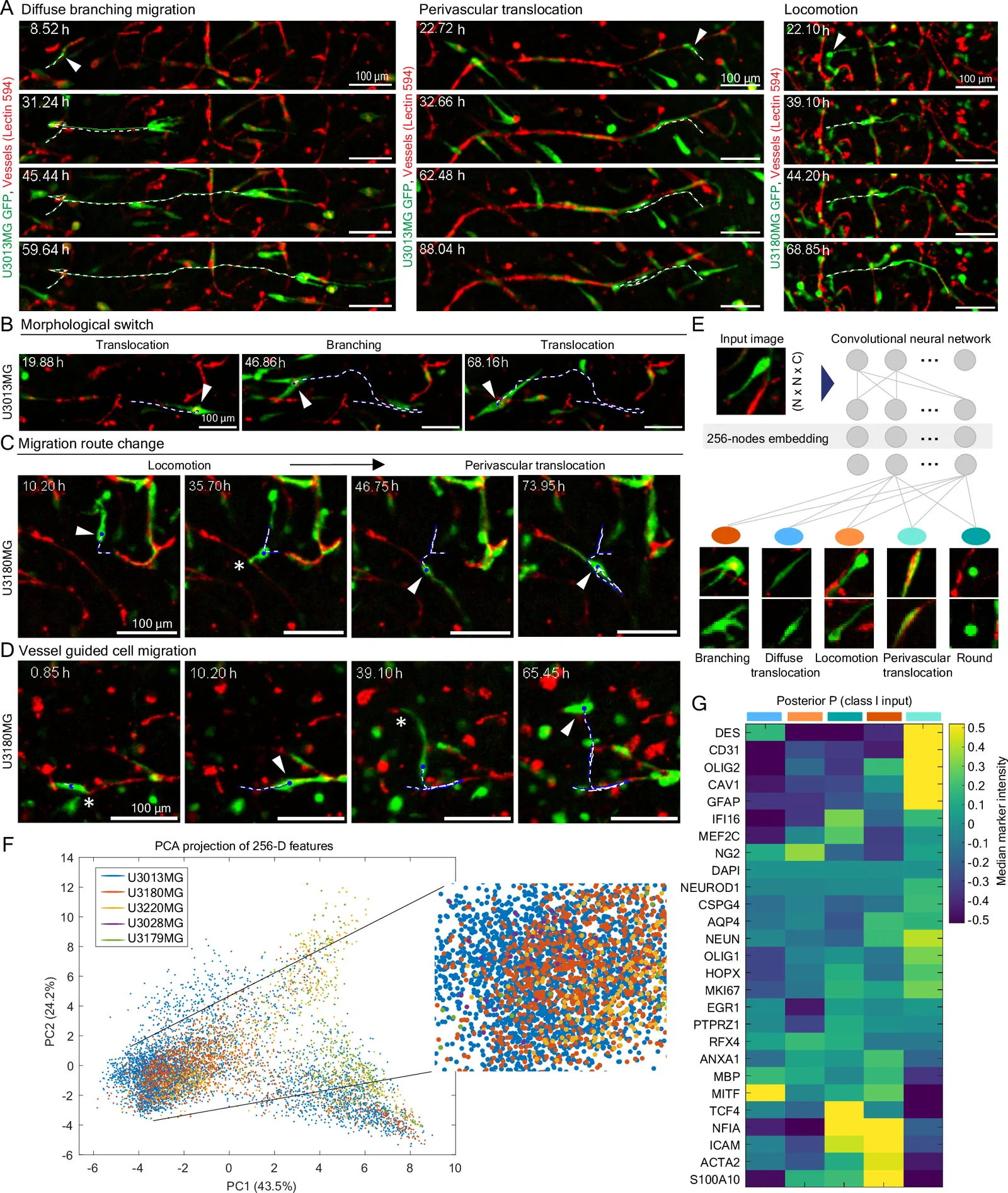
Detection and characterization of migratory and invasive mechanisms of glioblastoma cells in PDCX slice culture. **A** Time-lapse confocal images of tumor cells displaying diffuse branching migration, perivascular translocation, and locomotion. Tumor cells are labeled with GFP (green) and vasculature with TL–DyLight 594 (red). **B** Example of a glioblastoma cell undergoing morphological switching between translocation and branching states. Scale bars: 100 μm. **C** Example of a tumor cell changing migration routes from locomotion to perivascular translocation. Scale bars: 100 μm. **D** Example of vessel-guided migration along the vasculature. Scale bars: 100 μm. Arrowheads in (**A**–**D**) indicate tracked tumor cells; asterisks in (**C**, **D**) indicate vessel contact. Dashed lines indicate cell trajectories. The images in **A**–**D** represent observations of 57 slices from 22 mice. **E** Schematic of tumor cell morphology classification from image patches by a CNN. The network assigns cells to one of five morphological classes: branching, diffuse translocation, locomotion, perivascular translocation, and round. The 256-layer embedding before the final layer is highlighted. (An additional outlier class used during training is not shown). **F** PCA projection of the last 256-dimensional ReLU layer before softmax, illustrating learned features of morphological states across PDCX models (*n* = 8 slices from 8 mice). **G** Heatmap showing relative protein marker enrichment across morphological classes inferred by applying the classifier to multiplex immunohistochemical data (*n* = 16 from 16 mice). Source data are provided as a Source data file.

We went on to investigate different classes of migratory cells detectable by GlioTrace at the tumor periphery. Through the analysis of multiple regions of interest (ROIs), we identified three distinct migratory phenotypes. First, branching cells exhibited long cytoplasmic protrusions extending at various angles from the cell body, with PDCX models U3013MG and U3028MG frequently demonstrating this phenotype (Fig. 3A left, Supplementary Movie 2A). Second, translocating cells appeared spindle-shaped with central nuclei and forward and backward protrusions, often engaging with vasculature during migration (Fig. 3A middle, Supplementary Movie 2B). Third, locomotive cells displayed unidirectional migration with elongated leading-edge protrusions and nuclei positioned at the rear. This phenotype was prominent in U3180MG, where cells often migrated along vascular routes (Fig. 3A right, Supplementary Movie 2C). These three phenotypes (branching, translocating, and locomotive) correspond closely to invasion modes described in prior intravital imaging studies, including neurite-like branching, perivascular translocation, and diffuse locomotion^31–36^. In addition, we observed round cells and atypical morphologies that did not conform to these categories. Together, these findings show that GlioTrace captures established invasion behaviors in a scalable slice culture system and extends prior work by enabling their systematic quantification and transition modeling across large cell populations.

Beyond these core phenotypes, we observed several intriguing dynamics. Tumor cells demonstrated phenotypic switching, transitioning between branching and translocating states, and reverting over time (Fig. 3B, Supplementary Movie 2D). Additionally, cells altered their migration routes, shifting between diffuse locomotion and perivascular migration (Fig. 3C, Supplementary Movie 2E). Notably, in U3180MG, diffusely invading cells alternated between vessel-associated and non-vessel-associated migration, indicating a highly adaptive form of invasion (Fig. 3D and Supplementary Movie 2F). These observations indicate that glioblastoma cells can switch between modes of migration.

### Automated classification reveals distinct glioblastoma invasion morphologies

To capture these phenotypes systematically, we established a convolutional neural network classifier designed to distinguish five principal morphological classes: branching (B), diffuse translocation (DT), locomotion (L), perivascular translocation (PT), and round (R) (Fig. 3E). An additional crowded/outlier class was included during network training to capture ambiguous or overlapping cells but was excluded from downstream analyses. The network achieved high overall accuracy (Supplementary Fig. 5A, E) and robustly separated these classes (87% accuracy) by assigning posterior probabilities based on a 256-dimensional latent feature representation with learned features from classes in all patients (Fig. 3F; Supplementary Fig. 5B). To ensure that classification was robust across patients and classes, we performed leave-one-patient-out validation, which demonstrated consistent per-class recall across models (Supplementary Fig. 5C, D). In the analyses below, these five morphological classes serve as the principal units of observation.

To obtain an initial molecular characterization of these morphologies, we revisited endpoint multiplex immunohistochemical data from Doroszko et al.^37^, comprising measurements for 26 proteins. Applying our neural network to score individual tumor cells in these specimens, we estimated protein expression patterns across the five morphological classes (Fig. 3G and Supplementary Fig. 5F). Several markers showed preferential association with specific morphological classes. For example, branching cells showed higher relative levels of markers such as DES, MBP, and MITF, diffuse translocating cells were enriched for NG2 and OLIG2, and locomoting cells were associated with markers including IFI16, MEF2C, TCF4, and NFIA. Perivascularly translocating cells showed relatively higher expression of ICAM, ACTA2, and ANXA1, while round cells were associated with markers including AQP4, NEUN, OLIG1, and HOPX (Fig. 3G). Although these associations are exploratory, they suggest that the image-defined morphological classes correspond to biologically distinct invasion-associated cell states.

### Hidden Markov modeling reveals dynamic transitions between morphological states

**Fig. 4:**
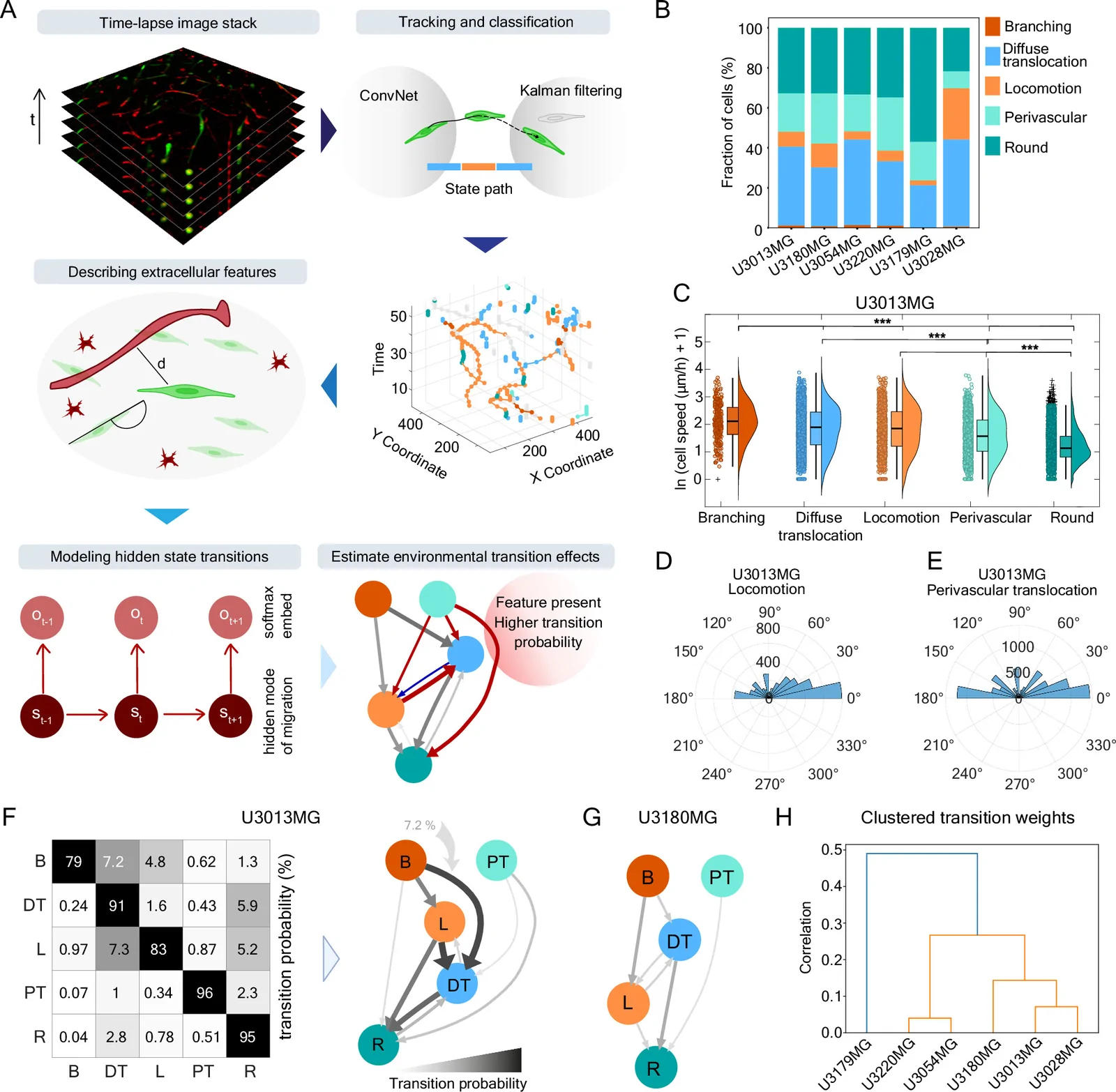
Hidden Markov modeling reveals dynamic transitions between morphological states. **A** Schematic overview of the GlioTrace computational framework. In time-lapse image stacks, cells are tracked with a Kalman filter and classified according to one of five morphologies, generating state paths in time and space for multiple cells simultaneously. Using these state paths, a Hidden Markov Model with a generalized linear model (covariate HMM) is used to infer hidden migration states and model state transitions, while incorporating the effects of extracellular features associated with each cell. **B** Distribution of morphological states across PDCX models (*n* = 123 slices from 56 mice). **C** Violin plot of speed distributions across morphological classes in the U3013MG model (*n* = 21 slices from 15 mice; 18,716 cells). Half violins show the kernel density estimate of the data distribution. Boxes indicate the interquartile range (IQR) (25th–75th percentiles), with a black center line indicating the median and whiskers extending to the most extreme data point within 1.5 × IQR. The underlying data are visualized as a jittered scatter. Differences among groups were assessed using one-way ANOVA. Post-hoc pairwise comparisons were performed using Tukey’s HSD. Results are reported as F(df1, df2), p, eta squared and 95% CI. F(4,10046) = 225.9529, *p* < 0.001, *η*^2^ = 0.162, 95% CI [0.149, 0.174]. **D**, **E** Turning-angle distributions of locomoting and perivascularly translocating cells in U3013MG, showing directional persistence of migration (*n* = 21 slices from 15 mice). **F** Left: Transition probability matrix inferred from the HMM for the U3013MG model. Values represent the probability of transitioning between morphological states. Right: State transition network for U3013MG. Gray arrow edge thickness reflects transition probability between modes of migration (*n* = 49 slices from 23 mice). (B – Branching, DT – Diffuse Translocation, L – Locomotion, PT – Perivasculalar Translocation, R – Round). **G** State transition network for U3180MG. Edge thickness reflects transition probability (*n* = 25 slices from 11 mice). **H** Hierarchical clustering of PDCX models based on inferred transition matrices (123 slices from 56 mice). Diagonal weights were excluded in clustering, which was done with complete linkage with correlation as the distance metric. Source data are provided as a Source data file. Significant Tukey-adjusted pairwise comparisons are indicated by asterisks. * = *p* ≤ 0.05, ** = *p* ≤ 0.01, *** = *p* ≤ 0.001.

Having established the morphological state classes in Fig. 3, we next sought to quantify their dynamics and interactions using a computational framework implemented in GlioTrace. Throughout the study, these classes are interpreted as morphological states representing distinct migration behaviors, which serve as the observable units in the subsequent Hidden Markov modeling. The analysis pipeline integrates convolutional neural network (CNN)–based morphology classification with single-cell tracking and probabilistic modeling of state transitions (Fig. 4A). Individual tumor cells were detected and tracked using Kalman filtering, generating trajectories in space and time from which morphological state assignments and movement statistics were derived.

Across all models, the relative abundance of the five morphological classes varied substantially (Fig. 4B). For example, diffusely invading cultures showed a higher proportion of locomotive and diffuse-translocation phenotypes, whereas bulk-forming tumors contained larger fractions of branching and perivascularly translocating cells. Quantitative analysis of single-cell tracks further revealed systematic behavioral differences between classes. Branching cells exhibited the highest migration speeds, whereas locomotive and perivascularly translocating cells showed more directional movement patterns (Fig. 4C–E and Supplementary Fig. 6A).

To infer the dynamic relationships between morphological states, we modeled state transitions using a Hidden Markov Model (HMM). In this framework, the CNN-derived class probabilities serve as observational evidence, while the hidden states represent the latent morphological states governing migration behavior (Fig. 4A). By analyzing thousands of cell trajectories, the HMM estimates the probabilities of transitions between morphological states over time. The resulting transition matrix revealed that certain states function as hubs within the state transition network (Fig. 4F). In particular, branching and diffuse-translocation phenotypes frequently transitioned into locomotive states, whereas round cells acted as terminal or sink states that rarely reverted to more migratory phenotypes.

Visualizing these transition probabilities as networks highlighted patient-specific differences in invasion dynamics (Fig. 4F, G; Supplementary Fig. 6B). While the overall structure of the transition graph was conserved across models, the relative strengths of transitions varied substantially between tumors. Spatial analysis further indicated that phenotypic states were not randomly distributed within the tissue. Nearest-neighbor analysis revealed moderate homotypic clustering of several states, particularly round and perivascular phenotypes, suggesting that invasion states form spatially structured neighborhoods within the tumor microenvironment (Supplementary Fig. 7). Clustering models based on their inferred transition matrices separated diffusely invading tumors from bulky, perivascularly growing tumors (Fig. 4H), consistent with the phenotypic patterns observed in the imaging data.

To evaluate whether the HMM framework provides advantages over simpler alternatives, we compared it with models based on raw CNN class counts and smoothed counts without explicit state modeling. We assessed robustness by bootstrapping the transition estimates across mouse-level datasets and measuring drift in inferred model weights (Supplementary Fig. 8A–C). While raw-count models showed substantial variability across bootstrap replicates, the HMM framework produced more stable transition weights, indicating improved robustness to sampling noise. These results indicate that the HMM adds value beyond temporal smoothing alone, by stabilizing transition inference under sampling variation while preserving biologically interpretable state trajectories. Inspection of state assignments further revealed that the HMM performs biologically plausible corrections of CNN predictions. In particular, a subset of cells initially classified as locomotive by the CNN were reassigned to diffuse-translocation states by the HMM (Supplementary Fig. 8D). Visual inspection of representative examples suggests that these corrections occur when weak vascular signal is present but under-interpreted by the CNN alone (Supplementary Fig. 8E). By incorporating temporal continuity and microenvironmental covariates, the HMM therefore improves consistency of state assignments while preserving the underlying biological interpretation of migration behaviors.

Together, these analyses demonstrate that GlioTrace not only classifies invasion phenotypes but also reconstructs the dynamic state transitions that govern glioblastoma invasion. This computational framework enables quantitative comparison of invasion behaviors across patient-derived models and provides a basis for analyzing how microenvironmental factors or therapeutic perturbations reshape invasion dynamics.

### Patient-specific vascular disorganization and microglial interactions at the tumor border

**Fig. 5:**
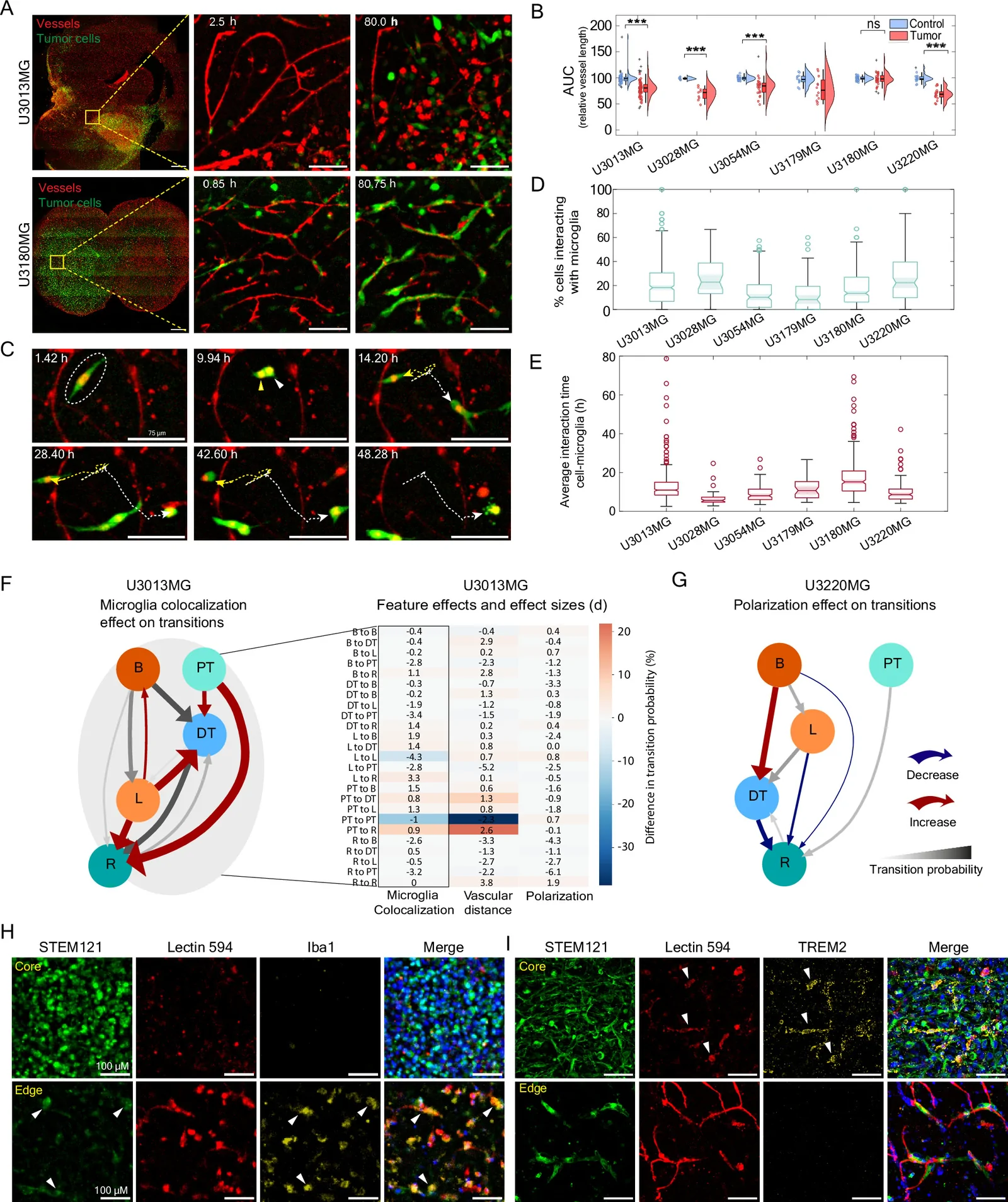
Vascular remodeling and immune interactions during glioblastoma invasion. **A** Whole-slice confocal images with zoomed time-lapse sequences illustrating vascular dynamics at the tumor border in U3013MG and U3180MG models. Tumor cells are labeled with GFP (green) and vasculature with TL-DyLight 594 (red). Dashed boxes indicate magnified regions. Scale bars: 1 mm (overview), 100 μm (zoomed panels). Representative of 10 slices from 5 mice. **B** Quantification of vascular disorganization at the tumor border. Violin plots show the area under the curve (AUC) of relative vessel length at the tumor border compared with tumor-free control regions across PDCX models (*n* = 56 slices from 34 mice). Statistical comparisons between groups were performed using Welch’s two-sample t-test (two-tailed). Results are reported as t(df), *p*, Hedges’ *g*, and 95% CI. Effect sizes were calculated as Hedges’ g using the pooled standard deviation and a small-sample correction, with 95% confidence intervals estimated from the standard error of Hedges’ g. U3013MG: t(86.14) = 3.370, *p* = 0.001126, Hedges’ *g* = 0.537, 95% CI [0.219, 0.855]. U3028MG: t(8.44) = 7.246, *p* = 6.726e-05, Hedges’ *g* = 3.253, 95% CI [1.873, 4.633]. U3054MG: t(25.05) = 4.228, *p* = 0.0002748, Hedges’ *g* = 1.200, 95% CI [0.594, 1.806]. U3180MG: t(38.00) = 0.645, *p* = 0.5228, Hedges’ *g* = 0.157, 95% CI [−0.321, 0.635]. U3220MG: t(28.44) = 9.359, *p* = 3.503e-10, Hedges’ *g* = 3.134, 95% CI [2.142, 4.127]. **C** Time-lapse sequence showing an example interaction between a tumor cell and a microglial/macrophage cell at the tumor periphery. Arrowheads indicate interacting cells and dashed lines indicate tracked trajectories. Scale bar: 75 μm. Images representative of *n* = 10 slices from *n* = 3 mice. **D** Percentage of tumor cells interacting with microglia/macrophages across PDCX models (*n* = 56 slices from 34 mice). Differences among groups were assessed using one-way ANOVA. Post-hoc pairwise comparisons were performed using Tukey’s HSD. Results are reported as F(df1, df2), *p*, eta squared and 95% CI. F(5,1157) = 13.778, *p* = 4.30 × 10⁻¹³, *η*²=0.056, 95% CI [0.030, 0.080]. **E** Average duration of tumor cell–microglia/macrophage interactions across PDCX models (*n* = 56 slices from 34 mice). Differences among groups were assessed using one-way ANOVA. Post-hoc pairwise comparisons were performed using Tukey’s HSD. Results are reported as F(df1, df2), *p*, eta squared and 95% CI. F(5,966) = 22.155, *p* = 4.58 × 10⁻²¹, *η*² = 0.103, 95% CI [0.066, 0.136]. **F** Effect of microenvironmental features on morphological state transitions inferred using the HMM-GLM framework. Left: differential transition network illustrating the effect of microglia colocalization on state transitions in the U3013MG model. Directional arrow edge thickness reflects increased (red) transition probability between modes of migration. Right: heatmap summarizing estimated effects and effect sizes for microglia colocalization, vascular distance, and polarization on transitions (*n* = 49 slices from 23 mice). **G** Example transition network illustrating the effect of directional polarization on invasion state transitions in the U3220MG model (*n* = 14 slices from 7 mice). Directional arrow edge thickness reflects increased (red) or decreased (blue) transition probability between modes of migration. **H**, **I** Immunofluorescence staining of PDCX slices following time-lapse imaging. Representative images from tumor core and invasive edge regions showing tumor cells (STEM121, green), vasculature (TL–DyLight 594, red), and microglia markers. **H** Iba1-positive microglia (yellow) enriched at the tumor edge (*n* = 3 slices from 3 mice). **I** TREM2-positive microglia/macrophages (yellow) enriched in the tumor core. Arrowheads indicate representative cells. Scale bars: 100 μm (*n* = 2 slices from 2 mice). The features of the violin/boxplots show the following: half violins show the kernel density estimate of the data distribution. Boxes indicate the interquartile range (IQR) (25th–75th percentiles), with a black centre line indicating the median and whiskers extending to the most extreme data point within 1.5 × IQR. The underlying data is visualized as a jittered scatter. For boxplot-only graphs, notches and shaded regions represent an approximate confidence interval around the median. Boxes with non-overlapping notches have significantly different medians (5% significance level). Significance is indicated by asterisks. * = *p* ≤ 0.05, ** = *p* ≤ 0.01, *** = *p* ≤ 0.001. Source data are provided as a Source data file.

While the PDCX brain slice model does not recapitulate the full immune repertoire of the brain tumor microenvironment, it retains several key structural and innate immune components, including intact vasculature and resident microglia, as evident from both microscopy and single-cell RNA sequencing. We therefore next examined whether GlioTrace could capture functional interactions between invading glioblastoma cells and these microenvironmental elements. A subset of our PDCX models exhibited extensive disorganization of the vasculature at the tumor front. In the U3013MG and U3028MG tumor slices, TL-DyLight 594-positive vascular structures were disrupted and retracted toward the tumor cell mass. Of note, vascular structures were disrupted even before physical contact between tumor cells and endothelial cells (Fig. 5A, Supplementary Fig. 9A, E, G, Supplementary Movie 3A, B), suggesting mediation via a soluble factor. Similarly, U3220MG and U3054MG formed connections with vessels while disintegrating endothelial organization (Supplementary Fig. 9C, D, I, J, Supplementary Movie 3C, D). In contrast, while the diffusely growing U3180MG and U3179MG tumor slices also exhibited perivascular connections of tumor cells, including examples of tumor-cell interactions with CD13-positive pericytes and vascular structures (Supplementary Movie 7), this occurred without disruption of the vascular network and with the majority of tumor cells migrating independently regardless of vessel contact (Fig. 5A; Supplementary Fig. 9B, F, H, Supplementary Movie 3E, F). To quantify these observations, we measured the extent of vessel disruption occurring around tumors by analyzing the length of vessels across the time-lapse, using a neural network for semantic vessel segmentation. When comparing the length of vasculature at the tumor border to vasculature within control (tumor-free brain) regions, we saw significant effects on vascular disorganization in all bulk-forming tumors and in one of the diffusely growing ones (Fig. 5B).

Having established vascular disruption as a patient-specific phenotype, we next examined interactions between tumor cells and the innate immune compartment of the slice model. In addition to vascular structures, GlioTrace captures a population of relatively round and motile TL-DyLight 594-positive microglia/macrophage cells at the tumor core and periphery (Fig. 5C; Supplementary Fig. 9K). The TL-DyLight 594 signal correlates well with the microglia marker Iba1 protein staining at the tumor periphery, which, together with the fact that TL-DyLight 594 stains microglia^38^, identifies this population as activated microglia (Supplementary Fig. 10A, C, D). GlioTrace, therefore, provides a means of probing microglia dynamics at the glioblastoma border. Intriguingly, we found examples of microglia seemingly chasing tumor cells at the tumor periphery, ending with the death of the tumor cell (Fig. 5E; Supplementary Movie 3G). Using a neural network trained to detect tumor cells co-localizing with microglia, we calculated the level of microglial engagement, defined as the percentage of tumor cells at the tumor periphery that co-localized with microglia, as well as the duration of co-localization. Broadly, the PDCX models with an ability to disorganize blood vessels showed higher levels of microglial/macrophage engagement (Fig. 5D). However, the relatively slow-moving U3180MG had a longer duration of microglial/macrophage engagement compared to the faster-moving cell lines (Fig. 5E).

To further characterize the microglial populations detected in the slice model, we performed immunostaining for the microglial markers Iba1 and TREM2. These analyses revealed a spatial compartmentalization of microglial states. Iba1-positive cells were predominantly observed at the tumor border, where they frequently contacted invading tumor cells, whereas TREM2-positive microglia were enriched within the tumor core (Fig. 5H, I; Supplementary Fig. 10A, B). This spatial distribution is consistent with transcriptional data from our single-cell RNA sequencing analysis, which showed that while both Iba1 and TREM2 are expressed in homeostatic microglia, Iba1 expression is elevated in inflammatory macrophage-like populations, whereas TREM2 is associated with tumor-associated microglia/macrophages localized within the tumor core (Supplementary Fig. 10E).

These results highlight the utility of GlioTrace in observing the intricate spatial and temporal relationships between glioblastoma cells, vasculature, and immune populations, providing a robust platform to elucidate dynamic cell-cell interactions at the tumor’s edge region.

### Microenvironmental features modulate invasion state transitions

Because invasion occurs within a complex tissue context, we next asked whether local microenvironmental features systematically bias transitions between invasion phenotypes using a covariate-dependent extension of the HMM framework.

Specifically, we modeled transition probabilities using a multinomial logistic regression conditioned on (i) the current cell state and (ii) local microenvironmental features, including proximity to blood vessels, microglial colocalization with GBM cells, and a measure of directional polarization among neighboring tumor cells.

This covariate HMM formulation separates a baseline transition network from context-dependent modulation of transition rates (Fig. 5F, G). The baseline model revealed a hierarchical transition structure similar to that described above, with branching cells frequently transitioning toward locomotive and diffuse translocation states. Incorporating environmental covariates revealed that specific microenvironmental contexts altered transition probabilities. For example, the presence of nearby microglia increased transitions from perivascular translocation to round states, as well as transitions from locomotion to diffuse translocation or round states (Fig. 5G).

Comparing models across multiple PDCX lines further demonstrated that all three environmental covariates, microglial colocalization, vascular distance, and local polarization, modulated transition probabilities in a patient-specific manner (Supplementary Fig. 11A–D). These results suggest that invasion phenotypes are not only intrinsic cell states but are dynamically influenced by the local microenvironment. Importantly, the covariate-HMM framework provides a principled statistical basis for disentangling intrinsic invasion dynamics from microenvironmentally gated transitions, quantifying not just what states cells occupy, but what drives them to switch, a distinction not accessible through endpoint or correlation-based analyses alone. To complement the covariate analysis with an experimental perturbation, we depleted microglia in U3013MG slices using clodronate. This reduced the microglia-associated modulation of transition dynamics while leaving overall tumor cell migration speed largely unchanged, consistent with a role for microglia in shaping invasion-state transitions more strongly than bulk motility (Supplementary Fig. 10F–H; Supplementary Movie 5).

### Targeted drugs alter invasion phenotypes and transition dynamics

**Fig. 6:**
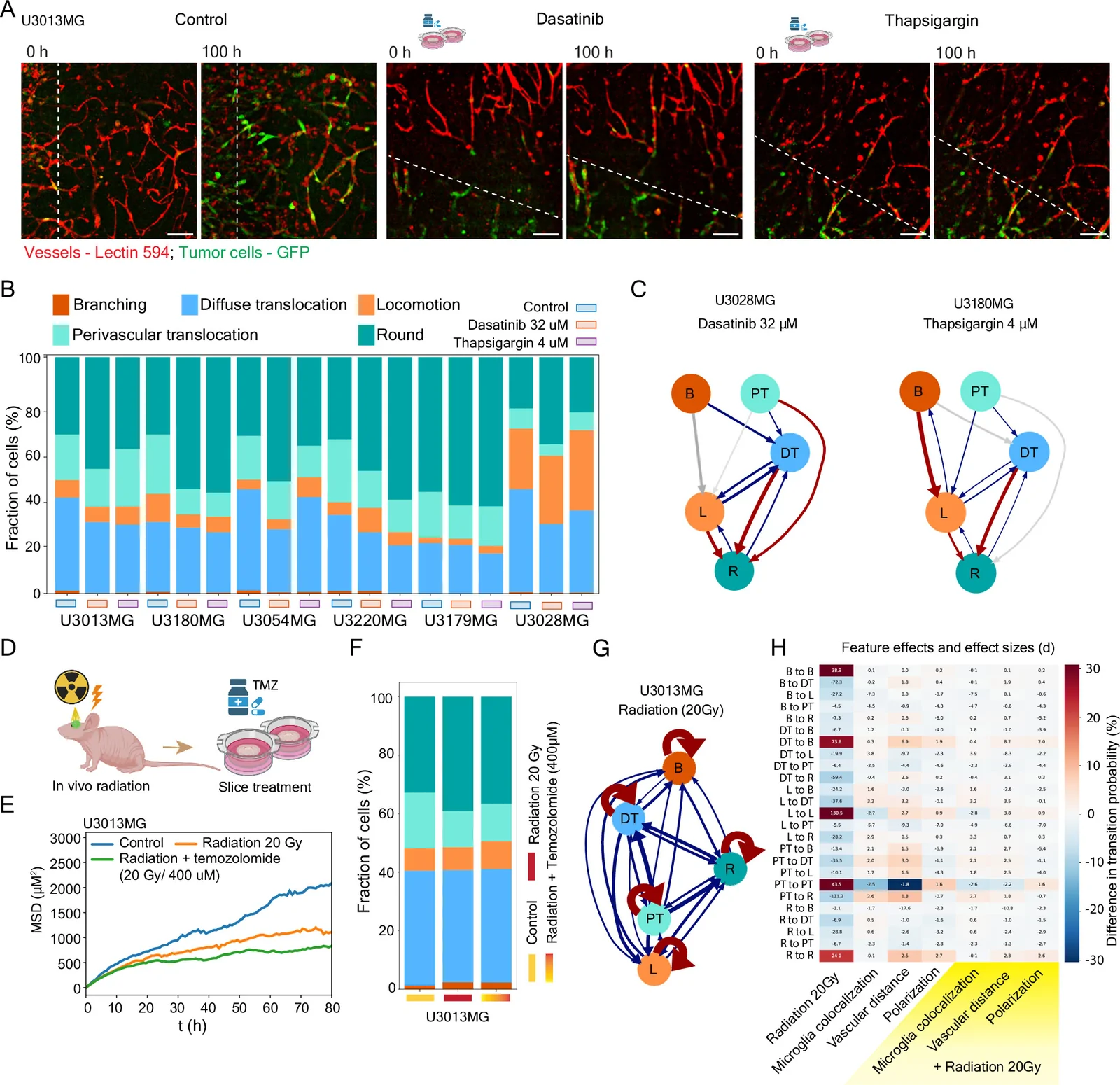
Drug treatment and standard-of-care therapies alter invasion phenotypes and transition dynamics in PDCX slice cultures. **A** Representative confocal images of U3013MG slices at the first (0 h) and last (100 h) time points under control conditions or after treatment with dasatinib (32 μM) or thapsigargin (4 μM). Tumor cells are labeled with GFP (green) and vasculature with TL–DyLight 594 (red). Dashed lines indicate the initial tumor border. Scale bar: 100 μm. Images are representative of 10 slices per treatment group from 6 mice). (Illustration created in BioRender. Nelander, S. (2026) https://BioRender.com/exdcu3r). **B** Distribution of morphological invasion phenotypes across PDCX models under control and drug-treated conditions. Stacked bar plots show the relative proportions of branching (B), diffuse translocation (DT), locomotion (L), perivascular translocation (PT), and round (R) states (*n* = 204 slices from 47 mice). **C** Differential transition networks inferred from the HMM-GLM framework showing the effect of dasatinib (left) and thapsigargin (right) on state transitions in representative models. Red edges indicate increased transition probabilities relative to matched control, whereas blue edges indicate decreased transitions. Edge thickness reflects the magnitude of the transition change (*n* = 27 slices from 9 mice). **D**, **E** Experimental design for standard-of-care treatment experiments. Tumor-bearing mice were irradiated in vivo (20 Gy total), after which brain slices were prepared and cultured ex vivo with or without temozolomide (TMZ) (*n* = 15 slices from 5 mice). (Illustration created in BioRender. Nelander, S. (2026) https://BioRender.com/exdcu3r). **F** Distribution of morphological invasion phenotypes in U3013MG slices under control, radiation-only, and radiation plus temozolomide conditions (*n* = 15 slices from 5 mice treated with radiation). **G** Differential transition networks showing treatment-induced rewiring of morphological state transitions following radiation alone or radiation combined with temozolomide. Red edges indicate increased transition rates relative to control, while blue edges indicate decreased transitions (*n* = 7 slices from 3 mice treated with radiation). **H** Heatmap of the effect of treatment and microenvironmental features, including interaction effects, on morphological state transitions inferred using the covariate HMM framework. Numbers in cells are Cohen’s *d* effect sizes (*n* = 7 slices from 3 mice treated with radiation). Source data are provided as a Source data file.

To test whether GlioTrace can detect pharmacologically induced changes in invasion dynamics, we treated slices with two mechanistically distinct compounds, implicated as the strongest hits in our previous in vitro screen for anti-migratory compounds across 41 patient-derived GBM cultures^39^. The first compound, dasatinib, is a clinically approved SRC-family kinase (SFK) inhibitor; SFK activity is concentrated at the invasive tumor front in GBM and regulates cytoskeletal remodeling, focal adhesion dynamics, and directed cell motility^40^. SFK inhibition has been linked to the invasive OPC-like transcriptional state in patient-derived GBM models^21^, and dasatinib has been evaluated in a randomized Phase II trial in recurrent GBM where the primary endpoint was not met in part due to restricted blood-brain barrier penetration but significant prolongation of response duration was observed in a subset of patients^41^^,^^42^; we therefore use it here as a mechanistically defined perturbation of SRC-family kinase signaling rather than as a clinical candidate. The second compound, thapsigargin, inhibits the SERCA pump, depleting ER calcium stores and triggering the unfolded protein response; GBM stem-like cells are selectively sensitive to perturbations of calcium homeostasis owing to higher expression of calcium-permeable ion channels and lower calcium-buffering capacity^43^^,^^44^, and thapsigargin-based compounds have been explored clinically as PSMA-activated prodrugs^45^. Together, these two compounds provide mechanistically complementary perturbations of tyrosine kinase signaling and calcium homeostasis, allowing evaluation of whether GlioTrace can resolve drug-specific effects on invasion dynamics in intact brain tissue. Using matched treated and untreated slices, we first examined qualitative effects of the drugs on tumor invasion. Time-lapse imaging revealed clear reductions in tumor expansion and cell dispersal over 100 h following treatment with either dasatinib or thapsigargin (Fig. 6A; Supplementary Fig. 12A–D; Supplementary Movie 4A–E). Spatial mapping further indicated that drug effects differed between tumor regions. In particular, dasatinib markedly reduced proliferation within the tumor core while leaving residual peripheral motility, whereas thapsigargin had weaker effects on core proliferation in some models (Supplementary Fig. 13).

Quantitative analysis revealed a dose-dependent reduction in cell speed across most models (Supplementary Fig. 14A–C). Linear mixed-effects modeling identified statistically significant treatment effects on migration speed in all models except U3179MG (Supplementary Fig. 14C). Drug treatment also altered the distribution of invasion phenotypes. In several models, drug exposure increased the proportion of round cells while reducing invasive morphologies such as branching and locomotion (Fig. 6B), consistent with a shift toward less invasive cellular states.

Branching cells, which were associated with rapid invasion (Fig. 4C), were particularly sensitive to both drug treatments. Dasatinib and thapsigargin both eliminated branching cells in U3013MG, whereas U3220MG retained branching cells following dasatinib treatment but lost them after exposure to thapsigargin (Fig. 6B).

To capture the underlying dynamics of these changes, we applied our Hidden Markov Model framework to estimate treatment-induced rewiring of morphological state transitions. Across models, transitions toward the round phenotype interpreted as consistent with cell death, were strongly increased (Fig. 6C; Supplementary Fig. 14E, F). However, drug-specific effects were also observed. For example, dasatinib-treated U3054MG cells showed a reduction in locomotion-to-diffuse translocation transitions, highlighting selective inhibition of specific invasive behaviors.

An important use of the covariate-HMM framework is that it allows us to estimate the relative contributions of direct drug effects, microenvironmental context, and their interaction to the observed changes in transition dynamics. For dasatinib, the best-fitted model attributed the dominant contribution to the treatment covariate itself, with minor contributions from microenvironmental interaction terms, suggesting that the primary locus of action is the tumor cell rather than the surrounding tissue (Supplementary Fig. 15). Consistent with this interpretation, single-cell RNA sequencing of dasatinib-treated slices revealed substantially more transcriptional perturbations in tumor cells than in the surrounding mouse brain compartment, with suppression of pathways consistent with SRC/ABL family kinase inhibition including cytoskeletal remodeling and adhesion-related programs (Supplementary Fig. 15). Together these analyses indicate that the invasion phenotype changes observed following dasatinib treatment primarily reflect direct drug action on tumor cells.

### Standard therapy alters invasion behavior through distinct mechanisms

Having established that GlioTrace captures the effects of targeted inhibitors on invasion dynamics, we next asked whether the platform could also detect responses to clinically relevant therapies. To this end, tumor-bearing mice were irradiated in vivo (20 Gy) prior to preparation of brain slices, which were subsequently treated ex vivo with temozolomide (Fig. 6D). Radiation reduced tumor cell motility and diffusivity as measured by mean squared displacement (Fig. 6E; Supplementary Fig. 14G), while speed remained unchanged (Supplementary Fig. 14H). The combination of radiation and temozolomide additionally altered invasion phenotype composition, including a consistent increase in the fraction of branching cells in some models (Fig. 6F; Supplementary Fig. 14I). Given that branching cells exhibit the highest migration speeds and occupy a highly connected hub position in the transition network, this enrichment is consistent with the persistence of a dynamically plastic invasion state under cytotoxic treatment pressure, raising the possibility that branching cells represent a treatment-tolerant phenotype that persists following depletion of more therapy-sensitive populations. HMM analysis revealed substantial rewiring of transition networks following treatment, with a notable pattern of reinforced self-transitions, that is, increased probability of cells remaining in their current state rather than switching, observed across both perivascular-invading U3013MG and diffusely invading U3180MG cells (Fig. 6G; Supplementary Fig. 14J, K). This effect was primarily attributable to radiation, as removal of temozolomide from the combination retained the pattern, suggesting that radiation reduces phenotypic plasticity by stabilizing cells within their current invasion state rather than driving transitions between states.

Together, these results demonstrate that GlioTrace captures multiple dimensions of treatment response, including migration speed, phenotypic composition, and the structure of phenotypic transition networks. These analyses show that pharmacological and standard therapies both reshape invasion dynamics, and suggest that GlioTrace could be used to classify therapeutic agents according to their effects on invasive behavior. More broadly, the framework provides a means to quantify rewiring of morphological state transitions and selective elimination of specific migratory phenotypes following chemical or genetic perturbations.

## Discussion

Glioblastoma invasion is not a uniform process but emerges from the dynamic interplay of heterogeneous tumor cell populations transitioning between distinct behavioral states. Here, we present GlioTrace, a scalable framework that resolves these dynamics at single-cell resolution across intact brain tissue, enabling systematic quantification of invasion phenotypes, their transitions, and their microenvironmental dependencies across patient-derived models. In practice, GlioTrace integrates whole-slice confocal imaging with convolutional neural network–based morphology classification and covariate Hidden Markov modeling of phenotypic transitions. Applied to six PDCX models from the HGCC collection, GlioTrace reveals that glioblastoma cells occupy a structured landscape of invasion states, including branching, translocating, and locomotive phenotypes, whose composition and transition dynamics are patient-specific, spatially organized, and modifiable by pharmacological perturbation. These findings establish that invasion plasticity, the capacity of tumor cells to transition between morphologically and behaviorally distinct states, is a reproducible and quantifiable property of glioblastoma that varies across patients and responds to therapeutic perturbation.

The invasion phenotypes analyzed by GlioTrace correspond closely to single-cell invasion modes previously characterized by intravital and live-cell imaging. Specifically, branching, locomoting, and translocating behaviors map directly to three modes defined by two-photon intravital microscopy in the cortex of patient-derived GBM xenografts^32^, and in the corpus callosum by three-photon microscopy^33^. Perivascular translocation in GlioTrace corresponds directly to vessel co-option and vascular-guided migration^31,34^; furthermore, the collective stream-like configurations seen in U3013MG are consistent with oncostreams, COL1A1-rich fascicles of mesenchymal-aligned cells^35^. Extending these contributions, GlioTrace enables their scalable and quantitative characterization. Whereas prior intravital approaches, including two-photon and three-photon microscopy through cranial windows^32,33^, which require specialized instrumentation and are limited by slow volumetric acquisition speeds that constrain multi-model throughput, are restricted to regions accessible via cranial window and analyze dozens to hundreds of cells, GlioTrace runs on standard high-content confocal microscopes, automates classification across thousands of cells spanning full anatomical cross-sections of patient-derived HGCC tumors, and reconstructs phenotype transition hierarchies using a probabilistic state-space framework not accessible to prior methods. This scale of analysis reveals properties not previously described, including the identification of branching cells as a transition hub with the highest connectivity in the phenotype network, and the quantification of microenvironmentally modulated transition probabilities. Consistent with a treatment-tolerant role for this highly connected state, standard-of-care treatment with radiation and temozolomide increased the fraction of branching cells in U3013MG (Fig. 6F), while the effect was absent in U3180MG, which has very low baseline branching cell frequency, raising the possibility that branching cells represent a preferentially persisting invasive subpopulation under treatment pressure.

A central question in glioblastoma invasion biology is whether switching between invasion modes reflects intrinsic cellular programs or microenvironmental cues. Our covariate-dependent HMM modeling provides a quantitative framework for addressing this question. Incorporating vascular proximity and microglial colocalization as predictors of transition rates revealed that transitions into perivascular translocating states are strongly associated with vascular proximity, consistent with vessels acting as permissive guidance structures for this invasion mode. In contrast, transitions involving branching and locomotive states showed weaker dependence on these environmental features. Critically, individual cells in models such as U3180MG repeatedly alternate between vessel-associated and non-vessel-associated migration within single trajectories. This behavior cannot be explained by passive environmental guidance alone and indicates that the capacity for mode switching is an intrinsic cellular property. Together, these observations support a two-component model of invasion plasticity: a cell-intrinsic baseline switching rate that is biased toward particular states depending on local context. This distinction carries therapeutic implications: if a substantial fraction of invasion plasticity is intrinsic, suppressing it will require targeting cell-autonomous programs rather than, or in addition to, disrupting microenvironmental guidance structures.

The patient-specific invasion patterns observed across HGCC models are consistent with underlying differences in transcriptional cell-state composition. Single-cell studies have established that glioblastoma tumors contain coexisting OPC-like, NPC-like, AC-like, and MES-like transcriptional programs, whose relative abundance shapes invasion behavior more than fixed subtype identity^19^. Oncogenic alterations appear to bias this distribution without strictly determining it. This spatial organization is consistent with recent single-cell analyses showing that glioblastomas comprise multiple coexisting transcriptional states whose relative abundance is influenced, but not fixed, by underlying genetic alterations^19,37,39^. Genetic events such as PDGFRA amplification, EGFR activation, or CDK4 amplification bias tumors toward specific proliferative or invasive-associated states, yet these states coexist within individual tumors and collectively shape phenotypic behavior^40^. Notably, invasive potential has been linked to specific transcriptional programs, including OPC-like and mesenchymal-like states, rather than to individual oncogenic alterations per se. Consistent with this framework, the diffusely invading models in our cohort express strong astrocyte marker signatures, while translocating cells in U3013MG are mesenchymal in character^37^. Protein-level analyses further suggest that invasion phenotype classes differ in the expression of markers including IFI16 and MEF2C, though the mechanistic significance of these associations remains to be established. Together, these observations support a model in which the spatial organization of proliferative and invasive dynamics reflects the distribution and plasticity of transcriptional cell states, modulated, but not dictated by tumor genotype. GlioTrace captures the behavioral output of this state landscape in real time, providing a dynamic complement to static transcriptomic snapshots that cannot resolve how state heterogeneity produces spatially organized invasion.

GlioTrace also provides a quantitative framework for assessing how pharmacological perturbations reshape invasion dynamics at the level of individual state transitions. Treatment with dasatinib, a SRC-family kinase inhibitor, selectively reduced transitions into highly motile translocating states while increasing the fraction of cells occupying round, low-motility states, consistent with the known role of SRC signaling in cytoskeletal remodeling and adhesion dynamics required for directed migration^22,41^. In contrast, thapsigargin, which perturbs calcium homeostasis and activates ER stress pathways^42^, produced a distinct perturbation signature, indicating that GlioTrace captures mechanistically meaningful differences between pharmacological agents rather than nonspecific effects on cell viability. Standard-of-care treatment with radiation combined with temozolomide had comparatively modest effects on migration speed but produced a consistent increase in the fraction of branching cells across models. Because branching cells occupy a central hub position in the transition network and display the highest migratory capacity, this enrichment raises the possibility that branching represents a treatment-tolerant invasive phenotype that persists following depletion of more therapy-sensitive populations, potentially contributing to the near-universal pattern of local recurrence in glioblastoma. Whether this enrichment reflects selection, phenotypic reprogramming, or both remains to be determined, but the observation motivates direct investigation of branching states as targets for combination strategies pairing cytotoxic and anti-invasive agents.

The ability to quantify patient-specific invasion dynamics and their pharmacological modulation positions GlioTrace as a functional phenotyping platform with several potential translational applications. Most immediately, it serves as a discovery tool for characterizing invasion behavior across larger patient-derived model collections, identifying reproducible dynamic subgroups not captured by static molecular profiling, and prioritizing candidate anti-invasive agents for further preclinical development. In a more translational setting, tumor slices prepared directly from surgical resection material could in principle be subjected to ex vivo GlioTrace profiling, enabling functional assessment of invasion dynamics and drug sensitivity within a timeframe compatible with clinical decision-making. Unlike traditional organotypic co-culture approaches, in which invasion dynamics are dominated by the geometry of the injection site, GlioTrace operates on tumors established in vivo, preserving a realistic spatial distribution of tumor cells across anatomical structures^26^. Consistent with this, directly transplanted preparations were enriched for round cells and displayed reduced migratory activity, suggesting they less faithfully reproduce the invasion dynamics of established tumors (Supplementary Fig. 16A–C; Supplementary Movie 6). Longer term, a realistic path toward clinical utility likely runs through biomarker discovery. By identifying molecular correlates of dynamic invasion states, it may become possible to infer invasion phenotype composition from standard clinical specimens without requiring live imaging infrastructure. GlioTrace is therefore best understood at this stage as a discovery platform whose translational potential is supported by the present findings but will require systematic validation to realize. To facilitate adoption, GlioTrace software is distributed as an open-source Python package compatible with standard confocal time-lapse data. The project repository (Code availability) provides full documentation, a step-by-step pipeline flowchart, two jupyter notebooks demonstrating example use cases with parameter combinations for each step, and example datasets hosted on Zenodo, including a demonstration of tracking and state-morphology visualization on an external dataset (Supplementary Movie 8).

Several limitations of the current study point toward productive directions for future work. The PDCX models are established in immunodeficient mice lacking functional T and B lymphocytes, and therefore do not capture adaptive immune interactions present in human glioblastoma. Microglial activation states in this system may accordingly differ from those in immunocompetent tumors, where CD8⁺ T cell contribute to shaping tumor-associated myeloid phenotypes at the invasive edge, as illustrated by our observation that TREM2-positive microglia are confined to the tumor core in our system, in contrast to their reported enrichment at the invasive edge in human GBM^43^. Extending GlioTrace to syngeneic or humanized models will be important for capturing these interactions. The brain slice assay introduces surface injury that contributes to early cell death measurements, although major brain cell populations and transcriptional programs remain stable across the imaging window, and the air-liquid interface does not recapitulate the blood-brain barrier - an important consideration when interpreting pharmacological responses to agents whose efficacy in vivo depends on CNS penetration. The current implementation is restricted to two fluorescence channels and maximum intensity projections rather than full three-dimensional volumes, and the cohort of six PDCX models, while spanning major invasion architectures, is insufficient for statistically powered subtype-level conclusions. These constraints define a clear path forward: expansion to larger and more immunologically representative model cohorts, multi-channel labeling strategies incorporating neurons, astrocytes, and immune populations, and full three-dimensional tracking will substantially extend the biological scope of the framework. The morphology classifier achieves 87% accuracy across six morphological classes, substantially exceeding the 17% chance expectation; remaining classification uncertainty is propagated through the HMM framework. The vessel segmentation and microglial interaction classifiers were trained on relatively small labeled datasets, which may limit generalization to imaging conditions that differ from those used here. Users working with different microscopy setups may need to retrain any of these classifiers on locally acquired labeled data, and the modular Python implementation is designed to support this.

The current study characterizes six PDCX models selected to represent opposing ends of the GBM invasion spectrum; this cohort is sufficient to demonstrate patient-specific differences in invasion dynamics and drug response but is too small for statistically powered conclusions regarding associations between molecular subtype, driver alteration, and invasion phenotype. Larger panels will be required to establish whether reproducible dynamic invasion subgroups exist beyond the perivascular and diffuse extremes, and to identify molecular predictors of invasion plasticity. In addition, the ex vivo system used here does not directly capture clinical outcomes such as survival or recurrence. While the HGCC PDCX collection used in this study has been shown to reproduce clinically relevant histopathological invasion patterns, and xenograft survival correlates with patient survival in the broader HGCC Phenobank^37,44^, a direct link between GlioTrace-derived invasion dynamics and patient clinical trajectories remains to be established in prospective cohorts.

Ultimately, GlioTrace provides a quantitative framework for dissecting glioblastoma invasion at single-cell resolution, and linking the dynamic states identified here to their underlying molecular programs through spatial transcriptomics or conditional reporters represents an exciting next step toward understanding, and therapeutically targeting, invasion plasticity in glioblastoma.

## Methods

### Models used in experiments and subject details

The mouse experiments described herein were carried out in accordance with an ethical permit granted by the Uppsala Animal Research Ethical Board, Sweden (Ethical permit number: 5.8.18-06726/2020 and 5.8.18-00216/2023). Female mice from 5 weeks of age (Hsd: Athymic Nude-Foxn1^nu^, genetic background: Foxn1^nu^/Foxn1^nu^, source: Envigo, Netherlands) were housed in ventilated cages, each accommodating up to five mice. These cages were equipped with suitable paper-based enrichment and bedding material, while the mice had unrestricted access to food and water. Environmental conditions were controlled to adhere to a 12-h light/12-h dark cycle at an ambient temperature and humidity. Sex was not included as an experimental variable, as all animals were female for grouped housing condition and sex was not considered to the study objectives.

### GFP-tagged human glioblastoma patient-derived primary cancer cell cultures

Primary cultures of patient-derived tumor cell lines as outlined in Supplementary Dataset 1, sheet 1, were procured from the Human Glioblastoma Cell Culture (HGCC) biobank^45,46^. The collection of HGCC samples was authorized by the Uppsala Regional Ethical Review Board, Sweden (2007/353), and explicit informed consent was obtained from all participants. These cells were propagated in laminin-coated T25 flasks (Corning #353808) utilizing a serum-free neural stem cell culture (NSC^+/+^) medium, comprising 50:50 nutrient blend of Neurobasal and DMEM/F12 media (Gibco, #21103-049, #31331-028), supplemented with B27 and N2 additives (ThermoFisher Scientific, #12587-001, #17502-001), human recombinant epidermal growth factor (EGF) and fibroblast growth factor (FGF) at concentrations of 20 ng/ml each (Peprotech), along with 1% penicillin/streptomycin. All utilized cell cultures were previously transduced with Green Fluorescent Protein (GFP) and luciferase (pBMN(CMV-copGFP-Luc2-Puro), Addgene plasmid #80389)^47^. These cell lines are available from HGCC biobank under a material transfer agreement; requests should be directed to the corresponding author. Furthermore, all cell lines underwent scrutiny for mycoplasma contamination using the Mycoalert kit (Lonza, #LT07-418) and were affirmed to be devoid of such contamination.

### Xenotransplantation of patient-derived glioblastoma cells in mice

Each GFP-labeled HGCC line was prepared at a density of 1 million cells per 20 µl in phosphate-buffered saline. Mice aged 6 weeks were anesthetized using deep isoflurane inhalation within an induction chamber and subsequently secured onto a stereotactic frame under anesthesia. Subsequently, 100,000 tumor cells were stereotactically injected into the striatum of the mouse brain at the following coordinates relative to the bregma: AP + 0, ML 1.5(R), DV −3.0. Mice were closely monitored until fully awake, and post-injection weights were recorded to monitor weight changes throughout the experiment. A total of 56 mice were used across the six HGCC models.

### Glioblastoma PDCX tumor growth monitoring through bioluminescence

Animal ethics permits specified humane endpoints rather than a maximal permitted intracranial tumor burden. In compliance to the ethical permit, following 6 weeks post-implantation of glioblastoma into the striatum using stereotactic techniques, mice received an intraperitoneal injection of 100 µl of luciferin solution (15 µg/ml), followed by in vivo IVIS spectrum imaging (Perkin Elmer, Waltham MA) every 2 weeks to quantify luciferase signal levels indicative of optimal tumor growth. Tumor-bearing mice were monitored at least three times weekly and were generally euthanized before reaching predefined humane endpoints: loss of ≥15% of maximum body weight, or reduced activity, whichever occurred first. Endpoint IVIS signals for individual mice are provided in Supplementary Dataset 1, sheet 1.

### Vibratome sectioning and PDCX brain slice culture

Intermittently, when luciferase levels indicated optimal tumor growth, mice were euthanized individually, and their brains were aseptically extracted and submerged in 50 ml of cold HBSS medium (Gibco™, #24020117) to facilitate preparation for vibratome sectioning. The brain was then encased in a low-melting agarose-HBSS medium cube. Organotypic brain slice cultures, each 300 µm in thickness, were generated through coronal sectioning of the brain utilizing a Leica vibratome (Leica VT 1200 S) instrument, with real-time sectioning speed adjusted between 0.2 and 20 µm/s to accommodate variations in brain texture, particularly in areas affected by tumor growth. Subsequently, brain slices were immersed in ice-cold brain slice culture medium (NSC+/+, 2.5 mM HEPES, 10 mM glucose) within a 6 cm culture dish. These slices were then transferred onto transwell membranes in a 12-well plate (Corning, #3460), with each well containing 700 µl of brain slice culture medium. Excess medium surrounding the brain slices on the transwell was carefully removed to maintain the air-liquid interface necessary for optimal brain slice culture conditions. The brain slice cultures were then placed in a 5% CO_2_ incubator at 37 °C for 24 h to recover and acclimate from any physical trauma incurred during the slicing process.

### Time-lapse whole brain slice imaging and post-acquisition processing

Time-lapse Z-stack images of brain slices were captured using the ImageXpress Micro Confocal High-Content Imaging System (IXMCS) (Molecular Devices), with imaging parameters set to a Z-step size of 10 µm, 9 × 9 tiles, a 10X objective with calibration of 1.36 µm/pixel. These parameters were adjusted to ensure optimized imaging frequency, with intervals of no more than 2 h between frames. The brain slice culture medium was replenished every 48 h, and imaging sessions continued for up to 5 days. Subsequently, all brain slice culture images from each experiment underwent processing and montage creation using the montage and overlay journal in MetaXpress 6.5 software (Molecular Devices). Following image tiling, sequences were stack registered and aligned for frame-to-frame coherence using custom-written MATLAB R2023b (The MathWorks) scripts, facilitating statistical analysis of cell dynamics and tumor growth.

### Tomato lectin-DyLight 594 conjugate bioavailability assay

The bioavailability of the compounds in slice cultures was evaluated by a tissue penetrant conjugate TL-DyLight 594 (ThermoFisher Scientific, #L32471). Brain slices were incubated with brain slice culture medium containing TL-DyLight 594 at a concentration of 2 µg/ml. Subsequent to the addition of TL-DyLight 594, time-lapse images were captured at intervals of 1.4 h over a 24-h period.

### Evaluation of slice viability

To assess PDCX slice viability, brain slices underwent culturing for 0, 1, 3, and 6 days. Following each incubation period, slices were incubated with 5 µM Hoechst 33342 (ThermoFisher Scientific, #62249) in the brain slice culture medium, a cell-membrane permeable dye that labels all cells in the sample, and 2 µM EthD-1 (Sigma-Aldrich, #46043-1MG-F), a live cell-impermeable dye that selectively labels dead cells, was applied for 45 min at room temperature (RT) by gentle shaking. After incubation, brain slices were washed with fresh brain slice culture medium and imaged using the IXMCS. The slice viability was quantified as the percentage of EthD-1 positive cells out of the total number of Hoechst-positive cells for 2D projection images saved as maximum projection of 15 z-stacks taken at 10 µm interval.

### Drug and standard of care treatments

For the in vitro drug treatment studies, four HGCC cell lines (Supplementary Dataset 1, sheet 1) were seeded at a density of 2500 cells per well in a 96-well Primaria plate (Corning, #353872) coated with laminin and grown in a 5% CO_2_ incubator at 37 °C. The next day, dasatinib (Selleck Chemicals, #S1021) and thapsigargin (Sigma-Aldrich, #T9033) treatments were added in the concentration range of 0.005, 0.05, 0.5, 1, 2, 4, 8, 16, 32, and 50 µM. Plates were then immediately transferred into an Incucyte S3 (Sartorius) for live cell image acquisition every 30 min for 72 h.

For the PDCX brain slice culture treatment studies, slices were treated with dasatinib and thapsigargin at a concentration of 32 µM and 4 µM, respectively, prepared in brain slice culture medium. For a dasatinib dose escalation treatment PDCX brain slices were treated with 0.05, 1, 4, 8, 16 and 32 µM dasatinib. For the thapsigargin dose escalation treatment, PDCX brain slices were treated with 0.05, 1, 2, and 4 µM thapsigargin. For the microglia depletion PDCX slices were treated with 50 and 100 µM Clodronate (Sigma-Aldrich, #D4434-1G). Soon after adding the drug treatment, slices were transferred to the IXMCS system for time-lapse image acquisition.

For the single-cell RNA transcriptomics, slices were incubated with or without 32 µM dasatinib treatment and incubated for 1 day, 3 days, and 6 days.

### Ex/in vivo radiation treatments

For the ex vivo radiation treatment, PDCX slices were treated with different doses of radiation as mentioned in the Supplementary Dataset 1, sheet 1. For the in vivo radiation treatment, tumor-bearing mice were treated with 4 Gy of radiation per day for the 5 consecutive days (total radiation 20 Gy). Soon after the last dose of radiation, brain was removed, sliced and slices were cultured with or without temozolomide (400 µM) treatment. The time lapse images were acquired in IXMCS for 5 days.

### Tissue dissociation and single-cell isolation from PDCX brain slices

The four slices per sample (each slice from different brain of U3013MG PDCX) for each time point were pooled together using cold HBSS buffer containing 1% Pen/Strep, 0.6% glucose, and 25 nM HEPES. The tissues of brain slices were dissociated into single cells by mixing with 1 ml micropipette tip and using tissue dissociation kit, human and mouse (Miltenyi, #130-095-942) according to the manufacturer’s protocol. Red blood cells were removed using the Red Blood Cell Lysis Solution (Miltenyi, #130-094-183). The myelin debris was removed using myelin removal kit (Miltenyi, #130-096-733).

### Single-cell RNA sequencing

The single-cell RNA sequencing libraries were prepared according to the manufacturer’s guidelines, following the Chromium GEM-X Single Cell 3’ Kit v4. (10X Genomics, #PN-1000686). Following cell isolation from PDCX slices, cells were washed and resuspended in 1X PBS and loaded onto Chromium GEM-X Single Cell 3´chip (10X Genomics, #PN-1000690) aiming to recover 20000 cells per sample. The quality of the libraries was assessed using Agilent High Sensitivity D5000 Kit (Agilent Technologies, #5067-55(92,93,94)). The libraries were sequenced on Illumina NovaSeq X Plus with the configurations in accordance with 10X Genomics recommendations. A total of six samples were sequenced and profiled. Each sample contained cells isolated from four slices pooled from four individual mouse brains for control and treatment group at each time points (days 1, 3, 6).

### Slice H&E, immunohistochemistry and immunofluorescence staining

Upon completion of time-lapse image acquisition, brain slices were fixed with 1–2 ml of freshly prepared 4% paraformaldehyde (PFA) for 2 h at room temperature (RT) and subsequently transferred into a 24-well plate. Following a triple wash with phosphate-buffered saline (PBS), the brain slices were subjected to incubation in a blocking solution (comprising 3% normal goat serum and 0.3% Triton X-100 in PBS) for 2 h at RT. Thereafter, slices were exposed to the following primary antibodies prepared in the blocking solution: mouse anti-STEM121 (diluted 1:500; Takara Bio, #Y40410; lot: AL90014S), rabbit anti-Ki67 (diluted 1:300; Abcam, #ab15580; lot: 1130217-1, GR123630-1), rabbit anti-cleaved caspase 3 (1:300; CST; #9664S; lot: 22), mouse anti-cleaved caspase 3 (1:200; Proteintech, #68773-1-Ig; lot: 10041968), mouse anti-Iba1/AIF1 (1:250; EMD millipore, #MABN92; lot: 3869128), rat anti-mouse CD13 (1:200; BIO-RAD, #MCA2183GA; lot: 158816), Goat anti-mouse/rat CD31/PECAM-1 (1:100; R&D Systems, #AF3628; lot: YZU0326011) rabbit anti-TREM2 (1:100; Abcam, #AB305103; lot: 1125056-2), rabbit anti-GFAP (1:250; CST; #80788T; lot: 4), rabbit anti-beta-3 tubulin (1:300; Invitrogen; #PA5-85639; lot: 79676988), rat anti- mouse CD11b (1:250; Invitrogen; #14-0112-82; lot: 3161275). Following a 2-day incubation period, the slices underwent triple PBS washes and subsequent incubation with appropriate Alexa Fluor-conjugated antibodies at 1:500 dilution (Goat anti-mouse Alexa Flour 488, Invitrogen #A32723, lot: ZF391806; Goat anti-rabbit Alexa Flour 647, Invitrogen #A21244, lot: 2134003; goat anti-rat Alexa Flour 647, Invitrogen #A21247, lot: 3148282; donkey anti-goat Alexa Flour 647, Invitrogen #A21447, lot: 3219279) for 4–5 h, followed by another triple PBS wash. Hoechst staining was then performed for 30 min.

Immunohistochemistry, hematoxylin and eosin staining performed as per standard protocol described previously with following modifications^37,44^. Mouse brains were embedded in paraffin through an automated tissue processor (70% ethanol, 1 h; 96% ethanol, 2 × 1 h; 100% ethanol, 3 × 1 h; xylene, 2 × 1 h; paraffin at 60 °C, 3 × 1 h), and sectioned at 5 μm. PFA fixed PDCX brain slices were embedded in paraffin with histogel in CellSafe+ biopsy cassettes (Histilab, #41130) through tissue processor (70% ethanol, 1 h; 96% ethanol, 1 h; 100% ethanol, 1 h; xylene, 1 h; paraffin at 60 °C, 1 h), and sectioned at 4 μm. Sections were deparaffinized with Xylene and rehydrated before hematoxylin and eosin staining. Antigen retrieval was performed in citrate-based Antigen Unmasking Solution (pH 6; Vector Laboratories, #H-3300) with 0.05% Tween 20 (Bio-Rad, #1610781) in PBS, followed by cooling to room temperature. Endogenous peroxidase was quenched with 3% H₂O₂ in PBS (10 min), and sections were blocked with Normal Antibody Diluent (ImmunoLogic WellMed, #UD09) for 30 min. Primary antibodies mouse anti-STEM121 (1:500; Takara, #Y40410, lot: AL90014S), rabbit anti-Numa (1:300; Abcam, #ab97585; lot:GR268490-35), were incubated for 60 min at 4 °C overnight. Detection used BrightVision one-step HRP systems (anti-rabbit, #DPVR110HRP; anti-mouse, #DPVM110HRP; WellMed) with Bright-DAB substrate (WellMed, #KBS04-110). Sections were counterstained with Mayer’s Hematoxylin and mounted with Pertex (HistoLab, #00811).

### Image registration and ROI selection

The input data from the IMX system were provided as 9000×9000 whole slide scans for a sequence of time points 1, 2,…, T. The image sequences were stabilized using rigid (translation + rotation) affine transformations based on automatically extracted landmarks^48^. Regions of interest (ROIs) were extracted from the slice to create stacks of size *W* × *W* × *T*, where *W* is the size of the region. ROI selection was followed by stabilization using an efficient rigid stack registration algorithm based on a 2-dimensional Fast Fourier transform^49^.

### Detecting tumor cell proliferation and movement

#### ROI-level summary measures

As a first layer of quantification, we computed ROI-level measures of growth and movement. To measure growth, a single region was chosen for each slice that would encompass the full tumor region (unless multiple tumor sites were present in the slice, in which case multiple ROIs were selected to accurately capture the full tumor). For each stack or collection of stacks representing the whole tumor, the total pixel intensity of the green channel in each frame is normalized by region size and divided by the total pixel intensity of the first frame in the stack to generate a sequence of values of length T, corresponding to the percentual increase in green channel signal over time. To detect movement, multiple smaller regions from each slice were chosen. Regions were primarily selected to be suitable for single-cell tracking, avoiding tumor bulk or empty regions. For each stack from the peripheral tumor regions in the slice a time-resolved adjusted mean of absolute differences (adMAD) was calculated according to Eq. 1 where g is the green channel in stack G of size W x W x T. The absolute difference in mean pixel intensity between two frames was normalized by the mean pixel intensity of the two frames together to adjust for an increasing number of cells in the frame over time. The resulting measure describes the rearrangement of pixels between frames over time.

1 $${adMAD}\left(i\right)=\frac{\overline{{|g}\left(:,:,t+1\right)-g\left(:,:,t\right)|}}{\overline{g\left(:,:,t+1\right)+g\left(:,:,t\right)}}$$

#### Cell tracking

To track single cells, we continued working with the peripheral ROIs suitable for this goal. Individual cell center detection was combined with a Kalman filter-based framework for multi-object tracking. Cells were detected with means of common image analysis operations implemented in Python^50–53^ intended to find intensity peaks in an image that could correspond to cell centers (Algorithm 1). Generated peaks were filtered through a number of steps to ensure that peaks (coordinates) were located to cell bodies (not cell protrusions), that they corresponded to single cells and that one cell had only one coordinate. The pruning of candidate points was followed by feature extraction; for each coordinate an image of window size *B* × *B* × *C* was extracted from the original image stack frame, where *B* is the size in pixels and *C* is the number of channels.

Cell coordinates and corresponding images are passed on to a multi-object tracking framework (Algorithm 2). The tracking framework is based on the Kalman filter algorithm which connects spatial coordinates across time to create object trajectories. It relies on assumptions of an object’s velocity and acceleration, the dynamic model. Based on the dynamic model, coordinates are extrapolated into the near future and become predictions. The predictions are used to match coordinates between two subsequent frames by solving a linear assignment problem. The resulting tracks consist of coordinates x(t), y(t).

3. Summary statistics. From the tracks, we computed a number of summary statistics. These were the average speed (microns per hour) averaged across the whole track and the turning angle distribution obtained by standard trigonometry functions. Furthermore, the mean squared displacement (MSD) was calculated from the cell trajectory coordinates. For each stack, every cell trajectory is handled separately to calculate the squared displacement for each coordinate from the starting point of the trajectory. The tracks are interpolated to the smallest **Δ**t and the final MSD is calculated as the ensemble mean across all trajectories from all stacks cell line-wise. Tracks are handled in such a way that they all start at t_0_, so that the time axis of the MSD curve corresponds to the time elapsed from the start of a track and not to the time elapsed in image acquisition.

### Detecting tumor cell morphologies and phenotypic switching

#### Morphological classifier

The images extracted around cell coordinates in the cell detection step were classified as belonging to five biological morphological classes (branching, diffuse translocation, locomotion, perivascular translocation, and round) and one additional crowded/outlier class used to capture ambiguous or overlapping cells (Supplementary Fig. 5), using a convolutional neural network (CNN) (Deep Learning Toolbox, Version 23.2, Release 2023b, The MathWorks, Inc., Natick, Massachusetts, United States)^54^.

The network was trained on 7800 manually curated images of the morphological classes. Class imbalance of the training data was handled through stratified splitting of the data into training, validation and test sets. In the training data, under-represented classes were handled by oversampling. Cell morphologies from all six patient-derived models were represented in the dataset. To test the cross-patient generalizability of the learned representations, we performed leave-out-patient-out cross-validation, confirming consistently high per-class per-patient recall across models, with the only exceptions being very low example counts for certain morphologies upon exclusion (Supplementary Fig. 5). Per-class recall was chosen in addition to overall accuracy to ensure that minority classes were not masked by high performance on dominant classes.

Mean subtraction centering was performed by subtracting the mean training image from all images in both training, validation and test set channel-wise. Mean subtraction centering with the same mean image was also performed channel-wise when images were classified inside the framework. Augmentation of training and validation images was achieved with the MATLAB function augmentedImageDatastore and included reflection, rotation and scaling.

To deploy the classifier in the GlioTrace package, an equivalent network architecture was reimplemented in Python (PyTorch) and the trained MATLAB weights were transferred layer-by-layer.

#### Covariate Hidden Markov Model (HMM) with GLM transitions and CNN-based emissions

By applying the above classifier to each cell along its track, we obtained at every time point *t* = 1,2,… a vector of scores over the 6 morphological classes (five biological classes and one outlier class, *K* = 6 from which we derive per-class probabilities (via softmax) and class assignments. For downstream analysis, predictions corresponding to the outlier class are excluded. Because these frame-wise predictions do not enforce temporal consistency and are noisy, we model the underlying morphological state using a Hidden Markov Model (HMM).

In the HMM, the hidden variable $Z\left(t\right)$ represents the latent (true) morphological class of a cell at time *t*, while the CNN output provides the observation evidence. The HMM model is defined by (i) an initial distribution *π* and (ii) a transition model *A* describing how hidden model states evolve over time.

Transition model: multinomial logistic regression. To allow transitions to depend on track-derived covariates, we model the probability of moving from one hidden state to another using multinomial logistic regression. Let $x\left(t\right)$ denote a vector of covariates measured from the track data at time *t*. The GlioTrace software offers the following covariates:

- Speed: Displacement between frames divided by dt
- Vascular distance: Distance to closest vasculature (see segmentation net below)
- Microglia associated: See trained network below
- Cosine similarity: Similarity between a cell’s direction and the average direction of all cells in the ROI
- Polarization: A measure of Co-movement defined as the length of the average unit vector of all cells in the ROI. A value of 1 indicates all cells move in the same direction and 0 indicate movement is uniformly distributed on all directions
- Treatment interaction: interaction terms for treatment against the covariates specified

For *K* = 1, 2…. the multinomial logistic regression $j={{\mathrm{1,2}}},\ldots,K-1$ is a linear predictor for the log-odds relative the baseline (2)

2 $$log \frac{\Pr \left(Y=j\right)}{\Pr \left(Y=K\right)}=x{\left(t\right)}^{T}{\beta }_{j}$$

where ${\beta }_{j}$ is a class-specific coefficient vector. This parametrization implies a proper probability distribution over classes, with $\Pr \left(Y=K\right)$ determined by normalization (3)

3 $$\Pr \left(Y=K\right)=1-{\sum }_{j=1}^{K-1}\Pr \left(Y=j\right).$$

Equivalently, we can formulate things in terms of probabilities for each class by for $j={{\mathrm{1,2}}},\ldots,K-1$(4)

4 $$\Pr \left(Y=j\right)=\frac{\exp \left(x{\left(t\right)}^{T}{\beta }_{j}\right)}{1+{\sum }_{h=1}^{K-1}\exp \left(x{\left(t\right)}^{T}{\beta }_{h}\right)}$$

and (5)

5 $$\Pr \left(Y=K\right)=\frac{1}{1+{\sum }_{h=1}^{K-1}\exp \left(x{\left(t\right)}^{T}{\beta }_{h}\right)}.$$

In our HMM we require probabilities of the form $\Pr (Z\left(t+1\right){|Z}\left(t\right),x\left(t\right))$. To achieve this, we fit one multilogistic linear regression model per from-state *i*. Concretely, for each hidden state *i*, the corresponding GLM takes as input $x\left(t\right)$ and outputs a probability distribution over all possible next states *j*. This yields a feature-based transition model ${{{\bf{A}}}}\left(t\right)$, where each row corresponds to a from-state.

The model parameters *β* are estimated by maximizing the log-likelihood; In our implementation we use scikit-learn’s multinomial logistic regression solver where the contribution of a cell at time *t* to each of the *K* multinomial logistic regression models is decided by the updated HMM class posterior probabilities.

Emission model: CNN derived observed likelihood (fixed).

For the emission component, we use the CNN outputs as observed evidence. Specifically, at each timepoint the CNN produces logits over the classes which we transformed into log-probabilities using a log-softmax function. These log-probabilities are used as emission log-likelihoods, i.e. they quantify how compatible the observed CNN evidence is with each hidden state.

HMM fitting and state scaling. Given observed sequences of multiple tracks, we estimate the transition model parameters and the initial state distribution using an expectation-maximization (EM) procedure (Baum-Welch style), while keeping the emission model fixed. In the E-step, we run a forward backward algorithm for each track to compute (i) $\gamma \left(t,i\right)$, the posterior probability that $Z\left(t\right)=i$ given the full track, and (ii) $\xi \left(t,i,j\right)$ the posterior probability of the transition $Z\left(t\right)=i$ to $Z\left(t+1\right)=j$. In the M-step, we update *π* using *γ* at the first time point, and refit each from state GLM using $x\left(t\right)$ as predictors and $\xi \left(t,i,j\right)$ as sample weights for the contribution to each model. After fitting we denoise the frame-wise classification by using the most likely hidden state sequence per track (Viterbi path), producing a final state call for each cell at each time point.

### Detecting microenvironmental interactions

#### Blood vessel disorganization

In matched tumor-free and tumor-peripheral regions, we quantified the length of the vasculature in every frame of the image stacks. To the red (TL-DyLight 594) channel of these stacks, we applied semantic segmentation by a U-net trained on 400 manually labeled 250×250 images of vasculature. Five-fold cross-validation gave an f1-score of 0.74 ± 0.015. The f1-metric was chosen because vessel pixels were a small minority of the total image pixels, making pixel accuracy misleading. The binary segmentation mask created by the network was skeletonized using the medial axis transform and pixels were summed up to calculate the length of the vasculature. The time-resolved measure of vasculature length was normalized to mean of control curves and to t_0_. From the curves, we computed a vascular disorganization index as the area under the curve, normalized by the stack-length.

#### Classifying tumor-microenvironmental interactions

To identify tumor cells colocalizing with microglia or migrating along blood vessels in the images, we developed a classifier using the same workflow as described above and a curated dataset of 3000 manually labeled images. The network scored the images as belonging to one of the two classes, thresholding the score with class-specific thresholds. Hence, if the classifier is too unsure of either class, the cell is classified as non-associated. The network achieved a performance of 92% accuracy on the test set (Supplementary Fig. 9K). The class sequences obtained were used for calculating the percentage of cells interacting with microglia and the average duration of this interaction.

### Assessing dose-dependent effects on cell statistics and morphological transition rates

#### Detecting patient differences and treatment effects

The above framework can be thought of as a nested design that results in summary statistics (speed, MSD, or morphological class frequencies) at the level of a particular cell, region of interest, slice, mouse brain, or patient, with treatment as an additional experimental factor. To perform statistical testing, we therefore use linear mixed effects models (Statistics and Machine Learning Toolbox, Version 23.2, Release 2023b, The MathWorks, Inc., Natick, Massachusetts, United States). To detect the effects of drugs we used (in Wilkinson notation) Y ~ treatment:dose + (1|batch), where each batch is a collection of slices cultured at the same time. We acknowledge that further study is needed to accommodate for non-linear responses to drugs and reserve such extensions to future work.

#### Differential transition matrices

As an additional explorative statistic, we calculated the effect of treatment and tumor microenvironment on the hidden transition matrix **A**, using the GLMs we obtained from the covariate HMM. For each patient specific model and treatment, the covariate HMM was first trained using the full data, which yielded a mean **A** matrix, by simply fitting the GLM models on the mean of the GLM feature values. To assess the variance of the **A** matrix, we performed jackknife resampling by training the HMM-GLM on the same data, excluding one mouse at a time. The results were aggregated through the mean and the jackknife variance (6)

6 $$\frac{M}{M-1}{\sum }_{i=1}^{M}{\left({x}_{i}-\bar{x}\right)}^{2}$$

of the probabilities, where *M* is the total number of mice, ${x}_{i}$ is the transition probability matrix of the jackknife sample excluding mouse *i*, and $\bar{x}$ is the mean jackknife transition probability matrix.

Further, we evaluated the effect of the treatment and the tumor microenvironment on the transition probabilities, by comparing the transition matrix of a low feature value to the transition matrix of a high feature value. For features such as treatment and microglia association, which are encoded as 0 or 1, we compared the GLM fitted on feature value = 0 to the same with feature value = 1. For the non-binary features, we instead compared the 10th percentile of the feature values to the 90th percentile. This was done for one feature at a time, with the others set to the mean of the feature values. Analogously to the above description, we performed jackknife resampling to assess the variance.

For all ex vivo experiments, the treatment-level analyses were done with matched control slices from the same mice as the treated slices. For in vivo experiments this was not possible due to the nature of the experiment, thus all other control slices from the same patient-derived model were used.

### Pre-processing, integration and clustering of single cell data

The Cell Ranger pipeline (v9.0.1)^55^ was used for demultiplexing and alignment of raw sequencing data. Single-cell analysis was performed using R^56^ in RStudio^57^, specifically using the Seurat package (v5.4.0)^58^. In the pre-processing step, we filtered out cells expressing less than 500 genes. Potential doublets were filtered away by removing cells expressing more than the 95th percentile of expressed genes per cell. We removed genes expressed in 10 cells or less. Cells with over 30% mitochondrial DNA were also removed. After these steps, 166,216 cells were left (93.2% of the original dataset). For species assignment, at least 70% of the reads in a cell had to map to either human or mouse; cells below this threshold were filtered out, removing an additional 461 cells. Cell cycle genes driving variance was regressed out by scoring for cell cycle gene sets for human and mouse using CellCycleScoring and SCTransform.

### Downstream analysis of single cell data

For clustering and cell type annotation of the mouse compartment, datasets were integrated using highly variable genes (HVG) to identify pairs of cells used for anchoring. Pairs were found by mutual nearest-neighbor matching in PCA space, computed from the HVGs across all datasets. Differences between pairs of anchor cells represent batch effects to be corrected. The corrected expression of each cell is the weighted combination of neighboring anchor corrections. After integration, cells were clustered using the Louvain algorithm on the shared nearest neighbor graph. After cluster assignment, all downstream analysis was performed on log-normalized (non-integrated) data. Clusters were annotated manually based on top marker genes for each cluster identified by running FindAllMarkers. Cells were scored for hallmark programs using GSEA Mouse MSigDB Hallmark gene sets.

Tumor cells were scored for hallmark GBM programs and visualized as described by ref. ^19^. For tumor cell state proportions across time points, cells were labeled according to the highest scoring module out of four meta modules: NPC, OPC, MES, AC (Nomura et al.^39^). The MES1/MES2 and NPC1/NPC2 signatures were merged in that case.

Differential gene expression analysis and GSEA were conducted using FindMarkers and gseGO with gene set size limits of 10–500, nPermSimple set to 10,000 and a *p*-value cutoff of 0.05.

### Inferring spatial differences in drug response from whole-slice analysis

To identify spatial trends in the drug response, we applied growth- and movement metrics to the entire slice, applying it to both tumor peripheral and core regions. Downsampling of the stack w.r.t. frame size was followed by Gaussian and Notch filtering. We estimated the growth across the slice as the median rate of log2 fold-change in GFP signal over time in the stack. Movement was estimated as the absolute difference between consecutive frames *t* + 1 – *t* normalized by abs(*t*) and averaged across time to create a mean absolute difference in GFP signal over time above some threshold.

### Cell number quantification in IHC stainings

For IHC stainings, we applied Gaussian filtering followed by blob detection by convolution with a Logarithm of Gaussian-filter corresponding to average cell size. Potential cell coordinates found in the blob detection were pruned by using the filtered, binarized version of the staining as a mask.

### Interpolation of data

When dealing with data from image stacks acquired with different time intervals between frames (Δt), data points were interpolated to the smallest Δt with the MATLAB function interp1.

#### Algorithm 1

Cell Center Detection and Feature Extraction.

For each frame f equals 1, 2, 3,…

1. Filter the green frame with the negative of Laplacian of Gaussian filter and identify peaks as local maxima against the 8 neighboring pixels.

2. Create an intensity-derived cell-body mask by computing an automatic global threshold on the green frame, binarizing the green frame, and then dilating with a small disk element.

3. Label connected components in the selected cell-body mask to obtain cell-body objects. Compute the area of each object and filter away those with too large area (area greater than 3000 pixels)

4. Apply strong Gaussian smoothing to the green frame using a large smoothing scale. Threshold the smoothed image to from a bulk mask. Treat bulk as present based on if the maximum smoothed intensity exceeds a value of 200 on 8 bit scale

5. For each cell-body object, collect all cell-center candidate coordinates as the coordinates which are local maxima, exceed a minimal threshold for noise, lie inside the object, lie inside the binarized green image mask and are not in the bulk mask.

6. If multiple candidates remain from step 6, choose the pixel closest to the mean coordinate of the candidates if the resulting pixel lies within the cell-body and else keep all coordinates.

7. Extract feature patches of size B by B around each retained coordinate in both the green and the red channel. Discard coordinates too close to the boundary.

#### Algorithm 2

Multi-object tracking using Kalman filter.

1. Initialize tracks in the first frame setting the track position to the detected cell centers and set the initial velocity to zero.

2. For each subsequent frame f = start+1, start+2, start+3,…

3. Predict the next position using a constant-velocity Kalman model.

4. Let q be the set of detections in the current frame. Pair detections to predicted track positions by solving a global minimum-cost assignment using Euclidean distance as the cost, with a hard constraint of 18 pixels (costs beyond 18 cannot get matched). Detections which are not paired remain unmatched.

5. For paired tracks, perform a Kalman measurement update using the matched detection.

6. For tracks not paired, keep the predicted position as the track position and update a missed-frame counter.

7. Tracks with missing frame > 2 are terminated

8. Detections not matched to any pre-existing track gets new tracks started from them.

9. Return x and y positions together with track ID

### Spatial analysis of phenotypic state organization

To quantify spatial organization of phenotypic states, we analyzed nearest-neighbor relationships between cells within each imaging frame. For each experiment, region of interest (ROI), and time point, cells were represented as spatial points using their tracked coordinates. Each cell was assigned a phenotypic state inferred by the hidden Markov model (HMM) classifier (viterbi_state, six discrete states).

For every cell in a frame, we identified the nearest neighboring cell using Euclidean distance in the image coordinate system. We then tabulated the joint frequencies of focal cell state i and nearest-neighbor state j, resulting in a 6 × 6 nearest-neighbor mixing matrix Mij. The rows of this matrix represent the focal cell state, and columns represent the state of its nearest neighbor. Row-normalized matrices therefore represent the conditional probability P(neighbor state=j|focal state=i).

To assess whether observed spatial associations differed from random spatial mixing of states, we generated a permutation-based null model. Within each frame, cell positions were held fixed while state labels were randomly permuted among cells. This procedure preserves the spatial configuration and the abundance of each state within the frame but removes any spatial dependence of state identity. For each dataset, we generated 500–1000 permutations and recomputed the mixing matrix for each permutation. Spatial enrichment or depletion of state pairs was quantified using Z-scores (7)

7 $${Z}_{{ij}}=\frac{{M}_{{ij}}-{\mu }_{{ijnull}}}{{\sigma }_{{ijnull}}}$$

where ${\mu }_{{ijnull}}$ and ${\sigma }_{{ijnull}}$ are the mean and standard deviation of the mixing counts obtained from the permutation distribution. Positive Z-scores indicate that state i cells have state j neighbors more often than expected under random mixing, whereas negative values indicate spatial avoidance.

To summarize homotypic spatial organization, we additionally extracted the diagonal elements ${Z}_{{ii}}{Z}_{{ii}}$, which quantify enrichment of same-state nearest neighbors. Cross-state interactions were visualized using directed networks in which nodes represent phenotypic states and edges indicate enriched nearest-neighbor preferences between different states.

### Statistics and reproducibility

We performed at least two independent experiments for the reproducibility with minimum two biological replicates. The individual slices as technical replicates from different mice brains representing biological replicates used for the specific experiment, are provided for each cell line in Supplementary Dataset 1. Statistical testing was performed with Welch’s two-sample t-test (two-tailed) or one-way ANOVA when involving multiple pairwise comparisons, adjusting for multiple pairwise comparisons using Tukey’s HSD test with a significance level of 0.05.

### Reporting summary

Further information on research design is available in the Nature Portfolio Reporting Summary linked to this article.

## Supplementary information

Supplementary Information

Description of Additional Supplementary Files

Supplementary Dataset 1

Supplementary Movie 1

Supplementary Movie 2

Supplementary Movie 3

Supplementary Movie 4

Supplementary Movie 5

Supplementary Movie 6

Supplementary Movie 7

Supplementary Movie 8

Reporting Summary

## Source data

Source Data

Transparent Peer Review file

## Data Availability

The single-cell RNA sequencing data generated in this study have been deposited in the Gene Expression Omnibus (GEO) database under accession code GSE344470. (https://www.ncbi.nlm.nih.gov/geo/query/acc.cgi?acc=GSE344470). A subset of the time-lapse imaging dataset generated in this study (U3028MG; baseline, dasatinib- and thapsigargin-treated) has been deposited in Zenodo under (https://doi.org/10.5281/zenodo.21981544). Clinical and genomic annotations for the patient-derived cultures used in this study are available from the HGCC resource (https://hgcc.se) and are provided in Supplementary Dataset 1, sheet 2. All remaining data supporting the findings of this study are available within the article, its Supplementary Information and its Source Data file. Source data are provided with this paper.

## Code availability

The full code base for the GlioTrace framework and additional image processing scripts are available as a GitHub repository under an Apache-2.0 licence (https://github.com/Gliomethods/GlioTrace.git), and the release (v0.2.0) used in this study is archived in Zenodo^59^.
