# Supplementary material for: Reconstructing the single-cell spatiotemporal dynamics of glioblastoma invasion
Source: Nat Commun. 2026 Nov 2;17:11245. doi: 10.1038/s41467-026-78908-9 (PMC13728213; doi:10.1038/s41467-026-78908-9)
Supplement: Supplementary file 1 [file 41467_2026_78908_MOESM12_ESM.pdf]

nature portfolio

Corresponding author(s): Sven Nelander, Ph.D.

Last updated by author(s): 2026-08-

Reporting Summary

Nature Portfolio wishes to improve the reproducibility of the work that we publish. This form provides structure for consistency and transparency in reporting. For further information on Nature Portfolio policies, see our Editorial Policies and the Editorial Policy Checklist.

Statistics

For all statistical analyses, confirm that the following items are present in the figure legend, table legend, main text, or Methods section.

n/a	Confirmed	
☐	☒	The exact sample size (*n*) for each experimental group/condition, given as a discrete number and unit of measurement
☐	☒	A statement on whether measurements were taken from distinct samples or whether the same sample was measured repeatedly
☐	☒	The statistical test(s) used AND whether they are one- or two-sided<br>*Only common tests should be described solely by name; describe more complex techniques in the Methods section.*
☐	☒	A description of all covariates tested
☐	☒	A description of any assumptions or corrections, such as tests of normality and adjustment for multiple comparisons
☐	☒	A full description of the statistical parameters including central tendency (e.g. means) or other basic estimates (e.g. regression coefficient) AND variation (e.g. standard deviation) or associated estimates of uncertainty (e.g. confidence intervals)
☐	☒	For null hypothesis testing, the test statistic (e.g. *F*, *t*, *r*) with confidence intervals, effect sizes, degrees of freedom and *P* value noted<br>*Give P values as exact values whenever suitable.*
☒	☐	For Bayesian analysis, information on the choice of priors and Markov chain Monte Carlo settings
☒	☐	For hierarchical and complex designs, identification of the appropriate level for tests and full reporting of outcomes
☐	☒	Estimates of effect sizes (e.g. Cohen's *d*, Pearson's *r*), indicating how they were calculated

Our web collection on statistics for biologists contains articles on many of the points above.

Software and code

Policy information about availability of computer code

Data collection	All images were acquired using IXMC and controlled and processed by MetaXpress 6.5 software (Molecular Device, Inc.). Tumor bearing IVIS images was collected using Living Imae 4.8.2 software from Revvity.
Data analysis	All image analysis was performed in the MATLAB R2023b with Deep Learning Toolbox v23.2 and Statistics and Machine Learning Toolbox v23.2 (MathWorks) and Python 3.11, R and RStudio, and Cell Ranger. Adobe Illustrator 28.1 and Affinity designer 2 were used for figure assembly. The code availability statement in the manuscript now reads as follow: The full code base for the GlioTrace framework and additional image processing scripts are available as a GitHub repository under an Apache-2.0 licence (https://github.com/Gliomethods/GlioTrace.git), and the release (v0.2.0) used in this study is archived in Zenodo.

For manuscripts utilizing custom algorithms or software that are central to the research but not yet described in published literature, software must be made available to editors and reviewers. We strongly encourage code deposition in a community repository (e.g. GitHub). See the Nature Portfolio guidelines for submitting code & software for further information.

Data

Policy information about availability of data

All manuscripts must include a data availability statement. This statement should provide the following information, where applicable:

- Accession codes, unique identifiers, or web links for publicly available datasets
- A description of any restrictions on data availability
- For clinical datasets or third party data, please ensure that the statement adheres to our policy

The complete data availability statement is provided in the manuscript at the end of the methods section as follows: The single-cell RNA sequencing data generated in this study have been deposited in the Gene Expression Omnibus (GEO) database under accession code GSE344470. (https://www.ncbi.nlm.nih.gov/geo/query/acc.cgi?acc=GSE344470). A subset of the time-lapse imaging dataset generated in this study (U3028MG; baseline, dasatinib- and thapsigargin-treated) has been deposited in Zenodo under (https://doi.org/10.5281/zenodo.21981544). Clinical and genomic annotations for the patient-derived cultures used in this study are available from the HGCC resource (https://hgcc.se) and are provided in Supplementary Data, sheet 2. All remaining data supporting the findings of this study are available within the article, its Supplementary Information and its Source Data file. Source data are provided with this paper.

Research involving human participants, their data, or biological material

Policy information about studies with human participants or human data. See also policy information about sex, gender (identity/presentation), and sexual orientation and race, ethnicity and racism.

Reporting on sex and gender	The patient donor sex of each patient-derived cultures (U3013MG, female; U3028MG, female; U3054MG, female; U3220MG, female; U3179MG, male; U3180MG, male), per-culture clinical and genomic annotations are given in Supplementary Data, sheet 2.
Reporting on race, ethnicity, or other socially relevant groupings	Race, ethnicity, and other socially relevant groupings were not collected or reported for patient donors, as they were not relevant to this study.
Population characteristics	Patient donors ranged from 60 to 80 years of age. Transcriptional subtypes, major driver alterations, and MGMT methylation status for all six models are provided in Supplementary Data, sheet 2.
Recruitment	All human patient-derived cell lines were obtained from the Human Glioblastoma Cell Culture (HGCC) biobank at Uppsala University (https://hgcc.se).
Ethics oversight	All patient derived primary HGCC cell lines were handled as approved by the Uppsala Regional Ethical Review Board, Sweden, approval 2007/353, for the collection of human tumour material for the HGCC biobank.

Note that full information on the approval of the study protocol must also be provided in the manuscript.

Field-specific reporting

Please select the one below that is the best fit for your research. If you are not sure, read the appropriate sections before making your selection.

☒ Life sciences ☐ Behavioural & social sciences ☐ Ecological, evolutionary & environmental sciences

For a reference copy of the document with all sections, see nature.com/documents/nr-reporting-summary-flat.pdf

Life sciences study design

All studies must disclose on these points even when the disclosure is negative.

Sample size	A total of 56 mice were used in this study (21 mice for U3013MG, 10 mice for U3180MG, 7 mice for U3220MG, 8 mice for U3028MG, 4 mice for 3054MG, 2 mice for U3179MG, 4 mice for U3013MG and U3180MG cells transplantation experiments). No statistical method was used to predetermine sample size. Group sizes were chosen on the basis of our previous work with patient-derived xenograft and slice-culture models and on the practical constraint of the number of brain slices obtainable per tumour-bearing brain. Each condition comprised at least three independent tumour-bearing mice (except U3179MG, n = 2 mice, which is flagged in the text) with multiple slices per brain.
Data exclusions	No experiments were excluded on the basis of failed replication; the only data excluded were individual image regions failing pre-specified quality criteria as described in the Methods.
Replication	All attempts at experiment replication were successful. Every reported phenotype was reproduced across independent slices and independent tumour-bearing mice within each patient-derived model, and the patient-specific ranking of invasion metrics was reproducible across slices and mice.
Randomization	Covariates were controlled both experimentally and statistically. Experimentally, for all ex vivo perturbations, treated and control slices were prepared from the same brain and cultured and imaged in the same session and on the same plate, so that mouse, batch, slice-preparation and imaging conditions are matched within each comparison. Statistically, mice ID was included as random effects in the linear mixed-effects models (Y ~ treatment:dose + (1|mouse)), and the covariate hidden Markov model explicitly conditions transition probabilities on measured

microenvironmental covariates: vascular distance, microglial colocalisation and polarisation. For the in vivo irradiation experiment, matched same-brain controls were not possible; control slices from other mice of the same patient-derived model were used instead, and this is stated in the Methods.

Blinding

Investigators were not blinded during data collection, because the treated and control slices had to be identified in order to apply the correct compound and to place them in the correct plate wells. Blinding was not required for the primary analyses because all quantitative outputs: cell detection, tracking, morphological classification, vessel segmentation and transition modelling, were produced by automated algorithms applied identically and with identical parameters to all conditions, with no manual selection of cells or events. Regions of interest were selected on the basis of cell density, using pre-specified criteria, before any quantification.

Reporting for specific materials, systems and methods

We require information from authors about some types of materials, experimental systems and methods used in many studies. Here, indicate whether each material, system or method listed is relevant to your study. If you are not sure if a list item applies to your research, read the appropriate section before selecting a response.

Materials & experimental systems

n/a	Involved in the study
☐	☒ Antibodies
☐	☒ Eukaryotic cell lines
☒	☐ Palaeontology and archaeology
☐	☒ Animals and other organisms
☐	☒ Clinical data
☒	☐ Dual use research of concern
☒	☐ Plants

Methods

n/a	Involved in the study
☒	☐ ChIP-seq
☒	☐ Flow cytometry
☒	☐ MRI-based neuroimaging

Antibodies

Antibodies used

All antibodies used in this study are as listed below also mentioned in the methods section of the manuscript.
1. mouse anti-STEM121 (diluted 1:500; Takara Bio, #Y40410; lot: AL90014S),
2. rabbit anti-NuMa (diluted 1:300; Abcam, #ab97585, lot: GR268490-35),
3. rabbit anti-Ki67 (diluted 1:300; Abcam, #ab15580; lot: 1130217-1, GR123630-1),
4. rabbit anti-cleaved caspase 3 (1:300; CST; #9664S; lot: 22),
5. mouse anti-cleaved caspase 3 (1:200; Proteintech, #68773-1-Ig; lot: 10041968),
6. mouse anti-Iba1/AIF1 (1:250; EMD millipore, #MABN92; lot: 3869128),
7. rat anti-mouse CD13 (1:200; BIO-RAD, #MCA2183GA; lot: 158816),
8. Goat anti-mouse/rat CD31/PECAM-1 (1:100; R&D Systems, #AF3628; lot: YZU0326011)
9. rabbit anti-TREM2 (1:100; Abcam, #AB305103; lot: 1125056-2),
10. rabbit anti-GFAP (1:250; CST; #80788T; lot: 4),
11. rabbit anti-beta-3 tubulin (1:300; Invitrogen; #PA5-85639; lot: 79676988),
12. rat anti- mouse CD11b (1:250; Invitrogen; #14-0112-82; lot: 3161275);
13. Goat anti-mouse Alexa flour 488, 1:500, Invitrogen #A32723, lot: ZF391806;
14. Goat anti-rabbit Alexa Flour 647, 1:500, Invitrogen #A21244, lot: 2134003;
15. goat anti-rat Alexa Flour 647, 1:500, Invitrogen #A21247, lot: 3148282;
16. donkey anti-goat Alexa flour 647, 1:500, Invitrogen #A21447, lot: 3219279

Validation

All primary antibodies specificity were validated in house by IHC PDCX brain sections as well as fixed PDCX brain slices. Representative images confirming expected staining patterns are provided in the supplementary figure 1E (mouse anti-STEM121), 2 (rabbit anti-NuMa), 3A-D, 13C (rabbit anti-Ki67, rabbit anti-Cleaved caspase 3), 4A (rabbit anti-GFAP, mouse anti-cleaved caspase 3), 4B (rabbit anti beta-3-tubulin), 4C (rabbit anti-CD11b), 4D (rat anti-mouse CD13), 4E (goat anti-mouse/rat CD31), 10A, 10C, 10D (mouse anti-Iba1/AIF1), 10B (rabbit anti-TREM2). Additionally, the published references where the primary antibodies were used and validated are provided below for each primary antibodies from manufacturer's citation list.

1. Doroszko, M., et al., The invasion phenotypes of glioblastoma depend on plastic and reprogrammable cell states. Nat Commun, 2025. 16(1): p. 6662.
2. Filbin, M.G., et al., Coordinate activation of Shh and PI3K signaling in PTEN-deficient glioblastoma: new therapeutic opportunities. Nat Med, 2013. 19(11): p. 1518–23.
3. Gao, Y., et al., A self-directed Trojanbot-enzymatic nanobot in neutrobot for active target therapy of glioblastoma. Nat Commun, 2025. 16(1): p. 5263.
4. Faust Akl, C., et al., Glioblastoma-instructed astrocytes suppress tumour-specific T cell immunity. Nature, 2025. 643(8070): p. 219–229.
5. Sun, X., et al., High-dose ascorbic acid selectively induces pyroptosis in LKB1-deficient lung cancer and sensitizes immunotherapy. Cell Rep Med, 2025. 6(9): p. 102291.
6. Sarn, N., et al., Cytoplasmic-predominant Pten increases microglial activation and synaptic pruning in a murine model with autism-like phenotype. Mol Psychiatry, 2021. 26(5): p. 1458–1471.
7. Rangel, R., et al., Impaired angiogenesis in aminopeptidase N-null mice. Proc Natl Acad Sci U S A, 2007. 104(11): p. 4588–93.
8. Zhang, Z.Q., et al., Rapid endothelialization of printed vascular grafts by perivascular niche-circulating endothelial progenitors crosstalk. Nat Commun, 2025. 17(1): p. 1311.
9. Yang, Y., et al., Accumulated BCAAs and BCKAs contribute to the HFD-induced deterioration of Alzheimer's disease via a

dysfunctional TREM2-related reduction in microglial beta-amyloid clearance. J Neuroinflammation, 2024. 21(1): p. 327.
10. Zhang, H., et al., Glycine-Histidine-Lysine (GHK) Alleviates Astrocytes Injury of Intracerebral Hemorrhage via the Akt/miR-146a-3p/AQP4 Pathway. Front Neurosci, 2020. 14: p. 576389.
11. Gao, Y., et al., The emergence of Sox and POU transcription factors predates the origins of animal stem cells. Nat Commun, 2024. 15(1): p. 9868.
12. Jin, H., et al., Genetic fate-mapping reveals surface accumulation but not deep organ invasion of pleural and peritoneal cavity macrophages following injury. Nat Commun, 2021. 12(1): p. 2863.

Eukaryotic cell lines

Policy information about cell lines and Sex and Gender in Research

Cell line source(s)	The six primary cell cultures used (U3013MG, U3028MG, U3054MG, U3179MG, U3180MG and U3220MG) are patient-derived primary glioblastoma cultures obtained from the Human Glioblastoma Cell Culture (HGCC) biobank, Uppsala University (https://hgcc.se), where they are identified by these HGCC accession names; they are not commercial cell lines and have no vendor catalogue number. The donor sex of each culture (U3013MG, female; U3028MG, female; U3054MG, female; U3220MG, female; U3179MG, male; U3180MG, male), per-culture clinical and genomic annotations are given in Supplementary Data, sheet 2. All cultures were confirmed mycoplasma-negative (MycoAlert, Lonza #LT07-418) and were authenticated by the HGCC biobank.
Authentication	Cell lines were authenticated by STR profiling prior to use in experiments by the HGCC biobank.
Mycoplasma contamination	All cultures were confirmed for Mycoplasma negative by mycoAlert Mycoplasma Detection Kit from Lonza, caralog #LT07-318.
Commonly misidentified lines (See ICLAC register)	None of the cell lines used to appear on the ICLAC register of commonly misidentified cell lines.

Animals and other research organisms

Policy information about studies involving animals; ARRIVE guidelines recommended for reporting animal research, and Sex and Gender in Research

Laboratory animals	Athymic nude mice (Hsd: Athymic Nude-Foxn1nu, genetic background: Foxn1nu/Foxn1nu, source: Envigo, Netherlands), house 5 mice per cage, 12h light/dark at ambient temperature and humidity. The mice had unrestricted access to food and water.
Wild animals	No wild animals were used in this study.
Reporting on sex	Only female nude mice were used for all the experiments and to permit group housing and to reduce inter animal variability. The host sex was therefore not the study variable in this work.
Field-collected samples	No any field-collected samples were used in this study.
Ethics oversight	All animal experiments were conducted with an ethical permit granted by the Uppsala Animal Research Ethical Board, Sweden, permits 5.8.18-06726/2020 and 5.8.18-00216/2023, for all animal work; and the Uppsala Regional Ethical Review Board, Sweden, approval 2007/353, for the collection of human tumour material for the HGCC biobank.

Note that full information on the approval of the study protocol must also be provided in the manuscript.

Clinical data

Policy information about clinical studies

All manuscripts should comply with the ICMJE guidelines for publication of clinical research and a completed CONSORT checklist must be included with all submissions.

Clinical trial registration	*Provide the trial registration number from ClinicalTrials.gov or an equivalent agency.*
Study protocol	*Note where the full trial protocol can be accessed OR if not available, explain why.*
Data collection	*Describe the settings and locales of data collection, noting the time periods of recruitment and data collection.*
Outcomes	*Describe how you pre-defined primary and secondary outcome measures and how you assessed these measures.*

Plants

Seed stocks

Report on the source of all seed stocks or other plant material used. If applicable, state the seed stock centre and catalogue number. If plant specimens were collected from the field, describe the collection location, date and sampling procedures.

Novel plant genotypes

Describe the methods by which all novel plant genotypes were produced. This includes those generated by transgenic approaches, gene editing, chemical/radiation-based mutagenesis and hybridization. For transgenic lines, describe the transformation method, the number of independent lines analyzed and the generation upon which experiments were performed. For gene-edited lines, describe the editor used, the endogenous sequence targeted for editing, the targeting guide RNA sequence (if applicable) and how the editor was applied.

Authentication

Describe any authentication procedures for each seed stock used or novel genotype generated. Describe any experiments used to assess the effect of a mutation and, where applicable, how potential secondary effects (e.g. second site T-DNA insertions, mosiacism, off-target gene editing) were examined.
