# Supplementary material for: Reconstructing the single-cell spatiotemporal dynamics of glioblastoma invasion
Source: Nat Commun. 2026 Nov 2;17:11245. doi: 10.1038/s41467-026-78908-9 (PMC13728213; doi:10.1038/s41467-026-78908-9)
Supplement: Supplementary file 2 [file 41467_2026_78908_MOESM13_ESM.pdf]

nature portfolio

Peer Review File

Reconstructing the single-cell spatiotemporal dynamics of glioblastoma invasion

Corresponding Author: Professor Sven Nelander

This file contains all reviewer reports in order by version, followed by all author rebuttals in order by version.

Version 0:

Reviewer comments:

Reviewer #1

(Remarks to the Author)
This manuscript by Mangukiya and Skeppas et al presents a novel method called Gliotrace for measuring the invasion dynamics of glioblastoma PDX slices. This an important area of study given that mechanisms of glioma invasion are still poorly understood. They use an AI framework for interpreting tumour invasion from imaging protocols (confocal microscopy), and by doing this were able to detect different types of tumour invasion such as vessel guided migration and immune cell interactions. The data are clearly presented and the paper is well written. However, I have some concerns that should be addressed prior to publication:
1. It is difficult to appreciate the molecular subtypes of glioma that are being modelled in the absence of molecular data. Can the authors measure expression of oncogenes or other key markers (eg IDH, EGFR, PDGFRA, MDM2, PTEN etc), or ideally perform RNA-seq on their samples? Citation of the relevant literature consistent with the subtypes being modelled is also required. This will help interpretation of relevance of their findings to the appropriate glioma subtypes.
2. Lines 111-113 - it is too speculative in my opinion to state in the Results section that this method can be directly applied to high-throughput drug screens in the absence of such data.
3. The authors show that Gliotrace provides a means to analyse microglia dynamics at the GBM border. This is interesting. However, how does this fit with recent and prior literature showing immunosuppressive TREM2+ microglia at border increasing GBM growth eg PMID: 37324244? Their findings need to be discussed in this context.
4. The authors observed that dasatinib-treated U3054MG cells showed a reduction in locomoting-to diffuse translocation transitions. In order to improve the clinical relevance, can the authors show if this improves survival in vivo or in patients' clinical data, ideally through experiment but if not through correlation with any published datasets?
5. They find spatial separation between proliferative and invasive tumour dynamics. Could this be a link with oncogene specific dynamics eg CDK4 vs EGFR - this can be discussed, as other works do identify differences based on the oncogene or transcriptional phenotypes.

(Remarks on code availability)

Reviewer #2

(Remarks to the Author)
Manuscript background information
The manuscript introduces GlioTrace, a high-throughput and scalable framework for analyzing glioblastoma invasion dynamics in patient-derived xenograft (PDCX) brain slices using live imaging and AI-driven analytics. The use of the full field of view in brain slices and the integration of convolutional neural networks (CNNs) and Hidden Markov Models (HMMs) to model phenotypic transitions in migrating tumor cells represents a strong methodological innovation. The manuscript is well-organized and flows smoothly from introduction through methods and results. Figure legends, in-text references, and method descriptions are clearly written and well-explained. The analytical framework presented in this work can serve as a useful tool for other investigators studying migration and invasion, not only in glioma but also in other tumors.

Major Revisions:
1. The migration patterns described in this study, “diffuse invasion” and “perivascular invasion”, as well as the migratory

phenotypes, including branching, translocating, and locomotive cells, are not entirely novel. The evaluation of diffuse and perivascular invasion has been extensively studied. Moreover, the described morpho-dynamics have already been reported in intravital imaging studies, brain slices, and other live-cell microscopy platforms (e.g., Schubert et al., 2024; Venkataramani et al., 2022; Comba et al., 2022; Griveau et al., 2018, Ralitza Staneva et al, 2019, Maria Alieva et al, 2019; and publications from Peter Friedl's research group). While the authors acknowledge these precedents, it should be made more explicit that GlioTrace's main contribution lies in its scalability and computational modeling of these known behaviors, rather than in the discovery of new invasion phenotypes.
a) How do these migration patterns correlate with previously described collective and single-cell migration behaviors?
b) Can this platform be used to revisit and analyze prior datasets to identify similarities and thus validate the robustness of the proposed data analysis?
c) Is there any spatial correlation or distance-specific organization in the movement of branching, translocating, and locomotive cells?

2. The study uses six well-characterized PDCX models spanning major transcriptional subtypes (proneural, mesenchymal, classical), which supports the broader applicability of findings. However, conclusions regarding subtype-specific invasion patterns are not clearly supported, and six tumors may be insufficient to draw robust conclusions regarding associations between subtype and invasion behavior. Since the authors claim that the platform enables classification of invasive phenotypes and quantification of transitions to define patient-specific invasion patterns, it would be important to incorporate data or discussion on how the proposed glioma migration strategies correlate with glioma malignancy at biological, histopathological, and molecular levels.
a) Incorporate patient clinical data (e.g., survival, recurrence status) or parallel mouse survival experiments to contextualize migration patterns biologically.
b) Describe any molecular mechanism (e.g., expression of migration-related genes or pathways) associated with different invasion modes.
c) Consider integrating molecular data to define whether specific migratory phenotypes (e.g., branching cells) correlate with particular gene expression programs.
d) Are branching cells more aggressive or treatment-resistant than other types? Quantitative analysis of survival, proliferation, or drug response would add important functional insight.
e) What are the morphological and histological features of each migration pattern and cell phenotype? Can dynamic behaviors be linked with histological characteristics?
f) Consider discussing how this platform could support functional stratification of patients for clinical trial inclusion.

3. This platform has significant translational potential for precision oncology by enabling the quantification of patient-specific invasion phenotypes and drug response profiles. The study includes both untreated and drug-treated conditions (dasatinib and thapsigargin) and evaluates multiple invasion-related metrics (morphology, movement, microenvironment interactions, and phenotypic plasticity). However:
a) The rationale for selecting these two drugs should be clarified. How do their known molecular targets relate to glioma invasion pathways? While dasatinib and thapsigargin have been previously identified as anti-invasive agents, briefly linking their molecular targets to glioma invasion pathways would strengthen the justification for their use in this study.
b) Importantly, there is no evaluation of standard-of-care therapies (radiation and temozolomide). Testing these agents, or their combinations, would improve translational relevance, especially in assessing treatment-resistant invasion phenotypes. Are the patient-derived glioma cells characterized in terms of MGMT promoter methylation status, radio-sensitivity or resistance, and whether they originate from primary or recurrent tumors?

4. The methods section is well-documented and reproducible, with clear reporting of software versions and imaging parameters. However, some clarifications are needed:
a) Define the time-lapse imaging parameters more precisely. It is currently stated as "no more than 2 hours per frame." What was the exact interval used?
b) How many movies were acquired per PDCX model?
c) Describe the number of biological replicates (i.e., independent mouse brains per model). Imaging many ROIs per slice is valuable, but independent biological replicates are essential to support conclusions.
d) What criteria were used to determine “optimal tumor growth” (line 420) as the starting point for imaging?

5. In lines 129–130, the authors state that U3220MG shows meningeal growth and U3054MG perivascular growth. However, based on the images in Figure 1E and F, both appear to show meningeal invasion. Please clarify.

6. Proliferation and cell death status for each PDCX model should be demonstrated at the imaging endpoint.

7. In lines 188–191, the authors describe that U3180MG cells alternate between vessel-associated and non-vessel-associated migration, indicating an "inherent ability to switch" between modes. What intrinsic features of the cells support this adaptability? Is it driven by cell-intrinsic properties or the tumor microenvironment?

8. The interaction between glioma cells and blood vessels is well demonstrated. Are tumor cells physically interacting with perivascular pericytes or endothelial cells? What molecular features influence whether cells migrate via perivascular routes?

9. Regarding glioma–microglia/macrophage interactions: What is the role of microglia in determining glioma migration patterns? Could genetic or chemical inhibition of microglia alter glioma invasion behavior?

10. Cell segmentation is limited in the tumor core. Including a nuclear marker or dye would improve segmentation and

tracking in dense tumor areas.

Minor Revisions
• The manuscript is dense in some areas, particularly the Results section. Breaking up long paragraphs and improving transitions between topics (e.g., movement dynamics → phenotype classification → HMM modeling) would enhance readability.
• Some figure panels (e.g., Figures 2L–M, 4F) should be more clearly described in the main text to help readers unfamiliar with computational modeling better interpret them.

(Remarks on code availability)

Reviewer #3

(Remarks to the Author)
The authors developed GlioTrace to analyze 10x whole slide time lapse videos of PDCX specimens to analyze GBM invasion dynamics. Applying GlioTrace on PDCX specimens from 6 GBM patients, the authors identified three cell phenotypes (branching, trnaslocating, and locomotive). The authors observed different migration and phenotype transition patterns in different patients, and how drug treatment affect these patterns. GlioTrace can potentially analyze GBM invasion phenotypes and their modulation by therapy, and enabling functional profiling for precision oncology.

Questions for authors:

1. The use of a HMM to denoise CNN-predicted morphological classes raises concerns. In a standard HMM, the hidden state is meant to represent an unobserved biological process that causally generates the observed state. Here, hidden and observed states are defined over the same discrete classes, effectively using the HMM to correct CNN classification errors rather than uncovering latent dynamics, especially by setting emission probability to the CNN evaluation confusion matrix. However, CNN inference errors arise from model limitations, and do not represent meaningful causal relationships between cell states. This raises questions about the appropriateness of the HMM modeling framework in this context. Can the authors clarify the use of HMM? Does simpler approaches, such as direct counting, yield comparable insights? Have ablation studies been conducted to assess whether the HMM provides value beyond temporal smoothing of CNN outputs?

2. The description of the CNN used for cell state classification lacks sufficient detail, raising concerns about the scalability and generalizability of GlioTrace. The model architecture, training hyperparameters and configurations are not specified.How are the training, validation, and test sets constructed, and are they stratified by patient? Importantly, details regarding data processing are also missing. Cells are inherently elliptical or irregular in shape, and may overlap with neighboring cells. How are the images cropped and preprocessed for CNN input? Would the authors provide representative input images and class annotations, and how does surrounding context affect classification performance?

3. The cell detection pipeline in GlioTrace appears to rely on classical image processing techniques. Can the authors report quantitative performance metrics for detection accuracy, such as mean average precision (mAP), benchmarked against manual annotations? How do detection errors impact downstream tracking, CNN classification, and HMM modeling? Have the authors considered benchmarking or replacing this step with a learned segmentation approach?

4. GlioTrace is only validated on 6 PDCX models. This raises concerns regarding the scalability of the proposed model, as well as the generalizibility of the observations reported in the paper. Critical details regrading training / validation set split should be discussed further. Have the authors tested the model in separate, held-out PDCX lines to evaluate generalization beyond the training cohort?

5. The analysis of GlioTrace seems to be focus on tumor cells and blood vessels, but doesn't model other normal cells at the margin or immune cells. How does this limited representation of the tumor microenvironment affect the generalizability of the findings, especially given the known importance of neurons, glial cells and immune cells in GBM infiltration?

6. How would GiloTrace be integrated into the clinical workflow for personalized treatment recommendation, and how does it compare to the current standard-of-care?

7. All experimentation are performed using PDCX models. How reliable are these models to reflect how tumors behave in human? his raises concerns about the translational applicability of GlioTrace for patient-specific treatment guidance.

(Remarks on code availability)
The code base is generally well written, with many functions clearly documented. However, it would benefit from improved organization and the inclusion of a README file with instructions for running the code. It is somewhat difficult to locate specific components of GlioTrace, such as the CNN training and inference pipelines. Some functions, including "customReadFunction_semseg.m", "customReadFunction.m", "customReadFunction3.m", "customReadFunction5.m", are repetitive and lack documentation, causing confusion. Additionally, while the use of MATLAB is understandable for the project, much of the machine learning community has transitioned to Python. Relying on a proprietary language like MATLAB may pose barriers to wider adoption and long-term maintenance of the tool.

Reviewer #4

(Remarks to the Author)
This study introduces GlioTrace, an ex vivo imaging and AI-based analytical framework for tracking glioblastoma invasion dynamics in patient-derived xenograft (PDCX) brain slices. The authors combine confocal microscopy with computational modeling using convolutional neural networks and Hidden Markov Models to identify distinct invasion modes and quantify patient-specific variations in invasion plasticity. Using six PDCX models, they demonstrate different invasion patterns (bulky/perivascular vs. diffuse), characterize morphological transitions between branching, translocating, and locomotive cell types, and show that targeted therapies can selectively modulate invasion phenotypes with spatially and temporally distinct drug responses.
Overall, I find this work interesting in its approach to analyzing glioblastoma invasion dynamics, but I am unsure about the quality of the model system and whether it adequately recapitulates in vivo conditions.
Major Points
1. The model system itself has made an interesting modification to previous approaches. While the authors now use organotypic brain slices from previously engrafted tissue, previous work has used organotypic slices with subsequent injection of cancer cells. The fundamental limitations of slice culture systems should be addressed, and the improvement over existing methods should be clearly demonstrated. The authors should directly compare their approach to existing slice culture methods and intravital microscopy to demonstrate clear advantages and validate that their observations reflect genuine biological processes rather than culture artifacts. Specifically, the authors should validate that their observed patient-specific invasion patterns are comparable to in vivo behavior, potentially using two-photon microscopy (e.g. as in Griveau et al., Cancer Cell 2018). This is particularly important because strict perivascular invasion patterns can also be observed in unhealthy organotypic brain slices as an artifact rather than a true biological phenomenon.
2. The scalable approach is interesting, but it remains unclear how tumor cells and their microenvironment are affected by slice culture conditions. Supplementary Figure 1D shows that approximately 30% of cells are dead (EthD-1 positive), which raises serious concerns about tissue health. Previous work has established that adult neurons cannot be properly maintained in slice cultures, which is why organotypic hippocampal slice cultures typically use embryonic tissue. The fragmented appearance of vasculature in some images further suggests compromised tissue integrity. The authors should provide comprehensive characterization of the health and function of all microenvironmental components over time, including vascular cells, neurons, glial compartments, and immune cells.
3. While the drug responses are interesting, it is unclear whether these effects are mediated through the microenvironment or represent direct effects on cancer cells. The authors should distinguish between these mechanisms and demonstrate that observed responses are physiologically relevant rather than artifacts of the slice culture system.
4. The number of xenografted mice used for each experiment is unclear, as mostly slice and cell numbers are reported. The authors should provide exact numbers of mice used and compare preparation heterogeneity between mice to ensure that observed patient-specific differences reflect genuine biological variation rather than preparation artifacts.

Minor Points
1. How are "core" and "periphery" regions exactly defined in this study? Clear criteria should be provided.
2. How are "junk" cells defined, and can the authors provide more examples? Are these cells alive and functional, or are they artifacts of the slice culture system? This distinction is important for interpreting the biological relevance of the findings.

(Remarks on code availability)

Version 1:

Reviewer comments:

Reviewer #1

(Remarks to the Author)
The authors have addressed my comments. Congratulations to the authors on this interesting and informative study.

(Remarks on code availability)
n/a

Reviewer #2

(Remarks to the Author)
The authors are commended for the thorough revisions to the manuscript. While Reviewer #2's concerns have been largely addressed, several aspects would benefit from further strengthening. These represent relatively minor revisions that would enhance the overall quality of the study and increase the utility of GlioTrace as a tool for the broader scientific community.

1) Given that the central contribution of this work is the development of GlioTrace as a scalable analytical tool, it is essential to ensure that the software and methodology are fully accessible and user-friendly for the scientific community. Improving the GitHub repository, with clear organization, comprehensive documentation, step-by-step tutorials, and example datasets, will greatly enhance reproducibility and adoption. In addition, the development of a more user-friendly interface or packaged

software for analysis would further increase its impact and usability across a broader range of researchers.

2) While it is important to clearly describe how the glioma models were generated and analyzed, particularly in the context of endogenous tumor growth within intact brain tissue. The use of orthotopic or genetically engineered tumor models combined with tissue slice analysis is not, by itself, novel. Therefore, the authors should clarify how their approach advances beyond existing methodologies. Please revise the statement in lines 86–89 and expand the Discussion accordingly to better position the innovation of the model.

3) Several points addressed in the rebuttal letter should be explicitly incorporated into the manuscript, rather than remaining only in the responses. For example, the discussion of similarities with previously described migration modes (Response 1a and 1c) and the rationale for drug selection (Response 3a) should be clearly integrated into the main text to improve transparency and context.

4) The authors state that GlioTrace-derived migration phenotypes correspond to well-established histopathological invasion patterns. To strengthen this claim, it would be highly valuable to include representative H&E histological images alongside the immunofluorescence data. This would facilitate interpretation and provide a clearer link between dynamic behaviors and established pathological features, supporting statements such as the correspondence between perivascular translocation and vessel co-option, or diffuse migration and parenchymal infiltration.

5) Moreover, clearer acknowledgment of limitations related to sample size and the lack of direct clinical correlation would improve the robustness and balance of the conclusions.

***In addition, since Reviewer #4 who assessed the previous version of this manuscript is unavailable for this round of peer review, Dr. Lizhao Wu has asked me to provide my feedback on whether authors have adequately addressed Reviewer #4's previous concerns. Here is my response in brackets following Reviewer #4's comments:

This study introduces GlioTrace, an ex vivo imaging and AI-based analytical framework for tracking glioblastoma invasion dynamics in patient-derived xenograft (PDCX) brain slices. The authors combine confocal microscopy with computational modeling using convolutional neural networks and Hidden Markov Models to identify distinct invasion modes and quantify patient-specific variations in invasion plasticity. Using six PDCX models, they demonstrate different invasion patterns (bulky/perivascular vs. diffuse), characterize morphological transitions between branching, translocating, and locomotive cell types, and show that targeted therapies can selectively modulate invasion phenotypes with spatially and temporally distinct drug responses. Overall, I find this work interesting in its approach to analyzing glioblastoma invasion dynamics, but I am unsure about the quality of the model system and whether it adequately recapitulates in vivo conditions.

Major Points
1. The model system itself has made an interesting modification to previous approaches. While the authors now use organotypic brain slices from previously engrafted tissue, previous work has used organotypic slices with subsequent injection of cancer cells. The fundamental limitations of slice culture systems should be addressed, and the improvement over existing methods should be clearly demonstrated. The authors should directly compare their approach to existing slice culture methods and intravital microscopy to demonstrate clear advantages and validate that their observations reflect genuine biological processes rather than culture artifacts. Specifically, the authors should validate that their observed patient-specific invasion patterns are comparable to in vivo behavior, potentially using two-photon microscopy (e.g. as in Griveau et al., Cancer Cell 2018). This is particularly important because strict perivascular invasion patterns can also be observed in unhealthy organotypic brain slices as an artifact rather than a true biological phenomenon.
2. The scalable approach is interesting, but it remains unclear how tumor cells and their microenvironment are affected by slice culture conditions. Supplementary Figure 1D shows that approximately 30% of cells are dead (EthD-1 positive), which raises serious concerns about tissue health. Previous work has established that adult neurons cannot be properly maintained in slice cultures, which is why organotypic hippocampal slice cultures typically use embryonic tissue. The fragmented appearance of vasculature in some images further suggests compromised tissue integrity. The authors should provide comprehensive characterization of the health and function of all microenvironmental components over time, including vascular cells, neurons, glial compartments, and immune cells.

[
Regarding points 1 and 2, I believe the authors have substantially addressed the reviewer's concerns, and I do not consider two-photon microscopy to be essential for the scope of this manuscript. The primary objective of the study is to establish and characterize an organotypic glioma slice platform rather than to replicate all aspects of in vivo imaging approaches. Moreover, two-photon microscopy can be performed for a few hours and is limited to the cranial window.
That said, the manuscript would be significantly strengthened by additional histopathological validation of tissue integrity and microenvironmental preservation during culture. Specifically, I recommend including H&E staining and immunohistochemical analyses for markers of key microenvironmental components, such as CD31 and SMA (vasculature), Nestin and SOX9 (glioma cells), and CD3 and CD45 (immune populations) that have not yet been included. These analyses should compare slices at baseline (day 0) and at the experimental endpoint (day 6), alongside FFPE coronal sections obtained directly from the corresponding in vivo tumors.
Such experiments would help determine whether the organotypic slices maintain the cellular architecture and composition of the parental tissue over time and would address concerns regarding potential culture-induced artifacts. In addition, these studies would directly complement the reviewer's concern regarding the approximately 30% EthD-1–positive cells reported in Supplementary Figure 1D. Incorporating multiplex IF for proliferation and apoptosis markers, such as Ki67 and cleaved

Caspase-3, and the markers mentioned above would further define whether cell death or compensatory proliferation occurs during culture and identify the specific cellular compartments affected.
Overall, these analyses would provide a more comprehensive assessment of tumor tissue viability and microenvironmental stability, thereby strengthening confidence that the observed invasion patterns reflect biologically relevant processes rather than artifacts of ex vivo culture.
]

3. While the drug responses are interesting, it is unclear whether these effects are mediated through the microenvironment or represent direct effects on cancer cells. The authors should distinguish between these mechanisms and demonstrate that observed responses are physiologically relevant rather than artifacts of the slice culture system.

[
The authors have addressed this concern by performing additional analyses, including covariate-dependent transition modeling to evaluate migration behavior across different microenvironmental niches and single-cell transcriptomic profiling of treated slices. These analyses support the conclusion that microenvironmental factors do not solely drive the observed drug responses. However, additional functional validation would further strengthen this interpretation. For example, migration assays could be performed in explant slices derived from transgenic models with microglial depletion or dysfunction, or by comparing responses in immunocompetent versus immunodeficient hosts. Given the well-established role of microglia as key regulators of glioma invasion, such experiments would help distinguish tumor cell–intrinsic effects from microenvironment-mediated mechanisms and further validate the physiological relevance of the findings.
]

4. The number of xenografted mice used for each experiment is unclear, as mostly slice and cell numbers are reported. The authors should provide exact numbers of mice used and compare preparation heterogeneity between mice to ensure that observed patient-specific differences reflect genuine biological variation rather than preparation artifacts.
[
Reviewer concerns were addressed; no further comments.
]

Minor Points
1. How are "core" and "periphery" regions exactly defined in this study? Clear criteria should be provided.
[
The criteria used were provided in the new manuscript version.
]

2. How are "junk" cells defined, and can the authors provide more examples? Are these cells alive and functional, or are they artifacts of the slice culture system? This distinction is important for interpreting the biological relevance of the findings.
[
The authors clarified that the original “junk” cell category is in the new manuscript version. However, if feasible, it would be valuable to characterize these regions further using live/dead staining or apoptosis markers. Such analyses would help determine whether these cells represent viable but densely packed tumor cells, stressed cells, or non-viable cellular material, thereby strengthening the biological interpretation of this classification and excluding potential artifacts related to tissue preparation or culture conditions.
]

(Remarks on code availability)

Reviewer #3

(Remarks to the Author)
I would like to thank the authors for a detailed response, and my questions were sufficiently addressed. I have no further remarks or suggestions.

***In addition, since Reviewer #4 who assessed the previous version of this manuscript is unavailable for this round of peer review, Dr. Lizhao Wu has asked me to provide my feedback on whether authors have adequately addressed Reviewer #4's previous concerns. Here is my response:

My expertise is primarily in computational methods and machine learning, rather than the biological aspects or field-specific norms. With that caveat, based on my reading, the authors’ responses appear appropriate and seem to address the major concerns raised by Reviewer #4. I do not have additional major concerns.

(Remarks on code availability)

The authors have made an excellent effort to improve the source code. The updated repository contains detailed documentation and comments throughout the code, along with a notebook to demonstrate usage. Please make sure the MATLAB version of the code remain accessible, as I wasn't able to find a link to it.

Version 2:

Reviewer comments:

Reviewer #2

(Remarks to the Author)
The authors have addressed my comments, and I have no further concerns.

(Remarks on code availability)

Reviewer #3

(Remarks to the Author)
I thank the authors for the response. They have addressed my concerns.

(Remarks on code availability)

Open Access This Peer Review File is licensed under a Creative Commons Attribution 4.0 International License, which permits use, sharing, adaptation, distribution and reproduction in any medium or format, as long as you give appropriate credit to the original author(s) and the source, provide a link to the Creative Commons license, and indicate if changes were made.
In cases where reviewers are anonymous, credit should be given to 'Anonymous Referee' and the source.
The images or other third party material in this Peer Review File are included in the article's Creative Commons license, unless indicated otherwise in a credit line to the material. If material is not included in the article's Creative Commons license and your intended use is not permitted by statutory regulation or exceeds the permitted use, you will need to obtain permission directly from the copyright holder.
To view a copy of this license, visit https://creativecommons.org/licenses/by/4.0/

REVIEWER COMMENTS

Reviewer #1 (Remarks to the Author): GBM evolution, GBM mouse models

This manuscript by Mangukiya and Skeppas et al presents a novel method called Gliotrace for measuring the invasion dynamics of glioblastoma PDX slices. This an important area of study given that mechanisms of glioma invasion are still poorly understood. They use an AI framework for interpreting tumour invasion from imaging protocols (confocal microscopy), and by doing this were able to detect different types of tumour invasion such as vessel guided migration and immune cell interactions. The data are clearly presented and the paper is well written. However, I have some concerns that should be addressed prior to publication:

1. *It is difficult to appreciate the molecular subtypes of glioma that are being modelled in the absence of molecular data. Can the authors measure expression of oncogenes or other key markers (eg IDH, EGFR, PDGFRA, MDM2, PTEN etc), or ideally perform RNA-seq on their samples? Citation of the relevant literature consistent with the subtypes being modelled is also required. This will help interpretation of relevance of their findings to the appropriate glioma subtypes.*

Response. Thank you for this suggestion. We agree that molecular context is important for interpreting invasion phenotypes, and we have added a summary of genomic alterations and transcriptional annotations for all six HGCC models to Supplementary Data File, Excel Sheet "HGCC Clinical Data", based on prior profiling of the HGCC collection (Johansson et al., Cell Reports 2020; Doroszko et al., Nature Communications 2025). These annotations confirm that the models retain canonical glioblastoma driver alterations, including EGFR and PDGFRA amplification, CDK4 amplification, CDKN2A loss, and PTEN and TP53 disruption, at frequencies comparable to primary glioblastoma (TCGA 2008; Brennan et al., Cell 2013). All cases are IDH wild type. We note, however, that our models were selected based on invasion phenotype rather than molecular subtype, and we do not consider subtype identity to be the most informative framework for interpreting the dynamics observed here. While bulk tumor subtype classifications correlate with underlying driver alterations (Verhaak et al., 2010; Wang et al., Cancer Cell 2017), recent single-cell studies have shown that individual glioblastoma tumors contain multiple coexisting transcriptional states, NPC-like, OPC-like, AC-like, and MES-like, and that oncogenic alterations bias the distribution of these states rather than defining fixed subtype identities (Neftel et al., Cell 2019; Verhaak et al., Cancer Cell 2010).

In line with this framework, our complementary study of overlapping HGCC models demonstrated that invasion patterns associate with cell-state composition: diffuse invasion correlates with enrichment of NPC-like and astrocyte-like programs, whereas perivascular invasion associates with OPC-like and mesenchymal-like states (Doroszko et al., Nature Communications 2025). Oncogenic alterations may therefore influence invasion behavior indirectly by shaping the underlying cell-state distribution, while the proximate determinant of invasion phenotype is the cell-state landscape. This conceptual framework is now more explicitly described in the revised Discussion, and the added genomic annotations enable readers to relate driver alterations, state composition, and invasion behavior across the six models.

2. *Lines 111-113 - it is too speculative in my opinion to state in the Results section that this method can be directly applied to high-throughput drug screens in the absence of such data.*

Response. Agreed. We have moderated the language accordingly. GlioTrace is now positioned as a scalable tool for quantifying how therapeutic perturbations alter invasion dynamics, including movement, morphological state transitions, and microenvironmental interactions, rather than as a high-throughput screening platform.

3. *The authors show that Gliotrace provides a means to analyse microglia dynamics at the GBM border. This is interesting. However, how does this fit with recent and prior literature showing immunosuppressive TREM2+ microglia at border increasing GBM growth eg PMID: 37324244? Their findings need to be discussed in this context.*

Response: Thank you for this comment. We stained for TREM2 and observed that TREM2^{+} microglia are predominantly localized to the tumor core in our nude mouse PDX brain slice model **(Supplementary Fig. 9).** This contrasts with reports in human GBM, where TREM2 expression at the invasive edge is linked to CD8^{+} T cell and PD-L1 co-localization, indicating region-specific immunophenotypes driven by adaptive immune activity (Noorani, 2023, PMID: 37324244). High TREM2 expression in GBM patient samples is predominantly myeloid-associated and correlates with poorer survival (PMID: 37172094). The discrepancy between our model and human GBM is most likely explained by immune context. While our model has macrophage/microglia populations and B cells **(Fig. 2),** Nude mice lack functional CD8^{+} T cells, which are key drivers of TREM2^{+} microglial recruitment and stabilization at the invasive edge in immunocompetent settings. In their absence, TREM2 expression is likely driven by tumor-intrinsic signals including hypoxia, lipid debris, and phagocytic demand, that confine TREM2^{+} microglia to the tumor core. This is now discussed explicitly in the limitations section of the revised manuscript, where we note that the absence of adaptive immunity limits the generalizability of our microglial findings to immunocompetent settings.

4. *The authors observed that dasatinib-treated U3054MG cells showed a reduction in locomoting-to diffuse translocation transitions. In order to improve the clinical relevance, can the authors show if this improves survival in vivo or in patients' clinical data, ideally through experiment but if not through correlation with any published datasets?*

Response. Thank you for this clinically relevant question. The organotypic brain slice system used here is designed to measure single-cell invasion dynamics and does not capture survival outcomes. To provide clinical context, we have added a brief note on prior clinical experience with dasatinib in glioblastoma (Results, p.14). Although SRC inhibition suppresses invasion in preclinical models, clinical trials have not demonstrated improvements in overall survival, likely reflecting limited brain penetration and heterogeneous pathway dependence (Reardon et al., 2012; Galanis et al., 2019). Our goal in using dasatinib was therefore not to assess survival, but to determine whether GlioTrace can resolve drug-induced changes in invasion phenotypes at single-cell resolution. Consistent with its known effects on cytoskeletal and adhesion signaling, dasatinib reduced cell motility (**Supplementary Fig. 13),** shifted the population toward low-motility round states, and altered transitions between invasion phenotypes, including increased transitions into non-motile states **(Fig. 6).** Our interest in dasatinib as a modulator of invasion plasticity is further supported by recent work showing effects on glioblastoma cell-state composition and therapeutic sensitivity (Larsson et al., Nat Commun 2024). These additions

clarify the clinical context while emphasizing that GlioTrace provides mechanistic insight into invasion dynamics that complements, rather than replaces, survival-based endpoints.

5. *They find spatial separation between proliferative and invasive tumour dynamics. Could this be a link with oncogene specific dynamics eg CDK4 vs EGFR - this can be discussed, as other works do identify differences based on the oncogene or transcriptional phenotypes.*

Response. Thank you for this insightful comment. We have expanded the Discussion to address the relationship between oncogenic alterations and invasion dynamics, and we now provide genomic annotations for all models (Supplementary Data File, Excel sheet "HGCC clinical data") to support this analysis. The spatial separation between proliferative and invasive dynamics that we observe is consistent with the known relationship between oncogenic alterations, transcriptional cell-state composition, and tumor organization. Single-cell studies have shown that key driver alterations bias the distribution of coexisting glioblastoma cell states: for example, CDK4 amplification and CDKN2A loss are associated with proliferative progenitor-like states, whereas EGFR and PDGFRA activation are linked to glial-lineage programs with invasive potential (Neftel et al., 2019; Verhaak et al., 2010; Cancer Genome Atlas Research Network, 2008; Brennan et al., 2013). These states have been reported to exhibit spatial biases within tumors, with proliferative populations tending to localize to tumor cores, while OPC-like and mesenchymal-like programs are more frequently associated with invasive and perivascular regions.

Importantly, oncogenic alterations bias this landscape without determining it. Multiple transcriptional states coexist within individual tumors, and invasion behavior emerges from the composition and plasticity of the cell-state landscape rather than from any single oncogenic event (Doroszko et al., 2024). In the revised manuscript, we therefore frame spatial compartmentalization of proliferative and invasive dynamics in terms of transcriptional state organization, while avoiding direct oncogene-to-phenotype assignments that cannot be resolved in the present experimental system.

Reviewer #2 (Remarks to the Author): GBM invasion, spatial

Manuscript background information
The manuscript introduces GlioTrace, a high-throughput and scalable framework for analyzing glioblastoma invasion dynamics in patient-derived xenograft (PDCX) brain slices using live imaging and AI-driven analytics. The use of the full field of view in brain slices and the integration of convolutional neural networks (CNNs) and Hidden Markov Models (HMMs) to model phenotypic transitions in migrating tumor cells represents a strong methodological innovation. The manuscript is well-organized and flows smoothly from introduction through methods and results. Figure legends, in-text references, and method descriptions are clearly written and well-explained. The analytical framework presented in this work can serve as a useful tool for other investigators studying migration and invasion, not only in glioma but also in other tumors.

Response. Thank you for this generous assessment. We are pleased that the methodological innovations are clearly communicated and that the framework is seen as broadly applicable.

Major Revisions:

1. *The migration patterns described in this study, "diffuse invasion" and "perivascular invasion", as well as the migratory phenotypes, including branching, translocating, and locomotive cells, are not entirely novel. The evaluation of diffuse and perivascular invasion has been extensively studied. Moreover, the described morpho-dynamics have already been reported in intravital imaging studies, brain slices, and other live-cell microscopy platforms (e.g., Schubert et al., 2024; Venkataramani et al., 2022; Comba et al., 2022; Griveau et al., 2018, Ralitza Staneva et al, 2019, Maria Alieva et al, 2019; and publications from Peter Friedl's research group). While the authors acknowledge these precedents, it should be made more explicit that GlioTrace's main contribution lies in its scalability and computational modeling of these known behaviors, rather than in the discovery of new invasion phenotypes.*

Response. Thank you for this important comment. We fully agree that the invasion phenotypes described here, including diffuse and perivascular invasion, as well as branching, translocating, and locomotive behaviors, have been documented in prior intravital and live-cell imaging studies. We have revised the Discussion to more explicitly acknowledge this body of work (e.g. Venkataramani et al., 2022; Griveau et al., 2018; Alieva et al., 2019; Comba et al., 2022; Schubert et al., 2024; Staneva et al., 2019; and related studies from Friedl and colleagues). The primary contribution of GlioTrace is therefore not the identification of new invasion phenotypes, but their scalable and computationally formalized characterization across large populations of tumor cells in intact brain tissue. Whereas prior approaches typically rely on manual annotation or analysis of limited numbers of cells within restricted imaging volumes, GlioTrace enables automated classification of thousands of cells, unbiased sampling across anatomical regions, and quantitative inference of phenotype transition dynamics and microenvironmental dependencies using a probabilistic state-space framework. This scale of analysis enables new insights into invasion dynamics, including the identification of branching cells as a highly connected hub state, the characterization of round cells as a largely absorbing state, and the quantification of microenvironmentally modulated transition probabilities. We have clarified this positioning in the revised Discussion and Results, and added a comparison table (Supplementary Data File, Excel Sheet "Comparison Table") summarizing experimental modalities and their analytical scope.

a) *How do these migration patterns correlate with previously described collective and single-cell migration behaviors?*

Response. Thank you for this important question. The invasion phenotypes identified by GlioTrace align closely with established migration typologies. Branching, locomoting, and diffuse translocation behaviors correspond to previously described single-cell invasion modes, including exploratory protrusive activity, directional translocation, and diffuse parenchymal infiltration, observed in neuronal-like and progenitor-like tumor cell populations (Venkataramani et al., 2022). Perivascular translocation corresponds directly to vessel co-option and vascular-guided migration (Griveau et al., 2018). Cells classified as round or low-motility are consistent with less migratory tumor populations, such as those residing in tumor cores or exhibiting reduced invasive activity, although their precise functional identity remains to be determined. Collective invasion behaviors, including oncostream-like structures (Comba et al., 2022) and coordinated multicellular fronts (Alieva et al., 2019), are also observed in our data as stream-like configurations. However, GlioTrace is primarily designed for single-cell tracking and state inference, and does not explicitly model collective dynamics as a separate class of behavior. The key advance of GlioTrace is

therefore not redefining migration modes, but enabling their scalable and quantitative characterization across thousands of cells, and reconstructing their hierarchical organization and transition dynamics in a manner not accessible to prior approaches.

b) Can this platform be used to revisit and analyze prior datasets to identify similarities and thus validate the robustness of the proposed data analysis?

Response. GlioTrace can be applied to time-lapse datasets containing fluorescently labeled cells with sufficient spatial and temporal resolution for tracking. To demonstrate robustness beyond our own recordings, we performed proof-of-principle re-analyses on two published nervous-system slice datasets with similar sparse-cell migration dynamics: cortical projection neuron migration (Hansen and Hippenmeyer, 2024) and cortical interneuron migration (Lepiemme et al., 2021). Across these datasets, GlioTrace reliably recovered coherent cell trajectories despite differences in imaging platforms, labeling strategies, and sampling rates **(Supplementary Video 8)**. Of note, the glioblastoma-trained morphological classifier is not intended to assign biological identity in neuronal datasets; rather, these analyses demonstrate generalizability of the tracking and trajectory analysis components. A tutorial for applying the workflow to external datasets is provided in the repository.

c) Is there any spatial correlation or distance-specific organization in the movement of branching, translocating, and locomotive cells?

Response. Thank you for this insightful question. We performed two complementary analyses to assess spatial organization of invasion phenotypes. First, nearest-neighbor analysis of tracked cells revealed significant homotypic clustering, with cells of the same morphological state occurring in close proximity more often than expected by chance **(Supplementary Fig. 6).** This effect was most pronounced for perivascularly migrating cells, consistent with stream-like migration along vascular structures, and was also detectable for branching cells in U3013MG and U3180MG. Round cells formed localized clusters, suggesting the presence of microenvironmental niches associated with low-motility states. Second, we extended our Hidden Markov Model (HMM) framework to incorporate local contextual features when modeling state transitions. Using this covariate HMM, we evaluated whether local directional polarization of neighboring cells influences transition dynamics. Regions with highly aligned migration trajectories showed increased probabilities of state persistence for branching, locomoting, and diffuse-translocating states, indicating that coordinated local environments can stabilize specific invasion modes. Together, these analyses support that invasion phenotypes are spatially structured and influenced by both anatomical guidance cues and local microenvironmental context.

2. *The study uses six well-characterized PDCX models spanning major transcriptional subtypes (proneural, mesenchymal, classical), which supports the broader applicability of findings. However, conclusions regarding subtype-specific invasion patterns are not clearly supported, and six tumors may be insufficient to draw robust conclusions regarding associations between subtype and invasion behavior. Since the authors claim that the platform enables classification of invasive phenotypes and quantification of transitions to define patient-specific invasion patterns, it would be important to incorporate data or discussion on how the proposed glioma migration strategies correlate with glioma malignancy at biological, histopathological, and molecular levels.*

a) Incorporate patient clinical data (e.g., survival, recurrence status) or parallel mouse survival experiments to contextualize migration patterns biologically.

Response. Thank you for these important comments. We agree that six models are insufficient for statistically powered subtype-level conclusions, and we have revised the manuscript to make this explicit. Our aim is not to define subtype-specific invasion patterns, but to demonstrate that GlioTrace can resolve patient-specific invasion phenotypes and quantify invasion plasticity across independent models. To strengthen biological and clinical context, we have added clinical annotations for all six models, including primary versus recurrent status, MGMT promoter methylation, and key driver alterations (Supplementary Data File, Excel Sheet "HGCC clinical information"). We further provide endpoint proliferation and apoptosis measurements (Ki67 and cleaved caspase-3) for four models at day 0 and day 6 **(Supplementary Fig. 2).** The Discussion has been expanded to clarify that invasion phenotypes are best interpreted in the context of transcriptional cell-state composition and plasticity, rather than fixed molecular subtype categories, and that larger cohorts will be required to link specific invasion dynamics to clinical outcomes such as malignancy or recurrence risk. While direct survival analyses are beyond the scope of the present ex vivo system, a complementary study from our group (Krona et al., in revision) provides detailed histopathological characterization of the same PDCX collection, supporting the biological representativeness of these models within the broader HGCC cohort.

b) Describe any molecular mechanism (e.g., expression of migration-related genes or pathways) associated with different invasion modes.

Response. Thank you for this important question. We have expanded the Results **(Fig. 2)** and discussion to more explicitly address the molecular basis of invasion phenotypes and their relationship to transcriptional cell states. In prior work (Doroszko et al., Nature Communications 2025), we performed single-cell RNA sequencing across overlapping HGCC models and showed that invasion patterns associate with transcriptional state composition: models with prominent perivascular invasion are enriched for mesenchymal-like and OPC-like programs, whereas diffusely invading models show higher representation of astrocyte-like and NPC-like states. This relationship is now more clearly articulated in the revised manuscript. To further explore molecular associations at the single-cell level, we re-analyzed multiplex protein imaging data (Vectra Polaris) for four overlapping models, applying the GlioTrace classifier to assign probabilistic invasion phenotypes to individual cells in situ. Class-associated differences were observed in markers including IFI16 and MEF2C **(Fig. 3; Supplementary Fig. 4)**, supporting that invasion phenotypes correspond to molecularly distinct cell states. In addition, pharmacological perturbation provides functional support: dasatinib selectively reduces transitions into highly motile translocating states, consistent with the role of SRC-family signaling in cytoskeletal remodeling and migration. Together, these transcriptomic, proteomic, and perturbation-based observations support a link between invasion behavior and underlying molecular programs. We note, however, that a comprehensive mechanistic dissection of invasion-state regulation will require dedicated functional studies beyond the scope of the present work.

c) Consider integrating molecular data to define whether specific migratory phenotypes (e.g., branching cells) correlate with particular gene expression programs.

Response. This has now been addressed in the response above. In brief, protein-level analyses reveal class-associated marker differences including IFI16 and MEF2C, and standard-of-care treatment experiments show enrichment of branching cells following radiotherapy combined with temozolomide, consistent with a treatment-tolerant phenotype. These findings are described in

detail in the molecular mechanisms response above and in the branching cell aggressiveness response below.

d) *Are branching cells more aggressive or treatment-resistant than other types? Quantitative analysis of survival, proliferation, or drug response would add important functional insight.*

Response. This is an interesting point. Branching cells exhibit the highest migration speeds in our dataset and occupy a hub position in the inferred transition network, with the highest transition rates into both translocating and locomotive states, indicating that they represent a highly dynamic and motile invasion state. Following treatment with standard-of-care therapy - radiation combined with temozolomide - we observed a consistent increase in the fraction of branching cells in U3013MG **(Fig. 6F)**, while this effect was absent in U3180MG, which has very low baseline branching cell frequency. This enrichment suggests that branching cells may be relatively resistant to cytotoxic therapy or preferentially persist following depletion of other invasion phenotypes, raising the possibility that they represent a treatment-tolerant invasive state that contributes to post-treatment recurrence. This interpretation is reinforced by the network analysis, in which branching cells occupy a hub position with high outgoing transition rates into other invasive states, making them a plausible reservoir of invasion plasticity under treatment pressure. Whether this reflects selection, phenotypic reprogramming, or both remains to be determined. We have added this observation and its implications to the Discussion.

e) *What are the morphological and histological features of each migration pattern and cell phenotype? Can dynamic behaviors be linked with histological characteristics?*

Response. Thank you for this important question. The migration phenotypes identified by GlioTrace correspond closely to well-described histological invasion patterns. Translocating cells aligned with vascular structures represent the dynamic counterpart of vessel co-option and perivascular invasion; locomotive phenotypes correspond to diffuse single-cell infiltration through parenchyma. Consistent with this, models with condensed perivascular growth patterns (e.g., U3013MG) show enrichment of translocating cells in the dynamic analysis, while diffusely infiltrative models (e.g., U3180MG) show higher proportions of locomotive phenotypes. These relationships suggest that macroscopic growth architectures observed in endpoint histology emerge from distinct underlying migration behaviors. Molecular observations further support this link - mesenchymal tumor cells were enriched in perivascular niches in both PDCX models and human GBM samples from the BrainUK cohort (Doroszko et al.), indicating conservation of molecular markers associated with perivascular invasion between experimental and clinical settings.

f) *Consider discussing how this platform could support functional stratification of patients for clinical trial inclusion.*

Response. We agree that this should be discussed. We view GlioTrace primarily as a discovery platform, and the most immediate translational application is systematic characterization of invasion behavior across larger patient-derived model collections to identify reproducible dynamic subgroups not captured by static molecular profiling. Looking further ahead, patient-derived tumor slices prepared at surgery could in principle be subjected to ex vivo GlioTrace profiling to assess invasion dynamics and drug sensitivity in a timeframe compatible with clinical decision-making, providing a functional readout complementary to existing molecular diagnostics. The most realistic

near-term path toward clinical utility likely runs through biomarker discovery, by identifying molecular correlates of dynamic invasion states, it may become possible to infer invasion phenotype composition from standard clinical specimens without requiring live imaging infrastructure. We have expanded the Discussion to articulate this translational roadmap explicitly while emphasizing that each application requires prospective validation in larger cohorts before clinical implementation.

3. *This platform has significant translational potential for precision oncology by enabling the quantification of patient-specific invasion phenotypes and drug response profiles. The study includes both untreated and drug-treated conditions (dasatinib and thapsigargin) and evaluates multiple invasion-related metrics (morphology, movement, microenvironment interactions, and phenotypic plasticity). However:*

a) *The rationale for selecting these two drugs should be clarified. How do their known molecular targets relate to glioma invasion pathways? While dasatinib and thapsigargin have been previously identified as anti-invasive agents, briefly linking their molecular targets to glioma invasion pathways would strengthen the justification for their use in this study.*

Response. Dasatinib and thapsigargin were selected because they perturb two mechanistically distinct pathways relevant to glioblastoma invasion, and because both were previously identified in our in vitro work as inhibitors of glioblastoma cell migration (Rosén et al., Communications Biology 2023). Dasatinib inhibits SRC family kinases, which regulate cytoskeletal remodeling, focal adhesion dynamics, and directed cell motility, processes central to glioblastoma invasion. Thapsigargin inhibits the SERCA pump, depleting ER calcium stores and activating the unfolded protein response; perturbations of intracellular calcium homeostasis are particularly relevant in glioma-initiating cells, which show heightened sensitivity to calcium signaling disruption (Wee et al., PLoS ONE 2014; Niklasson et al., Cancer Research 2017), and thapsigargin-based compounds have been explored clinically as targeted anticancer agents (Mahalingam et al., British Journal of Cancer 2016). Together these drugs provide complementary pharmacological perturbations, tyrosine kinase signaling versus calcium homeostasis, allowing evaluation of whether GlioTrace can capture mechanistically distinct effects on invasion dynamics.

b) *Importantly, there is no evaluation of standard-of-care therapies (radiation and temozolomide). Testing these agents, or their combinations, would improve translational relevance, especially in assessing treatment-resistant invasion phenotypes. Are the patient-derived glioma cells characterized in terms of MGMT promoter methylation status, radio-sensitivity or resistance, and whether they originate from primary or recurrent tumors?*

Response. Thank you for this helpful suggestion. We have incorporated standard-of-care treatment into the assay: tumor-bearing mice were irradiated in vivo (20 Gy), after which brain slices were prepared and exposed ex vivo to temozolomide during time-lapse imaging **(Fig. 6; Supplementary Fig. 13).** Radiation significantly reduced tumor cell motility and diffusivity. The combination of radiation and temozolomide additionally altered invasion phenotype composition, including increased branching cell frequency in some models, and HMM transition analysis revealed substantial rewiring of phenotype transition networks with increased state self-transitions, suggesting that radiation reduces phenotypic plasticity by constraining transitions between states. Effects on instantaneous migration speed were comparatively modest, consistent

with these therapies acting primarily as anti-proliferative agents rather than direct motility inhibitors.

Regarding molecular characterization, MGMT promoter methylation status and primary versus recurrent tumor origin are now provided for all six models in Supplementary Data File, Excel Sheet “HGCC Clinical Data”, allowing the observed invasion dynamics to be interpreted in the context of clinically relevant molecular features.

4. *The methods section is well-documented and reproducible, with clear reporting of software versions and imaging parameters. However, some clarifications are needed:*
   a) *Define the time-lapse imaging parameters more precisely. It is currently stated as "no more than 2 hours per frame." What was the exact interval used?*

Response. The time-lapse interval for each experiment is provided in Supplementary Data File (Excel Sheet 1). The median interval was 1.5 hours (range 0.85 to 1.95) intervals, reflecting a pragmatic trade-off between imaging speed and the need to monitor multiple specimens in parallel across slices, fields, Z-planes, and channels.

   b) *How many movies were acquired per PDCX model?*

Response. The number of time-lapse recordings per model is now reported in Supplementary Data File (Excel Sheet 1). In brief: U3013MG 138 slice movies from 25 mice, U3028MG 40 from 8 mice, U3054MG 25 from 5 mice, U3179MG 12 from 2 mice, U3180MG 69 from 13 mice, and U3220MG 22 from 7 mice. A full breakdown of mice, slices, movies, and analyzed ROIs is provided in the Supplementary Data File.

   c) *Describe the number of biological replicates (i.e., independent mouse brains per model). Imaging many ROIs per slice is valuable, but independent biological replicates are essential to support conclusions. (Mention: Hitesh)*

Response: The numbers of independently grafted mice per model are reported above and in Supplementary Data File (Excel Sheet 1). The dataset has a nested structure, patient-derived model → mouse → slice → movie/ROI → cell, providing replication at multiple biological levels. The invasion phenotypes observed in GlioTrace are consistent with endpoint phenotypes reported for these models in independent studies (Doroszko et al.; Krona et al.), supporting reproducibility. The lower mouse number for U3179MG reflects this model's strong enrichment for the low-motility round phenotype, which limited its informative value for dynamic invasion analyses.

   d) *What criteria were used to determine “optimal tumor growth” (line 420) as the starting point for imaging?*

Response. Imaging was initiated once tumors showed sustained bioluminescence signal increases across at least two consecutive IVIS measurements, indicating active growth. For models with more diffuse growth and slower IVIS signal changes, an additional criterion was applied: mice that had lost 5–10% from their peak body weight, consistent with established tumor burden. Final IVIS signal values for each mouse prior to brain slicing are reported in Supplementary Data File (Excel Sheet 1).

5. *In lines 129–130, the authors state that U3220MG shows meningeal growth and U3054MG perivascular growth. However, based on the images in Figure 1E and F, both appear to show meningeal invasion. Please clarify.*

Response: Thank you for noting this. The representative images selected for **Fig. 1E** and **F** were not optimally chosen to illustrate the distinction. U3220MG is a gliosarcoma-derived line that consistently shows strong subventricular and meningeal growth in our experience. U3054MG predominantly forms condensed growth along blood vessels, but can also migrate through the corpus callosum and cortex to secondarily reach the meninges. We have clarified this in the revised manuscript and selected more representative images where possible.

6. *Proliferation and cell death status for each PDCX model should be demonstrated at the imaging endpoint.*

Response. Thank you for this important comment. We have now added endpoint proliferation and apoptosis measurements for four PDCX models (U3013MG, U3054MG, U3180MG, and U3220MG) using Ki67 and cleaved caspase-3 immunostaining immediately after sectioning (day 0) and at the end of the imaging period (day 6) **(Supplementary Fig. 2).**

Apoptotic fractions remained low across models: U3013MG showed minimal change (1.7% at day 0 vs. 2.3% at day 6), as did U3054MG (5.7% vs. 7.1%). Modest increases were observed in U3180MG (2.3% to 9.5%) and U3220MG (8.6% to 19.3%) by day 6, but these values are consistent with ongoing tumor biology rather than widespread culture-induced cell death. Importantly, proliferative activity remained detectable across all models over the culture period (U3013MG: 23.6%→12.6%; U3180MG: 9.5%→33.0%; U3220MG: 20.3%→31.1%; U3054MG: 15.9%→32.9%), confirming that tumor cells remain biologically active throughout the imaging window. The ~30% EthD-1 positivity reported in the original submission principally reflects acute mechanical injury at the slice surface caused by vibratome sectioning, a well-recognized feature of organotypic preparations, rather than progressive tissue deterioration during culture.

To further characterize the host microenvironment over time, we performed longitudinal single-cell RNA sequencing of slice cultures at days 1, 3, and 6. This analysis resolved all expected brain cell populations at each time point, and hallmark gene set scoring revealed no significant upregulation of cell-death programs at day 3 or day 6 **(Fig. 2D).** Together these data support the conclusion that the slice microenvironment remains viable and transcriptionally stable across the GlioTrace imaging window.

7. *In lines 188–191, the authors describe that U3180MG cells alternate between vessel-associated and non-vessel-associated migration, indicating an "inherent ability to switch" between modes. What intrinsic features of the cells support this adaptability? Is it driven by cell-intrinsic properties or the tumor microenvironment?*

Revised. Thank you for this insightful question. To address it, we have extended the method to take the microenvironment into account **(Fig. 4,5, Supplementary Fig. 10)**. Disentangling cell-intrinsic plasticity from microenvironmentally driven switching is a central challenge in glioblastoma invasion biology, and we addressed it directly by extending the HMM framework to incorporate microenvironmental covariates into the transition model. In the revised analysis,

transition probabilities between invasion phenotypes are modeled as a function of both the current cellular state and local microenvironmental features, including proximity to blood vessels, microglial co-localization, and directional polarization of neighboring tumor cells. This covariate-dependent formulation separates a baseline transition rate - reflecting cell-intrinsic switching capacity - from context-dependent modulation by local tissue features.

The analysis reveals that transitions into perivascular translocating states are significantly associated with vascular proximity, consistent with blood vessels acting as permissive guidance structures for this invasion mode. In contrast, transitions involving branching and locomotive states show weaker dependence on these microenvironmental features, suggesting a larger contribution from cell-intrinsic plasticity. Critically, individual U3180MG cells alternate repeatedly between vessel-associated and non-vessel-associated migration within single trajectories, a pattern that cannot be explained by passive environmental guidance alone and demonstrates that mode-switching capacity is an intrinsic cellular property that microenvironmental context modulates rather than determines. Together these observations support a two-component model of invasion plasticity: a cell-intrinsic baseline switching rate that is biased toward particular states depending on local context. These analyses are shown in **Fig. 5 and Supplementary Fig. 10,** and the two-component framework is discussed explicitly in the revised Discussion.

8. *The interaction between glioma cells and blood vessels is well demonstrated. Are tumor cells physically interacting with perivascular pericytes or endothelial cells? What molecular features influence whether cells migrate via perivascular routes?*

Response. Thank you for this question. To investigate whether glioblastoma cells physically interact with pericytes and endothelial cells, we performed immunofluorescence staining for the pericyte marker CD13 at the imaging endpoint in U3013MG and U3180MG slices. CD13-positive pericytes were consistently found in close proximity to tumor cells, but without superimposed colocalization, consistent with tumor cells occupying the perivascular Virchow-Robin space rather than directly contacting pericyte cell bodies. By tracing individual tumor cells identified in the endpoint staining back through the time-lapse recordings, we confirmed that the same cells engaged in repeated physical contact with both pericytes and endothelial structures during perivascular migration, suggesting that multiple cell–cell contacts contribute to defining the invasion route **(Supplementary fig. 15D; Supplementary Video 7)**. The molecular determinants of perivascular route selection are addressed in our complementary study (Doroszko et al., 2025) and represent an important direction for future work.

9. *Regarding glioma–microglia/macrophage interactions: What is the role of microglia in determining glioma migration patterns? Could genetic or chemical inhibition of microglia alter glioma invasion behavior?*

Response: The covariate-dependent HMM framework allows us to estimate how proximity to microglia influences invasion state transitions without requiring experimental manipulation. Using this approach, we find that microglial co-localization is associated with increased transitions toward round and diffusely translocating states **(Fig. 5; Supplementary Fig. 10)**. To probe this relationship experimentally, we performed microglia depletion using clodronate treatment of U3013MG slices (Vidya et al., Nature Communications 2022). Under depletion conditions, the microglia-associated modulation of transition dynamics was reduced, while overall tumor cell migration speed remained largely unchanged **(Supplementary Video 5; Supplementary Fig. 9F-H).** These observations suggest that microglia can influence phenotypic state transitions

during invasion more strongly than bulk migration speed, although we interpret this perturbation result cautiously and present it as supportive rather than definitive.

10. *Cell segmentation is limited in the tumor core. Including a nuclear marker or dye would improve segmentation and tracking in dense tumor areas.*

Response. We agree that dense tumor core regions present segmentation challenges. GlioTrace is therefore optimized for the invasive margin, where individual cells can be reliably detected, tracked, and classified. Nuclear labeling was not included in the present experiments in order to preserve imaging channels for tumor cells and microenvironmental features during long-term live imaging, but inclusion of a nuclear marker represents a logical future extension for improving core segmentation. An annotated example distinguishing tumor core from invasive edge regions is now provided in the revised manuscript.

Minor Revisions
• The manuscript is dense in some areas, particularly the Results section. Breaking up long paragraphs and improving transitions between topics (e.g., movement dynamics → phenotype classification → HMM modeling) would enhance readability.
• Some figure panels (e.g., Figures 2L–M, 4F) should be more clearly described in the main text to help readers unfamiliar with computational modeling better interpret them.

Response. The Results section has been revised to break up long paragraphs and improve transitions between topics.

Reviewer #3 (Remarks to the Author): neuroimaging, AI

The authors developed GlioTrace to analyze 10x whole slide time lapse videos of PDCX specimens to analyze GBM invasion dynamics. Applying GlioTrace on PDCX specimens from 6 GBM patients, the authors identified three cell phenotypes (branching, trnaslocating, and locomotive). The authors observed different migration and phenotype transition patterns in different patients, and how drug treatment affect these patterns. GlioTrace can potentially analyze GBM invasion phenotypes and their modulation by therapy, and enabling functional profiling for precision oncology. Questions for authors:

1. *The use of a HMM to denoise CNN-predicted morphological classes raises concerns. In a standard HMM, the hidden state is meant to represent an unobserved biological process that causally generates the observed state. Here, hidden and observed states are defined over the same discrete classes, effectively using the HMM to correct CNN classification errors rather than uncovering latent dynamics, especially by setting emission probability to the CNN evaluation confusion matrix. However, CNN inference errors arise from model limitations, and do not represent meaningful causal relationships between cell states. This raises questions about the appropriateness of the HMM modeling framework in this context. Can the authors clarify the use of HMM? Does simpler approaches, such as direct counting, yield comparable insights? Have ablation studies been conducted to assess whether the HMM provides value beyond temporal smoothing of CNN outputs?*

Response. Thank you for this thoughtful critique. The concern is well-founded and we have addressed it through both model refinements and explicit ablation benchmarking.

On the modeling formulation: the original implementation used hard-assigned CNN class labels as observations, with the confusion matrix as the emission model. We agree this can appear circular. In the revised model, we replace hard labels with the full CNN softmax probability vector as the observation signal. This means inference operates on calibrated per-frame uncertainty rather than discrete assignments, and the HMM's role is explicitly to infer temporally coherent state trajectories from uncertain evidence - not to relabel CNN outputs. We additionally extend the transition model beyond a fixed matrix by incorporating microenvironmental covariates, including vessel proximity, microglial co-localization, and local cell polarization, through a multinomial logistic regression formulation. This covariate-dependent extension is a key reason to retain a probabilistic state-space model: it provides a principled framework for testing whether transitions are gated by local tissue context, which no simpler approach can provide.

On ablation: we benchmark three alternatives on the same datasets - naive per-frame CNN label counting, counting after majority-filter temporal smoothing, and the revised softmax-emission HMM **(Supplementary Fig. 7).** The probabilistic model yields more stable estimates of phenotype composition and transition rates across bootstrap replicates, and reduces spurious rapid switching events inconsistent with typical cell-shape persistence. In particular, bootstrap analysis across mouse-level datasets showed substantially greater drift in transition estimates for raw-count models than for the HMM, directly demonstrating that the probabilistic framework improves robustness to sampling noise rather than merely smoothing the output. Critically, the main biological conclusions are qualitatively consistent across all three methods, but the HMM provides improved robustness to sampling noise and is the only approach that supports hypothesis testing for microenvironment-dependent transition effects. These benchmarking results are reported in **Supplementary Fig. 7.**

2. *The description of the CNN used for cell state classification lacks sufficient detail, raising concerns about the scalability and generalizability of GlioTrace. The model architecture, training hyperparameters and configurations are not specified.How are the training, validation, and test sets constructed, and are they stratified by patient? Importantly, details regarding data processing are also missing. Cells are inherently elliptical or irregular in shape, and may overlap with neighboring cells. How are the images cropped and preprocessed for CNN input? Would the authors provide representative input images and class annotations, and how does surrounding context affect classification performance?*

Response. Thank you for this comment. We have substantially expanded the Methods and Supplementary Information to address each point raised.

Architecture and training: the CNN is a lightweight patch classifier consisting of sequential convolutional layers with ReLU activation and max pooling, followed by fully connected layers and a softmax output. All architectural details, number of layers, filter sizes, stride, padding, learning rate, optimizer, batch size, epochs, augmentation, and regularization, are now specified in the Supplementary Methods.

Input preprocessing: cells are detected using a Laplacian-of-Gaussian filter and a fixed-size image patch is extracted around each detected center. This approach standardizes classification around the cell body without requiring full segmentation. Neighboring cells and surrounding

microenvironment are intentionally included as contextual information in the patch. Representative input patches with class annotations are now shown in **(Supplementary Fig. 4)**, including challenging cases such as overlapping cells and ambiguous morphologies. Highly ambiguous regions are assigned to a separate crowded/unclassified category during training to prevent forced assignment into biological classes.

Validation: we performed leave-one-patient-out experiments in which the network is trained on five models and evaluated on the sixth. Classification accuracy remains high across all held-out models, demonstrating cross-patient generalization. We now report per-class recall for each held-out model explicitly **(Supplementary Fig. 4).** We also confirmed that cells cluster by morphological class rather than by patient identity in the learned feature space **(Figure 3),** supporting generalization beyond patient-specific visual signatures.

3. *The cell detection pipeline in GlioTrace appears to rely on classical image processing techniques. Can the authors report quantitative performance metrics for detection accuracy, such as mean average precision (mAP), benchmarked against manual annotations? How do detection errors impact downstream tracking, CNN classification, and HMM modeling? Have the authors considered benchmarking or replacing this step with a learned segmentation approach?*

Response. Thank you for this request. GlioTrace uses Laplacian-of-Gaussian center detection rather than full segmentation, a deliberate choice since the downstream pipeline requires reliable cell center localization rather than precise boundary delineation. In morphologically complex invasive regions with extensive protrusions, full segmentation can introduce instability that center detection avoids. To quantitatively assess performance, we manually annotated cell centers across representative subsets of multiple PDCX models and evaluated precision, recall, and F1 score within a defined spatial tolerance. We benchmarked against Cellpose (Stringer et al., 2021) and StarDist (Schmidt et al., 2018) with default parameters. Cellpose achieved high precision but reduced recall, particularly missing elongated or low-contrast invasive cells. StarDist showed high sensitivity but tended to over-segment branching morphologies. The LoG detector provided a balanced trade-off while remaining substantially faster. These comparisons are now reported in **(Supplementary Figure 5C-F).** Downstream robustness is strong: small localization offsets do not materially affect CNN patch classification, transient false-positive detections generate short trajectories that are filtered during track assembly, and the HMM integrates information across full trajectories making it robust to occasional missed frames. An ablation analysis confirming that reasonable perturbations of detection thresholds do not substantially alter phenotype composition or transition statistics is now included in **(Supplementary Fig. 7).** The pipeline is modular and alternative detectors including Cellpose and StarDist can be substituted through a documented interface in the Python implementation.

4. *GlioTrace is only validated on 6 PDCX models. This raises concerns regarding the scalability of the proposed model, as well as the generalizibility of the observations reported in the paper. Critical details regrading training / validation set split should be discussed further. Have the authors tested the model in separate, held-out PDCX lines to evaluate generalization beyond the training cohort?*

Response. Thank you for raising this. We addressed generalization at three levels. First, leave-one-patient-out CNN validation: training on five models and evaluating on the sixth yields high classification accuracy across all held-out models, demonstrating that the network learns generalizable morphological features rather than patient-specific signatures. Second, mouse-level holdout: holding out individual mice within each patient model showed stable performance, indicating the classifier is not overfitting to preparation-specific artifacts. Third, feature space analysis: cells cluster by morphological class rather than patient identity in the learned embedding, directly supporting generalization.

Beyond the number of patient-derived models, the platform operates at substantial observational scale: whole-slice imaging over approximately 100 time points generates 9000×9000-pixel movies from which thousands of cells can be tracked and analyzed, enabling robust estimation of state composition and transition structure. This imaging regime yields whole-slice longitudinal datasets at a scale sufficient for tracking and classifying thousands of individual tumor cells per experiment, which is essential for robust estimation of phenotype composition and state-transition structure.

Beyond machine learning validation, the experimental design provides replication across biological scales through a nested structure, patient → mouse → slice → ROI → cell, enabling separation of patient-level invasion patterns from preparation variability. Mouse counts, slices, ROIs, and tracked cells per model are now detailed in Supplementary Data File, Excel Sheet 1. We acknowledge that six models cannot capture the full spectrum of glioblastoma heterogeneity, but within the practical constraints of orthotopic PDCX invasion modeling, six independently derived lines spanning distinct invasion architectures from a larger clinical repertoire represents a substantial cohort, especially in the context of prior quantitative dynamic invasion studies, which typically use one or two models.

> *5) The analysis of GlioTrace seems to be focus on tumor cells and blood vessels, but doesn't model other normal cells at the margin or immune cells. How does this limited representation of the tumor microenvironment affect the generalizability of the findings, especially given the known importance of neurons, glial cells and immune cells in GBM infiltration?*

Response. Thank you for this important comment. The current implementation explicitly probes microglia and macrophages alongside vasculature, capturing the major innate immune component of the brain microenvironment **(Fig. 5)**. The cellular composition of the slice microenvironment is now characterized by longitudinal single-cell RNA sequencing of the full system **(Fig. 2)**. The absence of adaptive immune T cells reflects the immunodeficient mouse model rather than a conceptual limitation of the GlioTrace framework - the covariate-dependent transition model is inherently extensible to any labeled microenvironmental feature, and distances to neurons, astrocytes, pericytes, or other glial populations could be incorporated as covariates as fluorescent labeling strategies are expanded. We view the present study as a first systematic step toward quantitative modeling of tumor–microenvironment interactions at scale, and we have stated this explicitly in the revised Discussion. The immunological limitations are also acknowledged there, including the consequences for generalizability of our microglial findings to immunocompetent settings.

6. *How would GiloTrace be integrated into the clinical workflow for personalized treatment recommendation, and how does it compare to the current standard-of-care?*

Response. Thank you for this question. We have expanded the Discussion to address this directly while being appropriately cautious about clinical readiness.

The current standard-of-care for glioblastoma (surgical resection followed by radiotherapy and temozolomide) involves limited molecular stratification and no functional assays for invasion dynamics. GlioTrace is not proposed as a replacement for this standard, but as a complementary platform with three potential roles in increasing order of clinical proximity. Most immediately, it serves as a discovery tool for characterizing invasion dynamics across patient-derived model collections and identifying biologically meaningful dynamic subgroups not captured by molecular profiling. In a more translational setting, patient-derived tumor slices from surgical material could in principle be subjected to ex vivo profiling to assess invasion phenotypes and drug sensitivity. Most realistically near term, GlioTrace may function as a discovery engine that identifies molecular correlates of dynamic invasion states, such as the marker associations observed in our protein-level analyses, enabling inference of invasion phenotype composition from standard clinical specimens without requiring live imaging infrastructure. Each of these applications requires prospective validation before clinical implementation, and we have stated this clearly in the revised manuscript.

7. *All experimentation are performed using PDCX models. How reliable are these models to reflect how tumors behave in human? This raises concerns about the translational applicability of GlioTrace for patient-specific treatment guidance.*

Response. Thank you for this important question. We agree that PDCX models are approximations of human disease, and the revised Discussion explicitly addresses both their strengths and limitations. On the strengths side: these models are derived directly from patient tumor material without long-term in vitro adaptation, preserve major driver alterations and transcriptional heterogeneity, grow within the native mouse brain microenvironment, and exhibit hallmark glioblastoma invasion behaviors consistent with human histopathology and intravital imaging studies. On the limitations side: the models are grown in immunodeficient mice lacking adaptive immunity, species differences in stromal and vascular biology may influence microenvironmental interactions, and engraftment may impose selective pressures altering clonal composition. These limitations constrain direct extrapolation to patient-level treatment response. For these reasons, we do not position GlioTrace as an immediate clinical decision-making tool but as a platform for quantitatively dissecting invasion dynamics in a controlled setting, generating hypotheses and candidate biomarkers for subsequent validation in human clinical material.

Reviewer #3 (Remarks on code availability):

The code base is generally well written, with many functions clearly documented. However, it would benefit from improved organization and the inclusion of a README file with instructions for running the code. It is somewhat difficult to locate specific components of GlioTrace, such as the CNN training and inference pipelines. Some functions, including "customReadFunction_semseg.m", "customReadFunction.m", "customReadFunction3.m", "customReadFunction5.m", are repetitive and lack documentation, causing confusion. Additionally, while the use of MATLAB is understandable for the project, much of the machine

learning community has transitioned to Python. Relying on a proprietary language like MATLAB may pose barriers to wider adoption and long-term maintenance of the tool.

Response. Thank you for the careful assessment of the code base. We have substantially reorganized the repository in response to each point raised. The repository now includes a comprehensive README with step-by-step instructions covering installation, data formatting, CNN training, inference, tracking, and transition modeling. The directory structure has been reorganized to clearly separate preprocessing, CNN training, inference, tracking, and modeling components. Redundant and ambiguously named functions, including the customReadFunction variants, have been consolidated, renamed descriptively, and fully documented. Inline documentation and docstrings have been expanded throughout, and example configuration files, example datasets, and expected outputs are now provided.

In response to the concern about MATLAB accessibility, we have ported the full GlioTrace pipeline to Python, which is now the official distribution. The Python package includes modular CNN training and inference (PyTorch-based), tracking and feature extraction modules, probabilistic state-transition modeling, full API documentation, and a tutorial notebook demonstrating end-to-end usage. The MATLAB implementation remains available for reproducibility of the originally submitted results, but future development will focus exclusively on the Python version.

Reviewer #4 (Remarks to the Author): GBM, brain imaging, some computational modelling, spatial

This study introduces GlioTrace, an ex vivo imaging and AI-based analytical framework for tracking glioblastoma invasion dynamics in patient-derived xenograft (PDCX) brain slices. The authors combine confocal microscopy with computational modeling using convolutional neural networks and Hidden Markov Models to identify distinct invasion modes and quantify patient-specific variations in invasion plasticity. Using six PDCX models, they demonstrate different invasion patterns (bulky/perivascular vs. diffuse), characterize morphological transitions between branching, translocating, and locomotive cell types, and show that targeted therapies can selectively modulate invasion phenotypes with spatially and temporally distinct drug responses.
Overall, I find this work interesting in its approach to analyzing glioblastoma invasion dynamics, but I am unsure about the quality of the model system and whether it adequately recapitulates in vivo conditions.

Response. Thank you for this balanced assessment. We have addressed each major concern through new experiments and analyses as described below.

Major Points

1. The model system itself has made an interesting modification to previous approaches. While the authors now use organotypic brain slices from previously engrafted tissue, previous work has used organotypic slices with subsequent injection of cancer cells. The fundamental limitations of slice culture systems should be addressed, and the improvement over existing methods should be clearly demonstrated. The authors should directly compare their approach to existing slice

culture methods and intravital microscopy to demonstrate clear advantages and validate that their observations reflect genuine biological processes rather than culture artifacts. Specifically, the authors should validate that their observed patient-specific invasion patterns are comparable to in vivo behavior, potentially using two-photon microscopy (e.g. as in Griveau et al., Cancer Cell 2018). This is particularly important because strict perivascular invasion patterns can also be observed in unhealthy organotypic brain slices as an artifact rather than a true biological phenomenon.

Response. Thank you for these important comments. We address the three elements of this concern in turn.

Comparison to intravital microscopy: We view GlioTrace as complementary to intravital imaging rather than a replacement. Intravital microscopy remains the gold standard for truly in vivo observation but is typically constrained to superficial cortical windows, limited fields of view, and sparse temporal sampling due to practical considerations. GlioTrace offers two specific advantages critical to the computational goals of this study: access to deeper brain regions inaccessible to cranial window approaches, and dense longitudinal sampling of approximately 100 time points per series. This density is essential because our transition modeling aims to resolve phenotypic switching dynamics, not just endpoint distributions, and transition rate estimates become substantially less stable with sparse sampling. We have clarified this complementary positioning in the revised manuscript and added a comparison table summarizing experimental modalities and their analytical scope (Supplementary Data File, Excel Sheet "Comparison Table").

Comparison to post hoc slice injection: Our choice to image orthotopically established tumors rather than inject cells directly into naïve slices was motivated by the need to preserve a realistic spatial distribution of tumor cells across anatomical structures and avoid the confounding effects of injection-site geometry and acute tissue injury. To directly address this point, we performed a side-by-side comparison between orthotopic slices and post hoc injection preparations. The injection condition yielded substantially higher fractions of round low-motility morphologies and a distorted transition structure, consistent with reduced tissue integration and acute injury effects **(Supplementary Fig. 15; Supplementary Video 5).** These data support the superiority of the orthotopic approach for dynamic invasion analysis.

Perivascular invasion as genuine biology, not artifact: We directly address the concern that perivascular patterns may reflect unhealthy tissue through three lines of evidence. First, perivascular invasion is strongly model-dependent and reproducible across independent animals, it does not appear uniformly across all slice cultures as would be expected for a generic deterioration artifact. Second, the diffusely invading model U3180MG, which shows non-perivascular invasion in vivo, does not convert to strict perivascular behavior under slice conditions, its dynamics remain consistent with its in vivo growth pattern and independent endpoint histology. Third, single-cell RNA sequencing of slice cultures across the imaging window shows that tumor cells occupy a broad spectrum of established glioblastoma cell states and do not collapse into a single stressed or dying transcriptional state. Together these arguments are now stated explicitly in the revised manuscript alongside side-by-side comparisons between time-lapse readouts and endpoint measurements.

2. The scalable approach is interesting, but it remains unclear how tumor cells and their microenvironment are affected by slice culture conditions. Supplementary Figure 1D shows that

approximately 30% of cells are dead (EthD-1 positive), which raises serious concerns about tissue health. Previous work has established that adult neurons cannot be properly maintained in slice cultures, which is why organotypic hippocampal slice cultures typically use embryonic tissue. The fragmented appearance of vasculature in some images further suggests compromised tissue integrity. The authors should provide comprehensive characterization of the health and function of all microenvironmental components over time, including vascular cells, neurons, glial compartments, and immune cells.

Response. Thank you for raising this critical point. We have substantially expanded our characterization of slice health and microenvironmental composition.

On the EthD-1 signal: the ~30% positivity in the original figure reflects acute mechanical injury at the slice surface from the vibratome sectioning procedure, a well-recognized feature of organotypic preparations that is spatially restricted to the cut surface and does not represent progressive tissue deterioration. To demonstrate this directly, we quantified apoptosis (cleaved caspase-3) and proliferation (Ki67) at day 0 and day 6 across four models. Apoptotic fractions remained low and largely stable: U3013MG 1.7%→2.3%, U3054MG 5.7%→7.1%. Modest increases in U3180MG (2.3%→9.5%) and U3220MG (8.6%→19.3%) by day 6 remain within ranges consistent with ongoing tumor biology. Proliferative activity remained detectable across all models (U3013MG 23.6%→12.6%; U3180MG 9.5%→33.0%; U3220MG 20.3%→31.1%; U3054MG 15.9%→32.9%), confirming continued biological activity throughout the imaging window.

For comprehensive microenvironmental characterization, we performed longitudinal single-cell RNA sequencing at days 1, 3, and 6, profiling approximately 150,000 cells in total. Unsupervised clustering resolved all expected brain cell populations at every time point, neurons, astrocytes, oligodendrocyte-lineage cells, microglia, endothelial cells, and perivascular stromal cells, without evidence of selective lineage loss. Transcriptional programs were largely stable between days 1 and 3; by day 6 a modest upregulation of interferon and stress-associated signatures was detectable, consistent with a mild late culture response, but global activation of apoptotic, hypoxic, or necrotic programs was absent. By day 6, a modest interferon- and stress-associated transcriptional response was detectable, but this occurred in the setting of preserved cell-type composition, maintained proliferative activity, and absence of global cell-death signatures, and we therefore interpret it as a mild late culture response rather than evidence of progressive tissue collapse. These findings were corroborated at the protein level by immunostaining for NeuN (neurons), GFAP (astrocytes), and CD11b/Iba1 (myeloid cells), all of which remained readily detectable across the imaging window. While long-term maintenance of adult neuronal physiology in slice culture is challenging, the 4–6 day imaging window used here is compatible with neuronal and glial populations that remain stable.

Regarding fragmented vasculature: this morphology is spatially restricted to tumor regions while adjacent tumor-free vasculature in the same slice remains intact, is model-specific and reproducible across independent animals, and is recapitulated in independent endpoint histology from the same PDCX models in vivo. These observations collectively indicate tumor-induced vascular remodeling rather than generic slice deterioration.

3. While the drug responses are interesting, it is unclear whether these effects are mediated through the microenvironment or represent direct effects on cancer cells. The authors should distinguish between these mechanisms and demonstrate that observed responses are physiologically relevant rather than artifacts of the slice culture system.

Response. Thank you for this important question. We have expanded the analysis to distinguish between direct tumor cell effects and microenvironment-mediated contributions using two complementary approaches. First, covariate-dependent transition modeling explicitly tests for microenvironmental mediation. Incorporating vascular proximity and microglial density as covariates, we find that dasatinib-associated reductions in transitions into highly motile states are largely preserved across different microenvironmental contexts, arguing against a purely microenvironment-driven mechanism **(Supplementary Fig. 14D–E).** Second, single-cell transcriptomic profiling of treated slices (10x Genomics) shows that tumor cells exhibit a substantially stronger transcriptional response than the surrounding host compartment, with enrichment of stress-associated and mesenchymal programs and depletion of proneural and OPC-like signatures. In contrast, the number and magnitude of differentially enriched pathways in host cells are markedly lower **(Supplementary Fig. 14A–C).** Together, these analyses, along with a known function in dasatinib inhibiting GBM cell migration (e.g. Huveldt et al, 2013), support that the observed drug responses include a substantial tumor cell–intrinsic component, with additional context-dependent modulation by the microenvironment, rather than representing nonspecific artifacts of the slice culture system.

4. The number of xenografted mice used for each experiment is unclear, as mostly slice and cell numbers are reported. The authors should provide exact numbers of mice used and compare preparation heterogeneity between mice to ensure that observed patient-specific differences reflect genuine biological variation rather than preparation artifacts.

Response.

Thank you for this important point. Mouse counts, slice numbers, ROIs, and tracked cells per model are now reported in full in Supplementary Data, Excel Sheet, “Experiment Imaging Parameters”. Across the six models: U3013MG 25 mice, 138 slice movies; U3028MG 8 mice, 40 movies; U3054MG 5 mice, 25 movies; U3179MG 2 mice, 12 movies; U3180MG 13 mice, 69 movies; U3220MG 7 mice, 22 movies. The dataset has a nested structure, patient → mouse → slice → ROI → cell, and the appropriate unit of analysis varies by question. Our mixed-effects modeling framework explicitly accounts for this hierarchy. Variance component analysis, now reported in the revised manuscript, confirms that model-specific behavioral signatures are robust to inter-animal variability.

Independent evidence for inter-mouse reproducibility comes from three converging sources. In the HGCC Phenobank study (Krona et al., bioRxiv 2025), phenotypic scoring across 449 mice from 76 HGCC cultures demonstrated that invasion phenotypes are highly reproducible within cell lines, with phenotypic distances between mice from the same line significantly more compact than between lines (K-S test, $p = 0.004$). In Doroszko et al. (Nature Communications 2025), transcriptional invasion state composition was shown to be reproducible across independently grafted animals. And within the present study, phenotype composition and transition structure are consistent across slices and mice from the same model. We acknowledge that the mouse numbers in the GlioTrace imaging experiments are modest relative to the full Phenobank cohort, reflecting the practical demands of dense longitudinal time-lapse acquisition, but the convergent

evidence across three independent studies supports the conclusion that patient-specific invasion patterns are stable biological properties of these models.

Minor Points

1. *How are "core" and "periphery" regions exactly defined in this study? Clear criteria should be provided. (Hitesh)*

Response. Thank you for this comment. The tumor core refers to regions of very high cell density where individual cells cannot be reliably segmented or tracked due to signal overlap. The invasive periphery refers to surrounding regions where individual tumor cells can be resolved, detected, and tracked. This distinction is operational and based on segmentation feasibility rather than a sharp anatomical boundary, as glioblastoma infiltration occurs along a continuous spatial gradient. Annotated examples clearly illustrating representative core and peripheral regions are now provided in the revised manuscript.

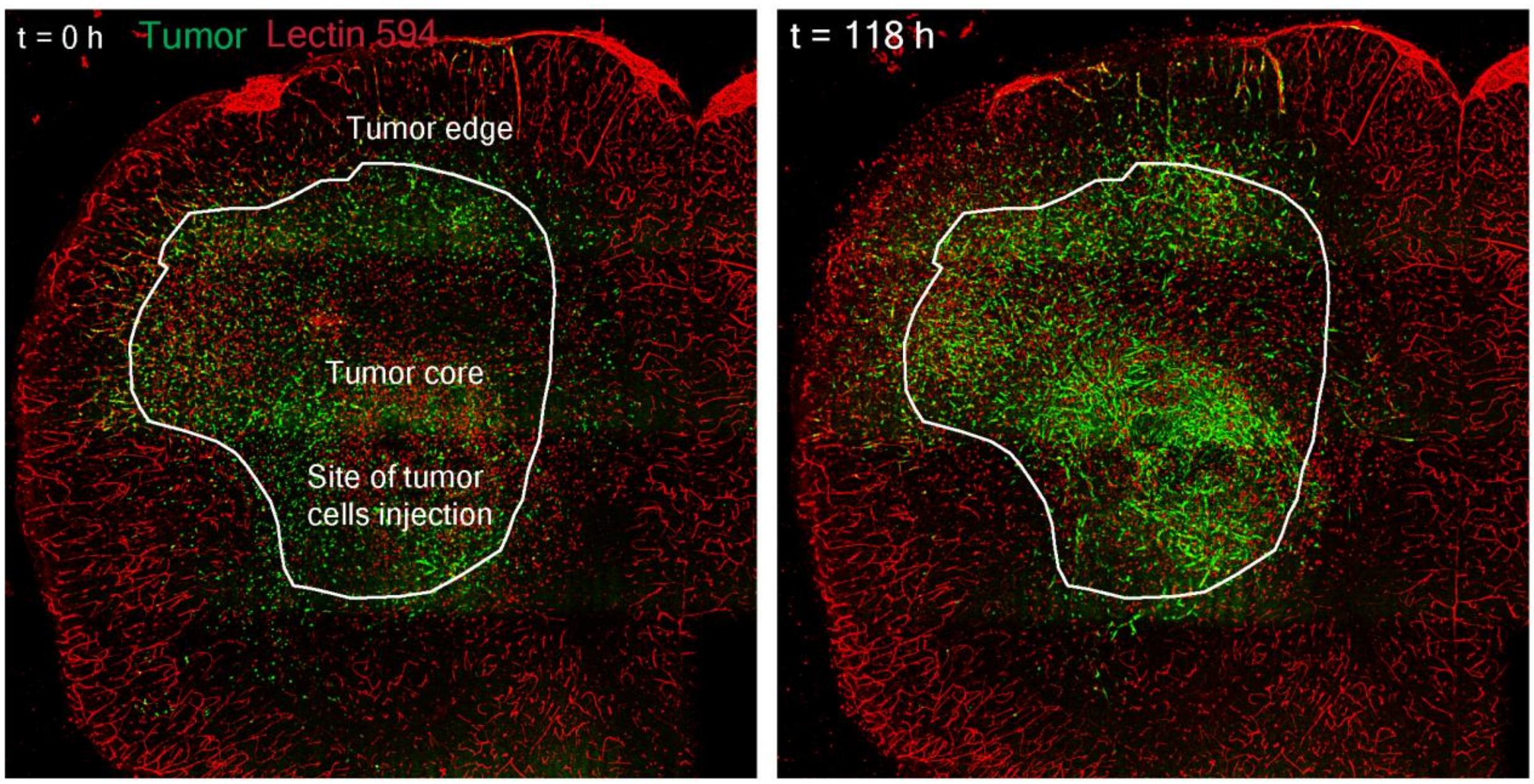

2. *How are "junk" cells defined, and can the authors provide more examples? Are these cells alive and functional, or are they artifacts of the slice culture system? This distinction is important for interpreting the biological relevance of the findings.*

Response: Thank you for raising this. The term "junk" in the original submission referred to image patches that could not be reliably assigned to a biological morphology class, regions with severe cell overlap, imaging debris, or areas without a clear cell center. These patches were assigned to a separate category during CNN training to prevent forced misclassification of ambiguous regions into biological classes. They are not a distinct cell type but rather an image-level quality flag. In the revised manuscript we have renamed this category "crowded" to more accurately reflect its meaning, and we provide representative examples in Supplementary Fig. 4 to illustrate what these patches look like and how they differ from the five biological morphological classes.

POINT-BY-POINT RESPONSE TO REVIEWERS

Reviewer #1

The authors have addressed my comments. Congratulations to the authors on this interesting and informative study.

Response: Thank you for the kind words and the careful assessment of our paper.

Reviewer #2

The authors are commended for the thorough revisions to the manuscript. While Reviewer #2's concerns have been largely addressed, several aspects would benefit from further strengthening. These represent relatively minor revisions that would enhance the overall quality of the study and increase the utility of GlioTrace as a tool for the broader scientific community.

1) *Given that the central contribution of this work is the development of GlioTrace as a scalable analytical tool, it is essential to ensure that the software and methodology are fully accessible and user-friendly for the scientific community. Improving the GitHub repository, with clear organization, comprehensive documentation, step-by-step tutorials, and example datasets, will greatly enhance reproducibility and adoption. In addition, the development of a more user-friendly interface or packaged software for analysis would further increase its impact and usability across a broader range of researchers.*

Response: Thank you for the kind words and the suggestion. We have now updated our repository with the following:

- A user manual with step-by-step instructions
- Improved installation guide in the README file
- Framework overview as a flowchart, illustrating the needed inputs at every step and the generated output from running class methods
- A link to example data uploaded to a HuggingFace repository + a Jupyter notebook `demo_with_example_data.ipynb` for an example of how to run the pipeline with the provided data
- An additional Jupyter notebook `class_run_example.ipynb` with example use-cases and parameter combinations for each step

The code base is also extensively documented with descriptions of parameters and returns for each function.

After cloning the repository into a local folder, the software can be installed using pip with a single command (pip install .). We agree that publishing GlioTrace to PyPI would further broaden the tool's accessibility across the research community, and we have added this as a priority for future releases.

GlioTrace is currently designed to be run through Jupyter notebook, with annotated step-by-step cells for each part of the pipeline to aid users less familiar with Python, while allowing flexibility for more experienced users. Pipeline functionality is wrapped into high-level class functions that execute multiple operations automatically under the hood. For example, calling `run_tracking` performs cell detection, morphology classification, and tracking in a single step, without requiring the user to execute each stage manually.

> ***2)*** *While it is important to clearly describe how the glioma models were generated and analyzed, particularly in the context of endogenous tumor growth within intact brain tissue. The use of orthotopic or genetically engineered tumor models combined with tissue slice analysis is not, by itself, novel. Therefore, the authors should clarify how their approach advances beyond existing methodologies. Please revise the statement in lines 86–89 and expand the Discussion accordingly to better position the innovation of the model.*

Response: Thank you for the comment, we agree that the statement needs additional nuance and have revised lines 86-89. The most central innovation of GlioTrace lies not in how the tissue is prepared, but in how it is analyzed: (1) automated morphological classification of thousands of individual cells using convolutional neural networks, applied across the full slice field-of-view rather than within restricted imaging volumes; (2) covariate Hidden Markov modeling of phenotype transition dynamics, which is not performed in any prior brain slice or intravital imaging study; and (3) systematic application of this pipeline across six patient-derived HGCC models, enabling cross-patient comparison. By contrast, prior intravital approaches (Venkataramani et al. 2022; Schubert et al. 2024) are restricted to the cranial window, limiting imaging to superficial cortical regions and precluding whole-section population-level sampling; and Schubert et al. (2024) explicitly note that their three-photon approach is limited by "relatively slow acquisition speed" and restricted volumetric field-of-view. Alieva et al. (2019) and Comba et al. (2022) use syngeneic mouse GPC lines (NPA cells), whereas GlioTrace is applied to patient-derived HGCC cultures that preserve the heterogeneous biology of human GBM.

Accordingly, we have revised lines 86-89 as described in the manuscript and have expanded the Discussion (below) to further contextualize our work.

> ***3)*** *Several points addressed in the rebuttal letter should be explicitly incorporated into the manuscript, rather than remaining only in the responses. For example, the discussion of similarities with previously described migration modes (Response 1a and 1c) and the*

rationale for drug selection (Response 3a) should be clearly integrated into the main text to improve transparency and context.

Response: Thank you for this valuable comment. We have now incorporated the three pieces of content identified in our previous rebuttal into the main text of the manuscript:

(i) First, regarding the correspondence between GlioTrace phenotypes and previously described invasion modes (previously in responses 1a and 1c), we have expanded the Discussion paragraph on invasion phenotype correspondence to explicitly name and cite the prior studies that defined each mode, to clarify which GlioTrace phenotypes map to which previously described behaviors, also underscoring that GlioTrace enables the scalable computational characterization of these behaviors across brain regions. We have also added our observation that branching cells are enriched following standard-of-care treatment (radiation + temozolomide), which was previously mentioned only in the rebuttal response.

(ii) Drug selection rationale (previously in response 3a): We have expanded the sentence introducing dasatinib and thapsigargin in the Results section to more fully explain why each compound was selected, with citations to our prior in vitro migration screen (Rosén et al. 2023), the established mechanistic literature for each drug, and the clinical context. This information was previously only in the rebuttal and we agree it should be in the paper.

4) The authors state that GlioTrace-derived migration phenotypes correspond to well-established histopathological invasion patterns. To strengthen this claim, it would be highly valuable to include representative H&E histological images alongside the immunofluorescence data. This would facilitate interpretation and provide a clearer link between dynamic behaviors and established pathological features, supporting statements such as the correspondence between perivascular translocation and vessel co-option, or diffuse migration and parenchymal infiltration.

Response: We thank Reviewer #2 for this constructive suggestion. We agree that representative histological images provide an important static reference for interpreting the dynamic invasion phenotypes quantified by GlioTrace. We have therefore added H&E and immunohistochemical/IHC reference panels that compare the slice-culture imaging data with corresponding in vivo PDCX tumor histology.

Specifically, Supplementary Figure 1 now combines representative live-imaging examples with histological validation: panels A-B document longitudinal lectin-594 vessel staining in U3013MG slices; panel C shows condensed/perivascular growth in U3028MG; panel D shows diffuse growth in U3179MG; and panel E shows H&E and STEM121 staining from U3013MG and U3028MG, comparing in vivo PDCX tumors with ex vivo PDCX slices at day 0 and day 6. These panels link the dynamic GlioTrace readouts to preserved tissue architecture and human tumor-cell distribution in the slice assay.

In addition, Supplementary Figure 2 now provides a broader side-by-side histological reference panel across four PDCX models, U3013MG, U3180MG, U3054MG, and U3220MG.

For each model, H&E, NuMa, and STEM121 staining of the corresponding in vivo PDCX tumor are shown alongside day 0 and day 6 slice-culture images stained for STEM121 and lectin-594. This comparison illustrates both perivascular/bulky and diffuse invasion architectures across the model panel and shows that the ex vivo slice assay preserves key tumor-cell distributions and vascular relationships observed in the parental in vivo tumors.

Together, these additions provide the requested representative H&E histological context alongside immunostaining and fluorescence imaging. They support the interpretation that GlioTrace-derived phenotypes, including perivascular translocation/vessel-associated invasion and diffuse parenchymal infiltration, correspond to established histopathological invasion patterns rather than being solely image-analysis categories.

As additional context, the broader histopathological and molecular characterization of the HGCC/PDCX invasion phenotypes, including perivascular and diffuse invasion patterns (at endpoint, non-temporal), has been described in our previous work (Doroszko et al., Nature Communications 2025, https://doi.org/10.1038/s41467-025-61999-1; Krona et al., bioRxiv 2025, https://doi.org/10.1101/2025.03.25.645260), which is cited in the manuscript. The new Supplementary Figures 1 and 2 are therefore intended to provide the requested representative H&E/IHC context within the present study, while the prior HGCC Phenobank analysis provide the broader model-level characterization.

> *5) Moreover, clearer acknowledgment of limitations related to sample size and the lack of direct clinical correlation would improve the robustness and balance of the conclusions.*

Response: We thank the reviewer for this suggestion. The Discussion now contains a dedicated limitations paragraph that directly and explicitly addresses both points.

Regarding **sample size**, we state:

"The current study characterizes six PDCX models selected to represent opposing ends of the GBM invasion spectrum; this cohort is sufficient to demonstrate patient-specific differences in invasion dynamics and drug response but is too small for statistically powered conclusions regarding associations between molecular subtype, driver alteration, and invasion phenotype. Larger panels will be required to establish whether reproducible dynamic invasion subgroups exist beyond the perivascular and diffuse extremes, and to identify molecular predictors of invasion plasticity."

Regarding **clinical correlation**, we state:

"The ex vivo system used here does not directly capture clinical outcomes such as survival or recurrence. While the HGCC PDCX collection used in this study has been shown to reproduce clinically relevant histopathological invasion patterns, and xenograft survival correlates with patient survival in the broader HGCC Phenobank, a direct link between GlioTrace-derived invasion dynamics and patient clinical trajectories remains to be established in prospective cohorts."

We believe these statements provide the explicit and balanced acknowledgment of limitations requested.

Reviewer #2 on behalf of Reviewer #4

We thank Reviewer #2 for assessing the previous Reviewer #4 comments on behalf of the editor. In the responses below, we address the residual points that Reviewer #2 and the editor identified as requiring further action in this revision. Where Reviewer #2 indicated that a prior concern was already addressed, we note this briefly and do not repeat the full prior rebuttal.

Regarding points 1 and 2 [made by Reviewer 4 in the previous round, our clarification] *I believe the authors have substantially addressed the reviewer's concerns, and I do not consider two-photon microscopy to be essential for the scope of this manuscript. The primary objective of the study is to establish and characterize an organotypic glioma slice platform rather than to replicate all aspects of in vivo imaging approaches. Moreover, two-photon microscopy can be performed for a few hours and is limited to the cranial window. That said, the manuscript would be significantly strengthened by additional histopathological validation of tissue integrity and microenvironmental preservation during culture. Specifically, I recommend including H&E staining and immunohistochemical analyses for markers of key microenvironmental components, such as CD31 and SMA (vasculature), Nestin and SOX9 (glioma cells), and CD3 and CD45 (immune populations) that have not yet been included. These analyses should compare slices at baseline (day 0) and at the experimental endpoint (day 6), alongside FFPE coronal sections obtained directly from the corresponding in vivo tumors. Such experiments would help determine whether the organotypic slices maintain the cellular architecture and composition of the parental tissue over time and would address concerns regarding potential culture-induced artifacts. In addition, these studies would directly complement the reviewer's concern regarding the approximately 30% EthD-1–positive cells reported in Supplementary Figure 1D. Incorporating multiplex IF for proliferation and apoptosis markers, such as Ki67 and cleaved Caspase-3, and the markers mentioned above would further define whether cell death or compensatory proliferation occurs during culture and identify the specific cellular compartments affected.*
Overall, these analyses would provide a more comprehensive assessment of tumor tissue viability and microenvironmental stability, thereby strengthening confidence that the observed invasion patterns reflect biologically relevant processes rather than artifacts of ex vivo culture.

Response: We thank the reviewer for this detailed and constructive recommendation. We agree that histopathological and microenvironmental validation is important for interpreting the dynamic invasion phenotypes measured by GlioTrace.

In the revised manuscript, we now more clearly point to the viability and microenvironmental validation already included in Supplementary Figures 3 and 4. These data include Ki67 and cleaved caspase-3 staining across multiple PDCX models, as well as co-staining of cleaved caspase-3 with markers for neurons (β-III tubulin), astrocytes

(GFAP), and myeloid cells (CD11b). Together, these analyses show that proliferation remains detectable, apoptosis does not markedly increase across the culture window, and key brain microenvironmental compartments are preserved.

To further strengthen the vascular characterization requested by the reviewer, we have added new CD31 and CD13 immunofluorescence staining of U3013MG organotypic slices at day 0 and day 6 to Supplementary Figure 4. CD31 labels endothelial cells, while CD13 labels CNS pericytes, including capillary-associated pericytes, and was therefore selected as a more suitable pericyte marker than SMA for this context. These new data demonstrate preservation of endothelial and pericyte-associated vascular compartments across the 6-day culture period. In addition, as described in our response to Reviewer #2 Comment 4, we have added H&E, STEM121, and NuMa reference panels (STEM121 and NuMa are used as anti-human tumor cell markers) comparing corresponding in vivo PDCX tumors with day 0 and day 6 slice cultures in Supplementary Figures 1 and 2.

We note that standard FFPE processing of the organotypic slices is technically challenging because the membrane-supported slice tissue is fragile and prone to fragmentation during paraffin processing. We therefore use fixed whole-mount slice immunofluorescence, which is the standard approach for preserving spatial structure in organotypic slice cultures.

Regarding immune and glioma-cell markers, the xenograft model used here is the athymic nude mouse, which lacks functional T cells; CD3 staining would therefore be expected to be negative by model design. We used **CD11b** rather than CD45 because the relevant immune compartment in this setting is predominantly myeloid, and CD11b more directly labels microglia/macrophages. Glioma-cell identity is established by stable GFP expression and confirmed by STEM121 staining, which specifically labels human cytoplasm and distinguishes patient-derived tumor cells from mouse host tissue. The broader stemness and transcriptional identity of the HGCC lines, including Nestin and SOX2 expression profiles, have been described in our previous HGCC Phenobank studies, which are cited in the manuscript.

Taken together, the new CD31/CD13 vascular staining, the existing viability and microenvironmental marker analyses, and the new in vivo-versus-slice H&E/IHC reference panels provide a substantially strengthened validation of tissue integrity, cellular architecture, and microenvironmental preservation across the imaging window.

3. The authors have addressed this [R4 point 3, our clarification] concern by performing additional analyses, including covariate-dependent transition modeling to evaluate migration behavior across different microenvironmental niches and single-cell transcriptomic profiling of treated slices. These analyses support the conclusion that microenvironmental factors do not solely drive the observed drug responses. However, additional functional validation would further strengthen this interpretation. For example, migration assays could be performed in explant slices derived from transgenic models with microglial depletion or dysfunction, or by comparing responses in immunocompetent versus immunodeficient hosts. Given the well-established role of microglia as key regulators of glioma invasion, such experiments would

help distinguish tumor cell–intrinsic effects from microenvironment-mediated mechanisms and further validate the physiological relevance of the findings.

Response: We thank the reviewer for these suggestions. We agree that the proposed experiments are interesting and would be valuable in future work. However, we consider them beyond the scope of the present revision, particularly in light of the editorial decision not to request further functional experiments.

4. Reviewer concerns were addressed; no further comments.

Response: We thank the reviewer for confirming that this point was addressed.

Reviewer #2 on behalf of Reviewer #4, Minor points

1. The criteria [core vs edge areas, our clarification] used were provided in the new manuscript version.

Response: We thank the reviewer for noting that this point has been addressed in the revised manuscript.

2. The authors clarified that the original 'junk' cell category is in the new manuscript version. However, if feasible, it would be valuable to characterize these regions further using live/dead staining or apoptosis markers. Such analyses would help determine whether these cells represent viable but densely packed tumor cells, stressed cells, or non-viable cellular material, thereby strengthening the biological interpretation of this classification and excluding potential artifacts related to tissue preparation or culture conditions.

Response: We thank the reviewer for this question. The crowded class (called 'junk' in an earlier version) captures GFP-positive images with morphologies that do not correspond to any of the five GlioTrace states. In practice, this class is dominated by regions of dense GFP signal where two or more cells overlap and cannot be individually resolved, as explained in the Methods. Immunostaining data already available from our validation experiments show that cells in dense regions are GFP-positive, Ki67-positive, and STEM121-positive, indicating that they are viable, proliferating human tumor cells rather than dead or stressed material. We have now provided qualitative examples illustrating this characterization in Supplementary Figure 5E.

Reviewer #3, Code Availability - MATLAB version accessibility

The authors have made an excellent effort to improve the source code. The updated repository contains detailed documentation and comments throughout the code, along with a notebook to demonstrate usage. Please make sure the MATLAB version of the code remain accessible, as I wasn't able to find a link to it.

Response: We thank Reviewer #3 for the kind words and appreciate them taking the time to look through the new repository. The MATLAB version can still be found in the old repository at https://github.com/shipsauce/GlioTrace_repository.git

POINT-BY-POINT RESPONSE TO REVIEWERS' COMMENTS

Reviewer #2 (Remarks to the Author):

The authors have addressed my comments, and I have no further concerns.

Reviewer #3 (Remarks to the Author):

I thank the authors for the response. They have addressed my concerns.

Response: We sincerely thank Reviewer #2 and Reviewer #3 for their careful evaluation of our revised manuscript and for confirming that our responses have adequately addressed their concerns. We greatly appreciate their constructive feedback throughout the review process, which has helped us improve the clarity and quality of the manuscript.
