# Supplementary material for: Reconstructing the single-cell spatiotemporal dynamics of glioblastoma invasion
Source: Nat Commun. 2026 Nov 2;17:11245. doi: 10.1038/s41467-026-78908-9 (PMC13728213; doi:10.1038/s41467-026-78908-9)
Supplement: Supplementary file 3 [file 41467_2026_78908_MOESM1_ESM.pdf]

Supplementary Information

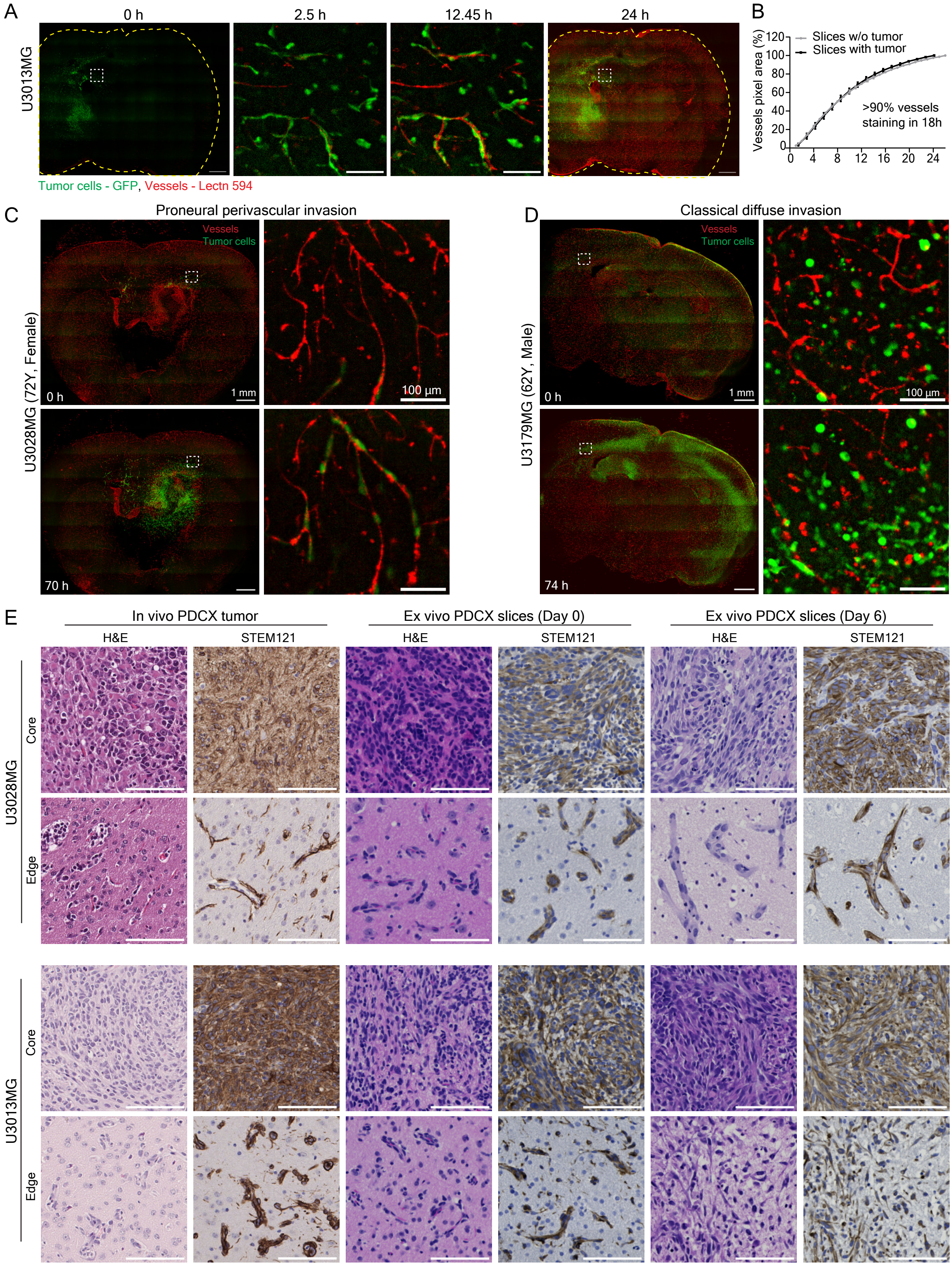

Supplementary figure 1. Validation of TL-DyLight 594 bioavailability to vessels and relative culture stability in longitudinal slice culture. (A) Representative time-lapse confocal images showing real-time lectin-594 staining of vessels (red) in live U3013MG tumor slice culture (tumor cells, green). (B) Percent pixel area of lectin-594-stained vessels in slice culture with or without tumor (n = 4 slices from 2 brains). Data represent mean ± SD. (C–D) Representative first and last time-point confocal images showing (C) perivascular migration by proneural subtype U3028MG glioblastoma tumor cells (72-year-old female) and (D) diffuse migration by classical subtype U3179MG glioblastoma tumor cells (62-year-old male) in slice culture. (E) Representative H&E and IHC images of tumor core and invasive edge area from ex vivo U3028MG and U3013MG slices at day 0 and day 6 compared to corresponding in vivo PDCX tumor. Scale bar 100 μm.

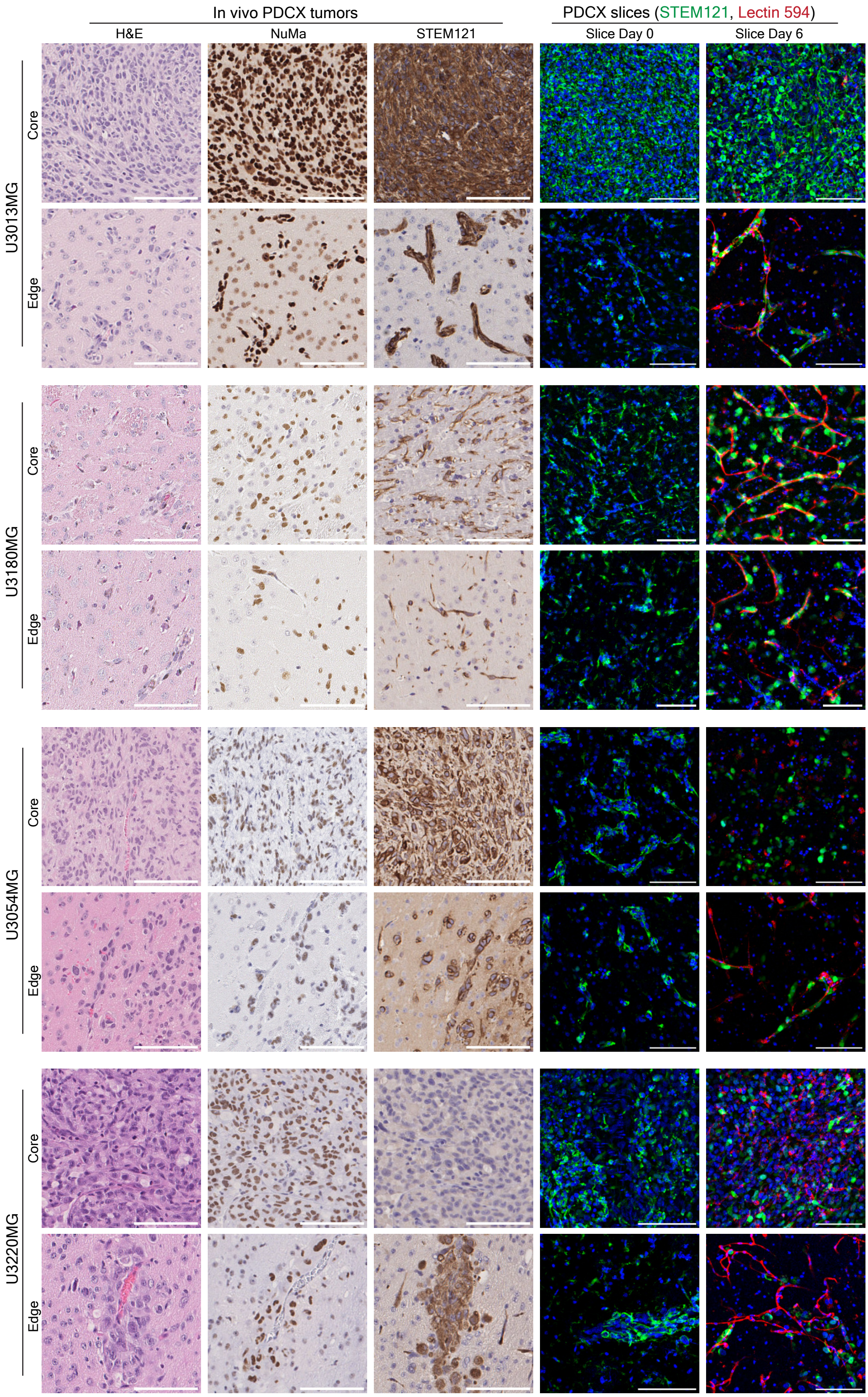

In vivo PDCX tumors
PDCX slices (STEM121, Lectin 594)
H&E
NuMa
STEM121
Slice Day 0
Slice Day 6
U3013MG
U3180MG
U3054MG
U3220MG
Core
Edge

Supplementary figure 2. Comparative representation of perivascular and diffuse invading tumor cells in different PDCX models. Representative PDCX slice images at day 0 and day 6 from tumor core and invading edge of indicated four different PDCX models compared to H&E and IHC of corresponding in vivo PDCX tumors. Scale bar 100 µm.

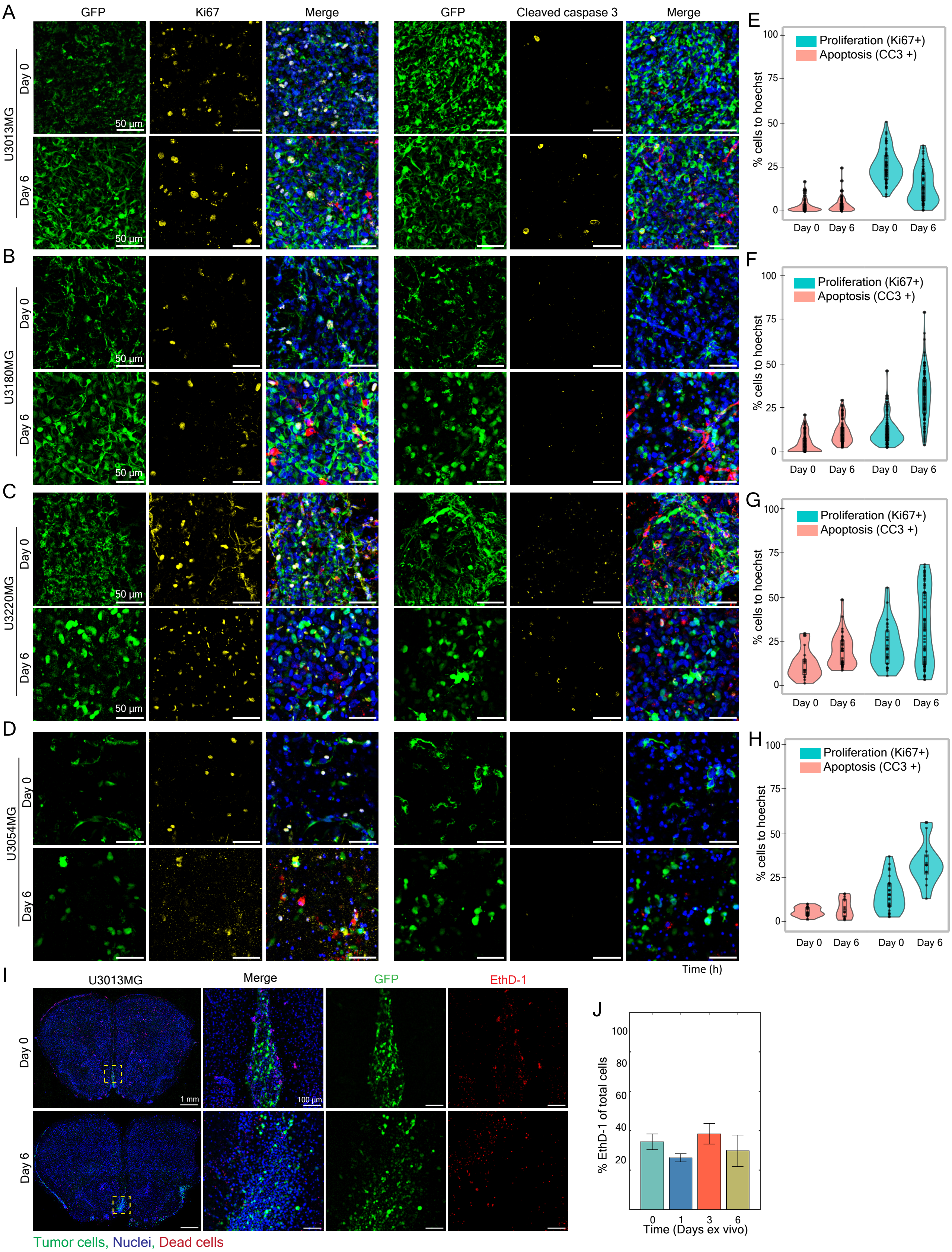

A
GFP
Ki67
Merge
GFP
Cleaved caspase 3
Merge
U3013MG
Day 0
Day 6
50 µm
B
U3180MG
C
U3220MG
D
U3054MG
E
F
G
H
Proliferation (Ki67+)
Apoptosis (CC3 +)
% cells to hoechst
100
75
50
25
0
Day 0
Day 6
Time (h)
I
U3013MG
Merge
GFP
EthD-1
1 mm
100 µm
Tumor cells, Nuclei, Dead cells
J
% EthD-1 of total cells
100
80
60
40
20
0
1
3
6
Time (Days ex vivo)

Supplementary figure 3. Programmed cell death and proliferation status of cells in slice culture. (A–D) Representative confocal images of U3013MG, U3180MG, U3220MG, and U3054MG PDCX slices immunostained at the end point of time-lapse imaging, showing cleaved caspase-3–positive or Ki67-positive cells. Scale bar: 50 µm. (E–H) Quantification of cleaved caspase-3–positive and Ki67-positive cells in PDCX slices (n = 6 slices per time point from 2 mouse brains for each PDCX model). (I) Representative confocal images stained with the dead-cell membrane-impermeable dye EthD-1 (red) and nuclear counterstain Hoechst (blue) at day 0 and after 6 days in glioblastoma PDCX brain slices in culture. Tumor cells are green. Scale bar: 1 mm (left column); 100 µm (zoomed panels). (J) Quantification of cell viability in ex vivo U3013MG brain slice culture (n = 12 slices from 2 mice). Bar height represents the mean; error bars represent the standard error of the mean. Violin plots show the kernel density estimate of the data distribution. Overlaid box plots indicate the median (center line) and the 25th-75th percentiles (bounds of box); whiskers extend to the largest/smallest value no further than 1.5× the interquartile range from the box; data points beyond this range are plotted individually as outliers. Individual data points are overlaid as dots.

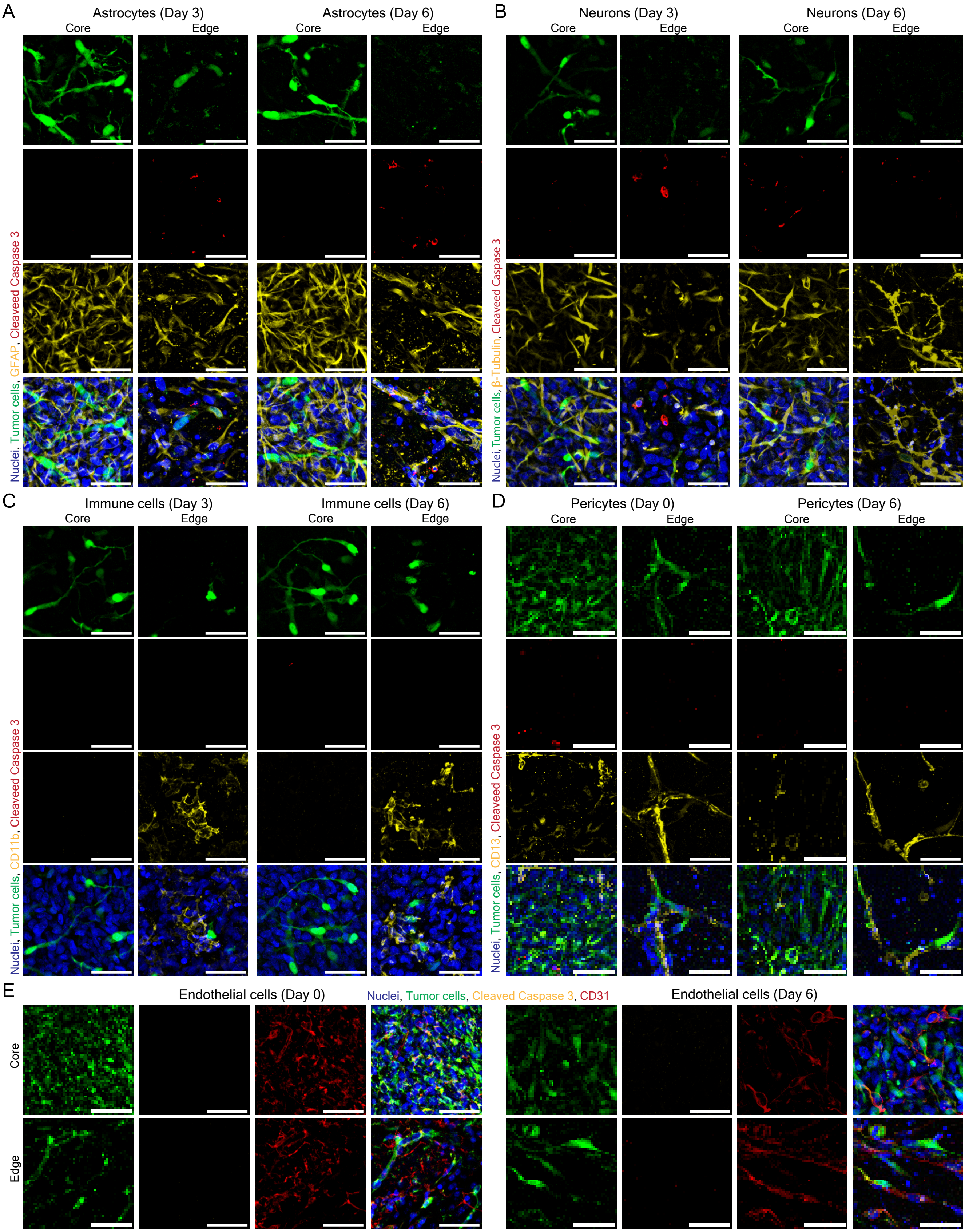
A
Astrocytes (Day 3)
Core
Edge
Astrocytes (Day 6)
Core
Edge
Nuclei, Tumor cells, GFAP, Cleaveed Caspase 3
B
Neurons (Day 3)
Core
Edge
Neurons (Day 6)
Core
Edge
Nuclei, Tumor cells, β-Tubulin, Cleaveed Caspase 3
C
Immune cells (Day 3)
Core
Edge
Immune cells (Day 6)
Core
Edge
Nuclei, Tumor cells, CD11b, Cleaveed Caspase 3
D
Pericytes (Day 0)
Core
Edge
Pericytes (Day 6)
Core
Edge
Nuclei, Tumor cells, CD13, Cleaveed Caspase 3
E
Endothelial cells (Day 0)
Nuclei, Tumor cells, Cleaved Caspase 3, CD31
Endothelial cells (Day 6)
Core
Edge

Supplementary figure 4. Tumor microenvironment health status in slice culture. (A–C) U3180MG slices immunostained at the end point of time-lapse imaging for GFAP (astrocytes), β-tubulin (neurons), or CD11b (immune cells), together with cleaved caspase-3. Representative images from tumor core and edge at day 3 and day 6 are shown. Scale bar: 50 μm. Tumor cells, green; cleaved caspase-3, red; indicated cell-type marker, yellow. (D, E) U3013MG slices immunostained at the end point of time-lapse imaging for CD13 (pericytes) or CD31 (endothelial cells), together with cleaved caspase-3. Representative images from tumor core and edge at day 0 and day 6 are shown. Scale bar: 50 μm. For the CD13 panel: Tumor cells, green; cleaved caspase-3, red; indicated cell-type marker, yellow. For the CD31 panel: Tumor cells, green; cleaved caspase-3, yellow; indicated cell-type marker, red.

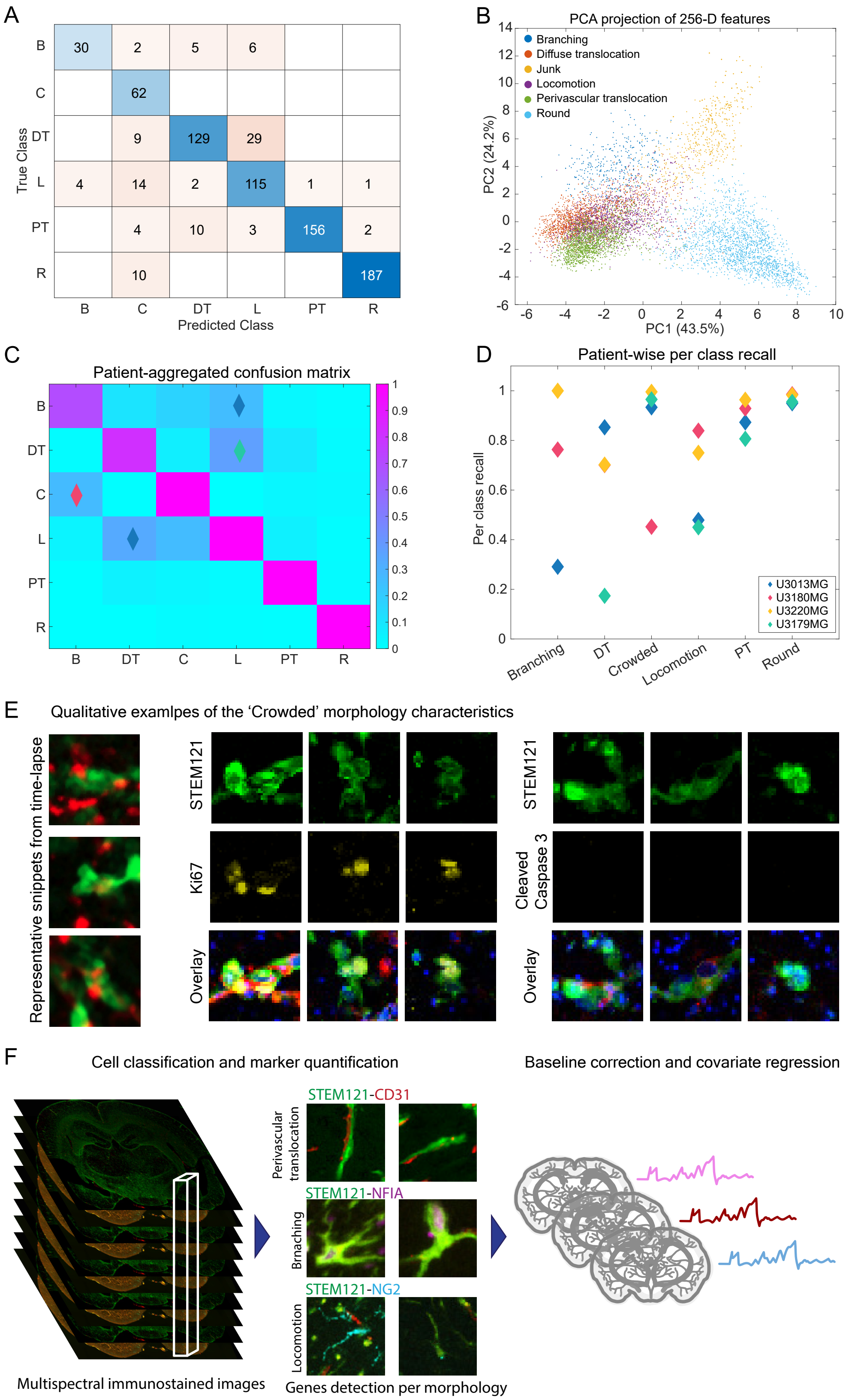

A
True Class
Predicted Class
B
C
DT
L
PT
R
B
PCA projection of 256-D features
Branching
Diffuse translocation
Junk
Locomotion
Perivascular translocation
Round
PC2 (24.2%)
PC1 (43.5%)
C
Patient-aggregated confusion matrix
D
Patient-wise per class recall
Per class recall
Branching
DT
Crowded
Locomotion
PT
Round
U3013MG
U3180MG
U3220MG
U3179MG
E
Qualitative examlpes of the 'Crowded' morphology characteristics
Representative snippets from time-lapse
STEM121
Ki67
Overlay
Cleaved Caspase 3
F
Cell classification and marker quantification
Baseline correction and covariate regression
STEM121-CD31
Perivascular translocation
STEM121-NFIA
Brnaching
STEM121-NG2
Locomotion
Multispectral immunostained images
Genes detection per morphology

Supplementary figure 5. Morphological classification performance and marker quantification workflow. (A) Confusion matrix of true versus predicted classes when evaluating the neural network on the held-out test set, stratified from the full training dataset. B, branching; DT, diffuse translocation; C, crowded; L, locomotion; PT, perivascular translocation; R, round. (B) PCA projection of the final 256-node ReLU layer showing morphological class distribution across the learned feature space (n = 16 slices from 16 mice). (C) Patient-aggregated confusion matrix for classification of morphological classes on a patient-wise held-out test set. Diamonds indicate frequently occurring misclassifications where one PDCX model dominated the signal (max2mean ratio > 2.5). (D) Scatter plot showing the patient-wise per-class recall. (E) Qualitative examples of crowded cell morphologies classified by GlioTrace. (F) Schematic of the analysis pipeline for marker quantification of multispectral immunofluorescence images.

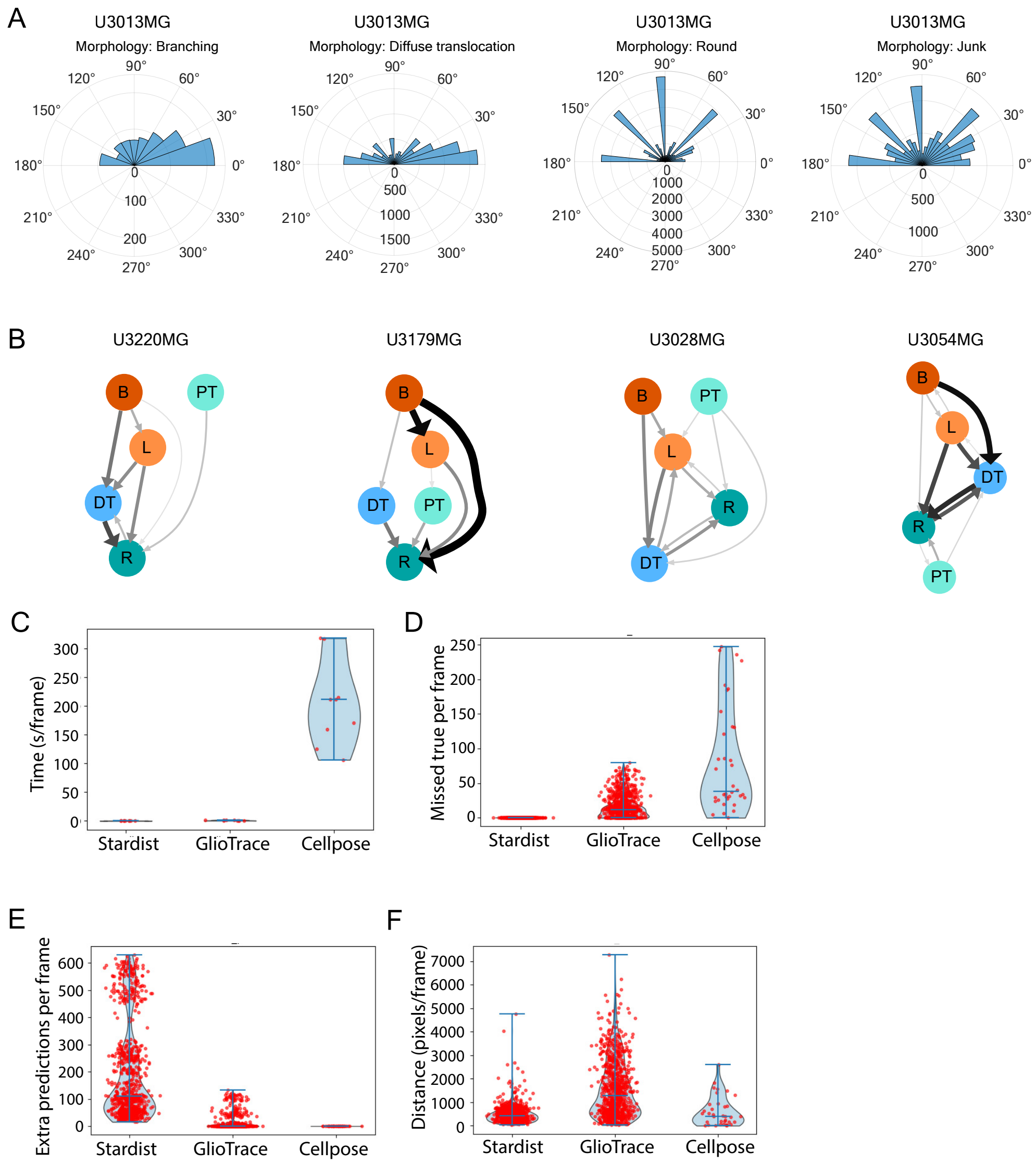
A
U3013MG
Morphology: Branching
U3013MG
Morphology: Diffuse translocation
U3013MG
Morphology: Round
U3013MG
Morphology: Junk
90°
60°
30°
0°
330°
300°
270°
240°
210°
180°
150°
120°
0
100
200
500
1000
1500
2000
3000
4000
5000
B
U3220MG
U3179MG
U3028MG
U3054MG
B
L
DT
R
PT
C
Time (s/frame)
300
250
200
150
100
50
0
Stardist
GlioTrace
Cellpose
D
Missed true per frame
250
200
150
100
50
0
Stardist
GlioTrace
Cellpose
E
Extra predictions per frame
600
500
400
300
200
100
0
Stardist
GlioTrace
Cellpose
F
Distance (pixels/frame)
7000
6000
5000
4000
3000
2000
1000
0
Stardist
GlioTrace
Cellpose

Supplementary figure 6. Demonstration of PDCX models recapitulating human glioblastoma phenotypes in slice culture. (A) Turning-angle distribution of migratory cells classified as branching, diffuse translocation, round, and crowded (cells with unidentifiable morphology) (n = 21 slices from 15 mice). (B) State-switching hierarchies displaying the transition rates between different morphological states of U3220MG, U3179MG, U3028MG, and U3054MG tumor cells. Thicker lines indicate more frequent transitions (n = 40 slices from 22 mice). (C–F) Benchmarking cell detection on a manually annotated in-house dataset comparing three different methods for cell detection: StarDist, GlioTrace, and Cellpose. (C) Processing time in seconds per frame for cell detection. (D) Missed true predictions per frame. (E) Extra predictions per frame. (F) Distance between true and predicted cell coordinates per frame. Violin plots show the kernel density estimate of the data distribution. The horizontal line indicates the median; the vertical line spans the minimum to maximum data values. Individual data points are overlaid as jittered dots.

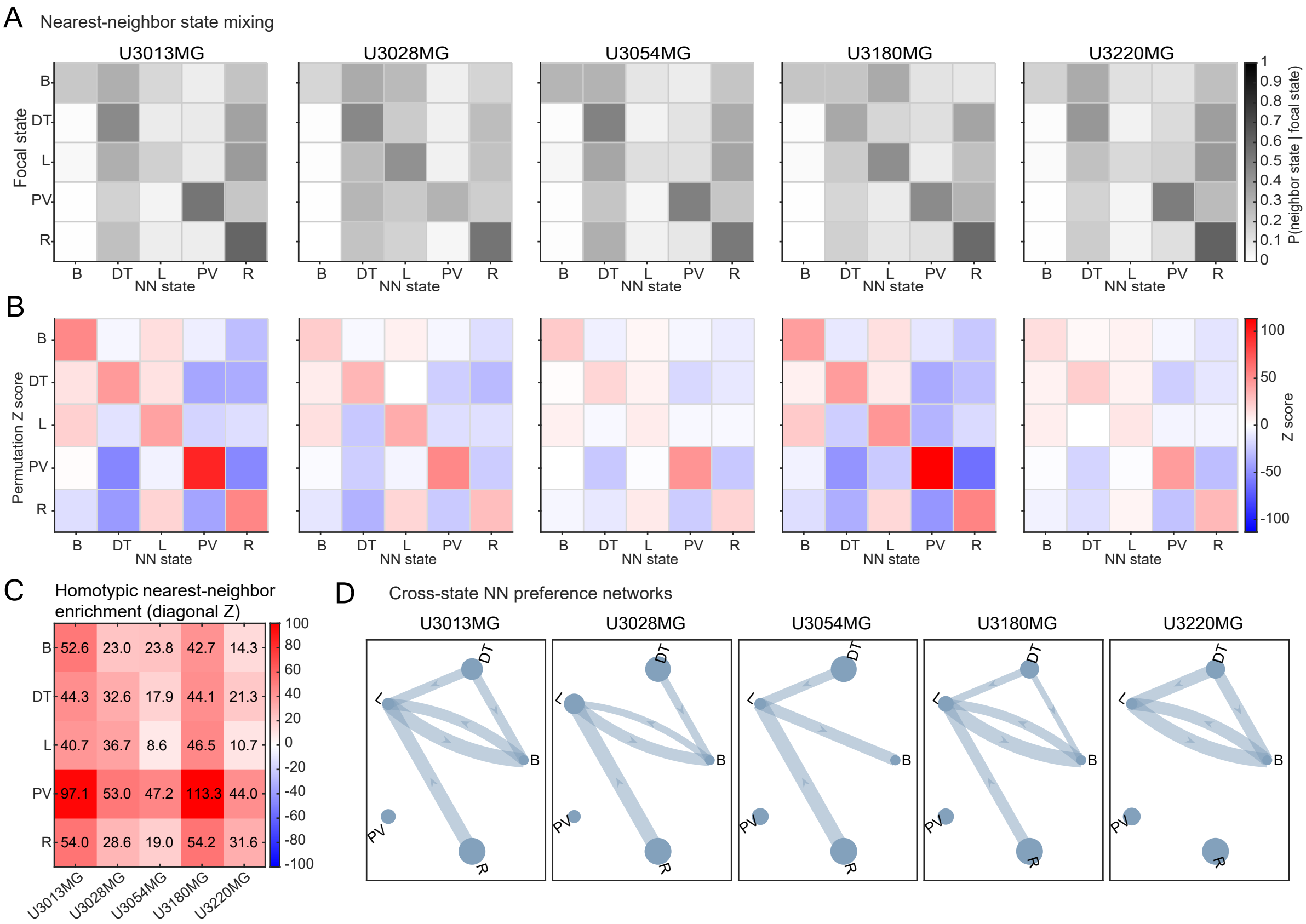
A
Nearest-neighbor state mixing
U3013MG
U3028MG
U3054MG
U3180MG
U3220MG
Focal state
B
DT
L
PV
R
NN state
P(neighbor state | focal state)
1
0.9
0.8
0.7
0.6
0.5
0.4
0.3
0.2
0.1
0
B
Permutation Z score
Z score
100
50
0
-50
-100
C
Homotypic nearest-neighbor enrichment (diagonal Z)
B 52.6 23.0 23.8 42.7 14.3
DT 44.3 32.6 17.9 44.1 21.3
L 40.7 36.7 8.6 46.5 10.7
PV 97.1 53.0 47.2 113.3 44.0
R 54.0 28.6 19.0 54.2 31.6
100
80
60
40
20
0
-20
-40
-60
-80
-100
D
Cross-state NN preference networks

Supplementary figure 7. Spatial correlation of morphologies. (A) State-neighbor probability matrices for five PDCX models. Each row is a state, showing the probabilities of having itself and each other state as the nearest neighbor. Values represent the conditional probability P (neighbor state = j | state = i). (B) Permutation Z-score derived by adjusting for the null-model probability of neighboring states. Positive Z-scores indicate enrichment of a given state–state neighbor pair relative to random mixing of state labels; negative values indicate a neighboring relationship less common than expected by chance. (C) Heatmap summarizing homotypic nearest-neighbor enrichment (diagonal Z-scores) across PDCX models, highlighting the degree to which cells of the same phenotypic state preferentially cluster. (D) Directed network representations of cross-state nearest-neighbor preferences. Nodes represent invasion phenotypes and edges indicate enriched nearest-neighbor associations between different states. Edge thickness reflects the strength of enrichment relative to the permutation-based null model. Abbreviations: B, branching; DT, diffuse translocation; L, locomotion; PT, perivascular translocation; R, round.

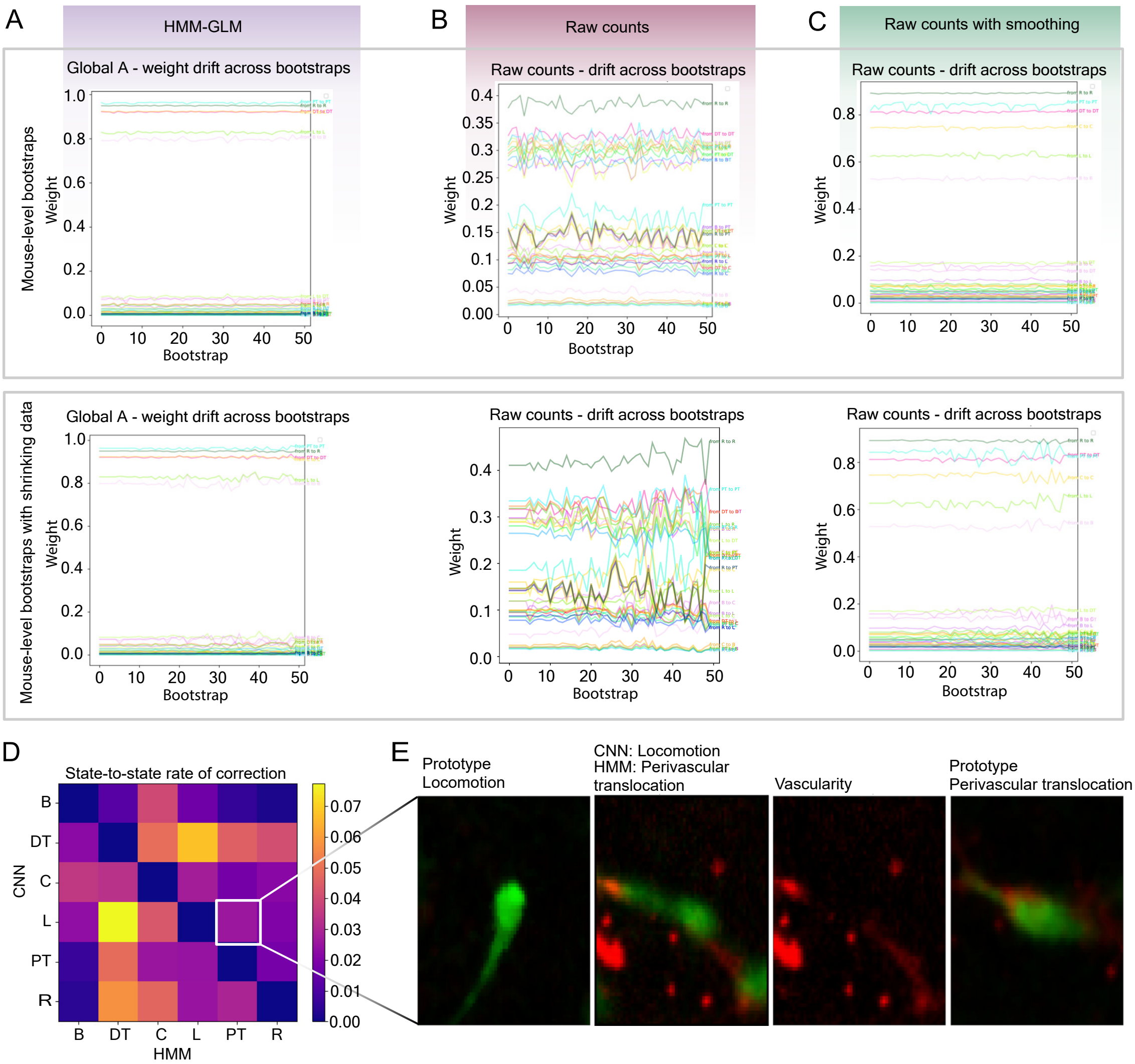

A
HMM-GLM
B
Raw counts
C
Raw counts with smoothing
Mouse-level bootstraps
Global A - weight drift across bootstraps
Raw counts - drift across bootstraps
Raw counts - drift across bootstraps
Weight
Bootstrap
Mouse-level bootstraps with shrinking data
Global A - weight drift across bootstraps
Raw counts - drift across bootstraps
Raw counts - drift across bootstraps
D
State-to-state rate of correction
CNN
HMM
B
DT
C
L
PT
R
E
Prototype
Locomotion
CNN: Locomotion
HMM: Perivascular
translocation
Vascularity
Prototype
Perivascular translocation

Supplementary figure 8. Transition weight bootstrapping: comparison of HMM-derived transition matrices, raw counts, and counts from smoothed state paths. (A–C) Line plots showing how each transition weight fluctuates across bootstraps when sampling on mouse level (top) or sampling on mouse level while shrinking the data (bottom). (A) Transition-weight fluctuations across bootstraps when fitting an HMM to the data and deriving transitions from the global transition matrix. (B) Transition-weight fluctuations when counting transitions from raw state paths. (C) Transition-weight fluctuations when counting transitions from smoothed state paths. (D) Contingency table showing the ratio of HMM-corrected observations (mean across bootstraps). (E) Example correction from locomotion (CNN class) to perivascular translocation (HMM class). Microscopy images show prototype locomotion and perivascular translocation cells, a green-red overlay of the observation, and the red channel alone to highlight the underlying vasculature. All tests were performed on the U3013MG control dataset (23 mice, 49 slices).

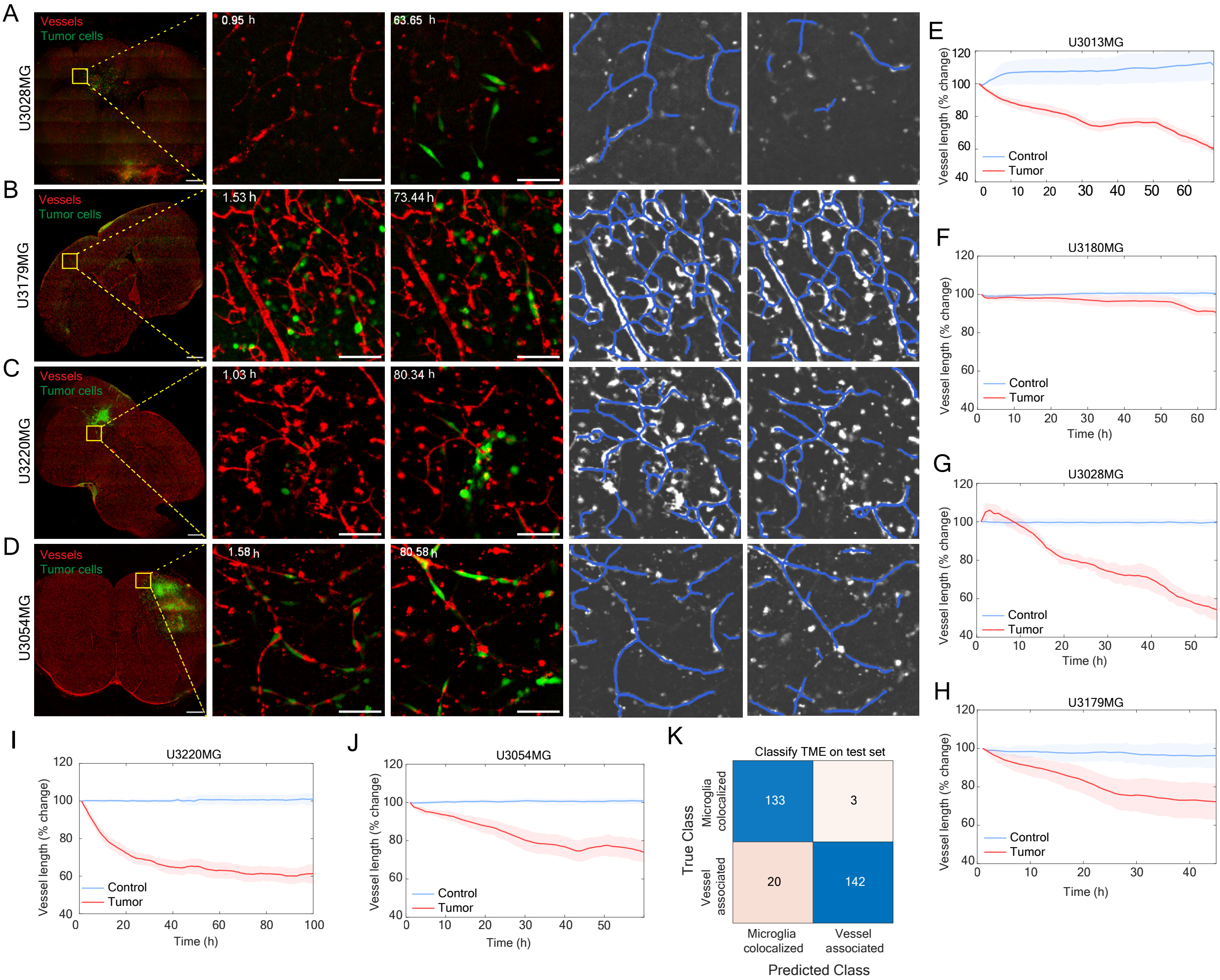

A
Vessels
Tumor cells
U3028MG
0.95 h
63.65 h
B
Vessels
Tumor cells
U3179MG
1.53 h
73.44 h
C
Vessels
Tumor cells
U3220MG
1.03 h
80.34 h
D
Vessels
Tumor cells
U3054MG
1.58 h
80.58 h
E
U3013MG
Vessel length (% change)
Control
Tumor
F
U3180MG
Vessel length (% change)
Time (h)
Control
Tumor
G
U3028MG
Vessel length (% change)
Time (h)
Control
Tumor
H
U3179MG
Vessel length (% change)
Time (h)
Control
Tumor
I
U3220MG
Vessel length (% change)
Time (h)
Control
Tumor
J
U3054MG
Vessel length (% change)
Time (h)
Control
Tumor
K
Classify TME on test set
True Class
Microglia colocalized
Vessel associated
133
3
20
142
Microglia colocalized
Vessel associated
Predicted Class

Supplementary figure 9. Patient-specific tumor–vessel interactions in slice culture. (A–D) Confocal whole-brain slice images at the starting point (left) with zoomed-in snippets at 0 h and the end point of time-lapse imaging, highlighting structural changes in tumor-associated vasculature in proneural (U3028MG), classical (U3179MG), and mesenchymal (U3220MG, U3054MG) glioblastoma tumors. Scale bars: 1 mm (left), 100 μm (zoomed). (E–J) Comparative measurement of relative vessel length at the tumor-progressing edge and normal brain area from the same slices in U3013MG, U3180MG, U3028MG, U3179MG, U3220MG, and U3054MG slices (n = 60 slices from 34 mice). Data are presented as mean ± SEM. (bold lines and shaded bands respectively). (K) Confusion matrix of the neural network classifying interactions between tumor cells and the microenvironment.

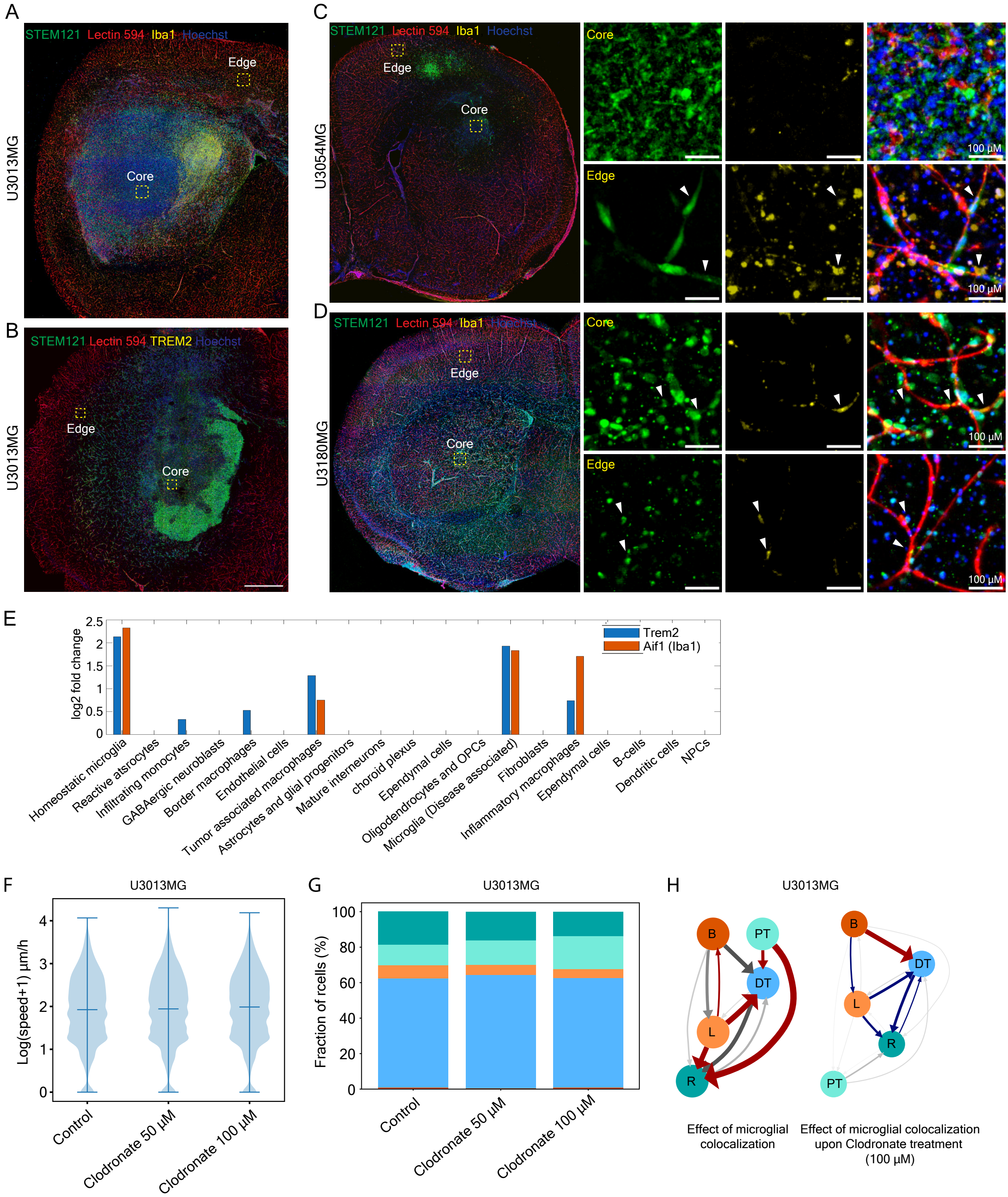

A
STEM121 Lectin 594 Iba1 Hoechst
U3013MG
Edge
Core
B
STEM121 Lectin 594 TREM2 Hoechst
U3013MG
Edge
Core
C
STEM121 Lectin 594 Iba1 Hoechst
U3054MG
Edge
Core
100 µM
D
STEM121 Lectin 594 Iba1 Hoechst
U3180MG
Edge
Core
100 µM
E
log2 fold change
Trem2
Aif1 (Iba1)
Homeostatic microglia
Reactive atsrocytes
Infiltrating monocytes
GABAergic neuroblasts
Border macrophages
Endothelial cells
Tumor associated macrophages
Astrocytes and glial progenitors
Mature interneurons
choroid plexus
Ependymal cells
Oligodendrocytes and OPCs
Microglia (Disease associated)
Fibroblasts
Inflammatory macrophages
Ependymal cells
B-cells
Dendritic cells
NPCs
F
U3013MG
Log(speed+1) µm/h
Control
Clodronate 50 µM
Clodronate 100 µM
G
U3013MG
Fraction of rcells (%)
Control
Clodronate 50 µM
Clodronate 100 µM
H
U3013MG
B
PT
DT
L
R
Effect of microglial colocalization
Effect of microglial colocalization upon Clodronate treatment (100 µM)

Supplementary figure 10. Glioblastoma tumor cells activate microglia at the tumor-progressing edge in perivascularly invading solid tumors. (A–B) Whole-tumor images of U3013MG slices showing core versus edge after immunostaining for Iba1-positive and TREM2-positive microglia, referenced to Fig. 5H–I. (C–D) Representative slices of U3054MG and U3180MG stained with the human cell marker STEM121 (green), vessels (lectin 594, red), and the microglia activation marker Iba1 (yellow) at the imaging end point. Representative core and edge sites are indicated by dashed squares for zoomed-in snippets. Arrowheads indicate tumor cells interacting with activated microglia at the same location. Scale bar: 100 µm (zoomed). (E) Average log2 fold change of Trem2 and Iba1 across single cells in all mouse clusters. Marker fold change is computed by comparing expression in each cluster one-versus-all. (F) Quantification of migration speed in U3013MG slices treated with clodronate. Speed is log(x + 1) (n = 16 slices from 2 mice). Violin plots show the kernel density estimate of the data distribution. The horizontal line indicates the median; the vertical line spans the minimum to maximum data values. (G) Distribution of morphological states in U3013MG slices treated with or without clodronate (n = 16 slices from 2 mice). (H) Differential transition networks showing the effect of microglial colocalization on state transitions in untreated (left) or clodronate-treated (right) U3013MG cells. Red edges indicate increased transition rates upon colocalization, whereas blue edges indicate decreased transitions.

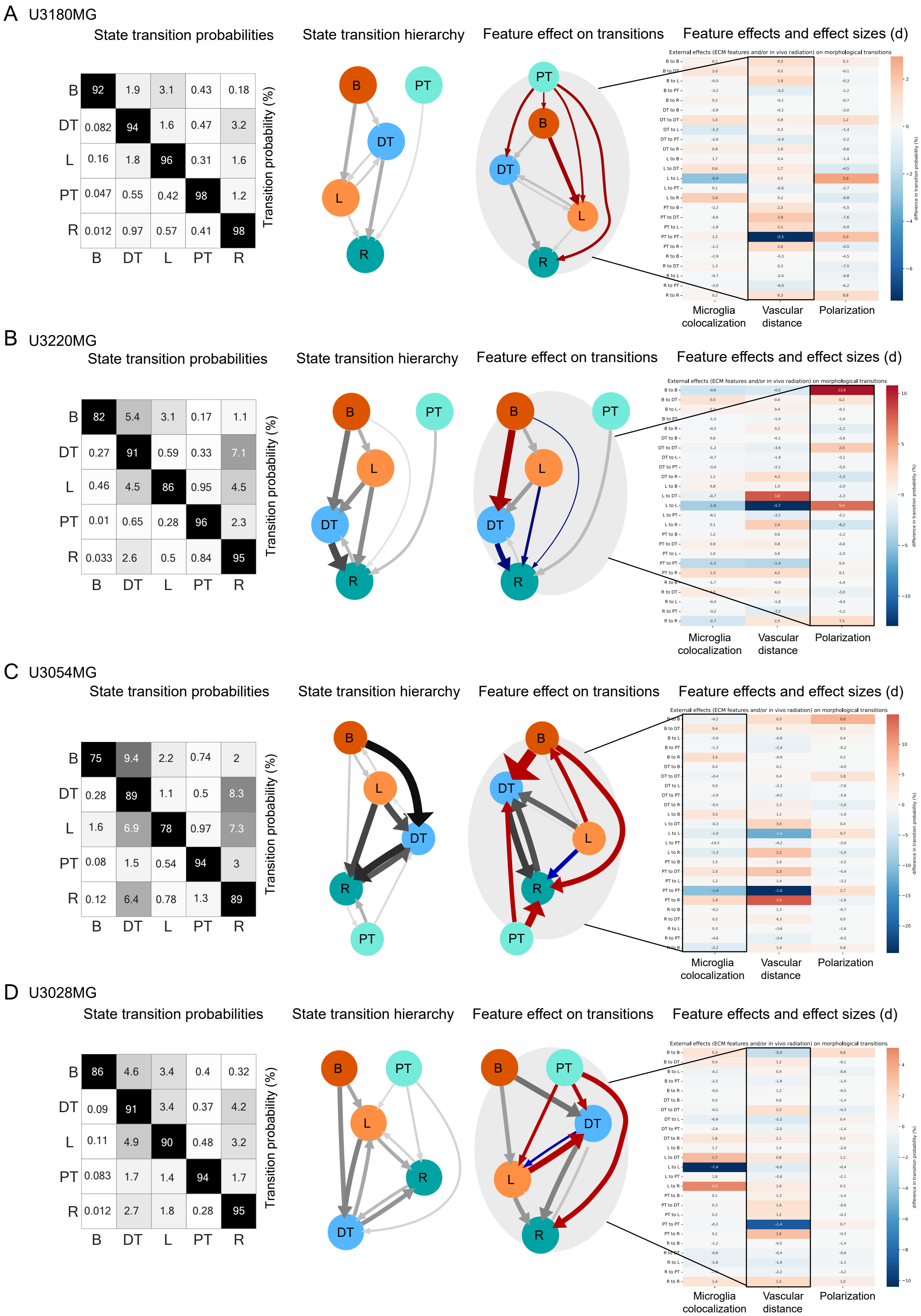
A U3180MG
State transition probabilities
State transition hierarchy
Feature effect on transitions
Feature effects and effect sizes (d)
B 92 1.9 3.1 0.43 0.18
DT 0.082 94 1.6 0.47 3.2
L 0.16 1.8 96 0.31 1.6
PT 0.047 0.55 0.42 98 1.2
R 0.012 0.97 0.57 0.41 98
B DT L PT R
Transition probability (%)
Microglia colocalization
Vascular distance
Polarization
B U3220MG
State transition probabilities
State transition hierarchy
Feature effect on transitions
Feature effects and effect sizes (d)
B 82 5.4 3.1 0.17 1.1
DT 0.27 91 0.59 0.33 7.1
L 0.46 4.5 86 0.95 4.5
PT 0.01 0.65 0.28 96 2.3
R 0.033 2.6 0.5 0.84 95
B DT L PT R
Transition probability (%)
Microglia colocalization
Vascular distance
Polarization
C U3054MG
State transition probabilities
State transition hierarchy
Feature effect on transitions
Feature effects and effect sizes (d)
B 75 9.4 2.2 0.74 2
DT 0.28 89 1.1 0.5 8.3
L 1.6 6.9 78 0.97 7.3
PT 0.08 1.5 0.54 94 3
R 0.12 6.4 0.78 1.3 89
B DT L PT R
Transition probability (%)
Microglia colocalization
Vascular distance
Polarization
D U3028MG
State transition probabilities
State transition hierarchy
Feature effect on transitions
Feature effects and effect sizes (d)
B 86 4.6 3.4 0.4 0.32
DT 0.09 91 3.4 0.37 4.2
L 0.11 4.9 90 0.48 3.2
PT 0.083 1.7 1.4 94 1.7
R 0.012 2.7 1.8 0.28 95
B DT L PT R
Transition probability (%)
Microglia colocalization
Vascular distance
Polarization

Supplementary figure 11. Microenvironmental effects on morphological state transitions. (A–D) Left: state-transition probability matrix shaded by probability. Darker shading indicates a higher transition rate. The diagonal represents self-transitions back to the same state. Middle left: state-transition hierarchy derived from the state-transition matrix. Edge thickness represents transition probability. Self-transitions are excluded. Middle right: differential transition hierarchy showing the effect of microenvironmental features on transitions. Color indicates the sign of the transition difference when comparing transition frequency in the presence versus absence, or high versus low value, of the feature; red indicates upregulation upon feature presence, and blue indicates downregulation. Right: heatmap of differences in transition probabilities. Each row is a transition from state i to state j. Each column is a microenvironmental feature. Colors indicate up- or downregulation of transition probability. Numbers are Cohen's d effect sizes.

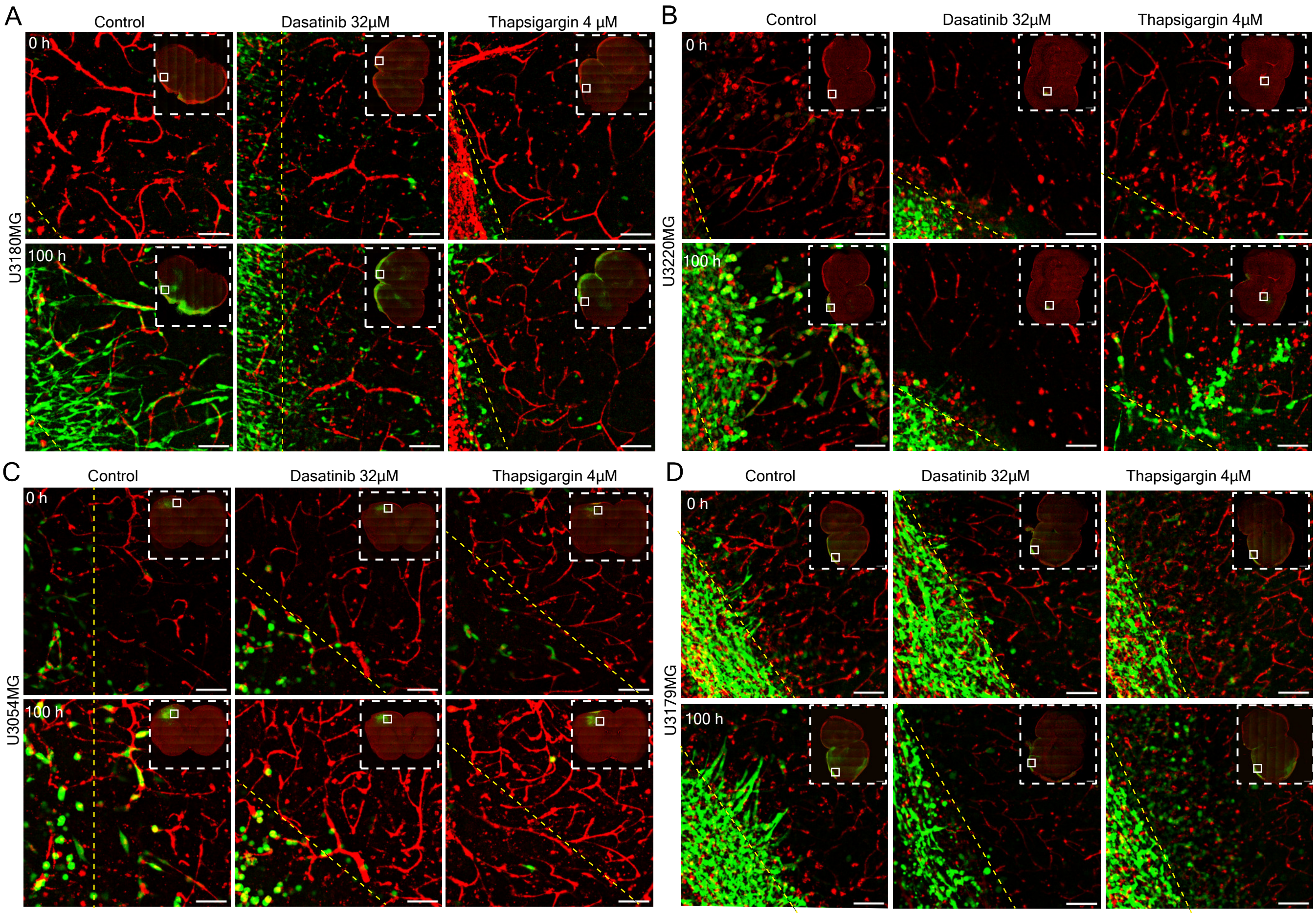
A
Control
Dasatinib 32μM
Thapsigargin 4 μM
0 h
100 h
U3180MG
B
Control
Dasatinib 32μM
Thapsigargin 4μM
0 h
100 h
U3220MG
C
Control
Dasatinib 32μM
Thapsigargin 4μM
0 h
100 h
U3054MG
D
Control
Dasatinib 32μM
Thapsigargin 4μM
0 h
100 h
U3179MG

Supplementary figure 12. Treatment effects of dasatinib and thapsigargin on tumor cell migration and invasion in PDCX slices. (A–D) Representative first and last time-point images of U3180MG, U3220MG, U3054MG, and U3179MG slice cultures untreated or treated with dasatinib (32 μM) or thapsigargin (4 μM). Vessels, red; tumor cells, green. Dotted line represents the tumor border at the start of time-lapse imaging. Scale bar: 100 μm.

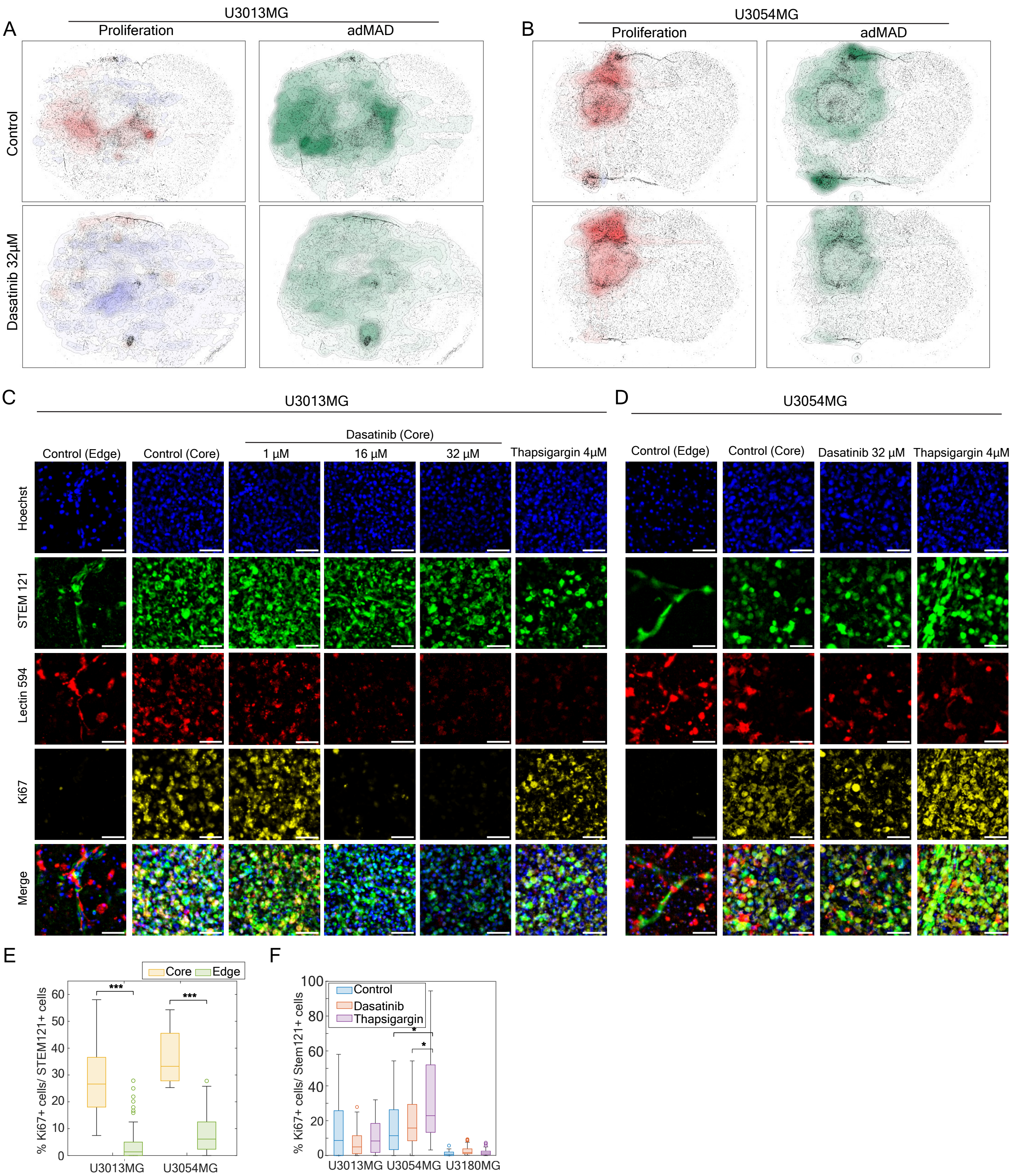

A
U3013MG
Proliferation
adMAD
Control
Dasatinib 32µM
B
U3054MG
Proliferation
adMAD
C
U3013MG
Dasatinib (Core)
Control (Edge)
Control (Core)
1 µM
16 µM
32 µM
Thapsigargin 4µM
Hoechst
STEM 121
Lectin 594
Ki67
Merge
D
U3054MG
Control (Edge)
Control (Core)
Dasatinib 32 µM
Thapsigargin 4µM
E
Core
Edge
% Ki67+ cells/ STEM121+ cells
U3013MG
U3054MG
F
Control
Dasatinib
Thapsigargin
% Ki67+ cells/ Stem121+ cells
U3013MG
U3054MG
U3180MG

Supplementary figure 13. Spatial analysis and post-time-lapse immunostaining reveal differential drug effects in tumor core and invasive front. (A–B) Spatial maps of tumor growth and migration in whole-brain slices, illustrating differential drug responses in the tumor core and invasive front. Blue, reduced growth; red, increased growth; green, migration extent. (C) Representative snippets from the tumor core of U3013MG slices showing the dose-dependent effect of dasatinib on cell proliferation and relative resistance to thapsigargin. Scale bar: 100 µm. (D) Representative images from the tumor core of U3054MG slices demonstrating high Ki67 signals and relative resistance to thapsigargin. (E) Percentage of Ki67-positive proliferating cells normalized to STEM121-positive tumor cells, comparing core and edge regions in U3013MG and U3054MG (n = 6 slices from 6 mice). Statistical comparisons between groups were performed using Welch's two-sample t-test (two-tailed). Results are reported as t(df), p, Hedges' g, and 95% CI. Effect sizes were calculated as Hedges' g using the pooled standard deviation and a small-sample correction, with 95% confidence intervals estimated from the standard error of Hedges' g. U3013MG: $t(67.04) = 12.000$, $p = 2.36e-18$, Hedges' $g = 2.423$, 95% CI [1.952, 2.894]. U3054MG: $t(18.32) = 9.091$, $p = 3.268e-08$, Hedges' $g = 3.257$, 95% CI [2.333, 4.181]. (F) Ki67-positive tumor cells in dasatinib- and thapsigargin-treated slices, normalized to control (n = 18 slices from 7 mice). Box plots show the median (centre line) and 25th–75th percentiles (bounds of box); whiskers extend to the most extreme data points not considered outliers (within 1.5× the interquartile range); outliers are plotted individually. Differences among groups were assessed separately for each dataset using one-way ANOVA. Post-hoc pairwise comparisons were performed using Tukey's HSD. Results are reported as F(df1, df2), p, eta squared and 95% CI. For U3054MG, $F(2,118)=8.37$, $p=3.99\times10^{-4}$, $\eta^2=0.12$, 95% CI [0.03, 0.23]. Significance is indicated by asterisks. * = $p \leq 0.05$, ** = $p \leq 0.01$, *** = $p \leq 0.001$.

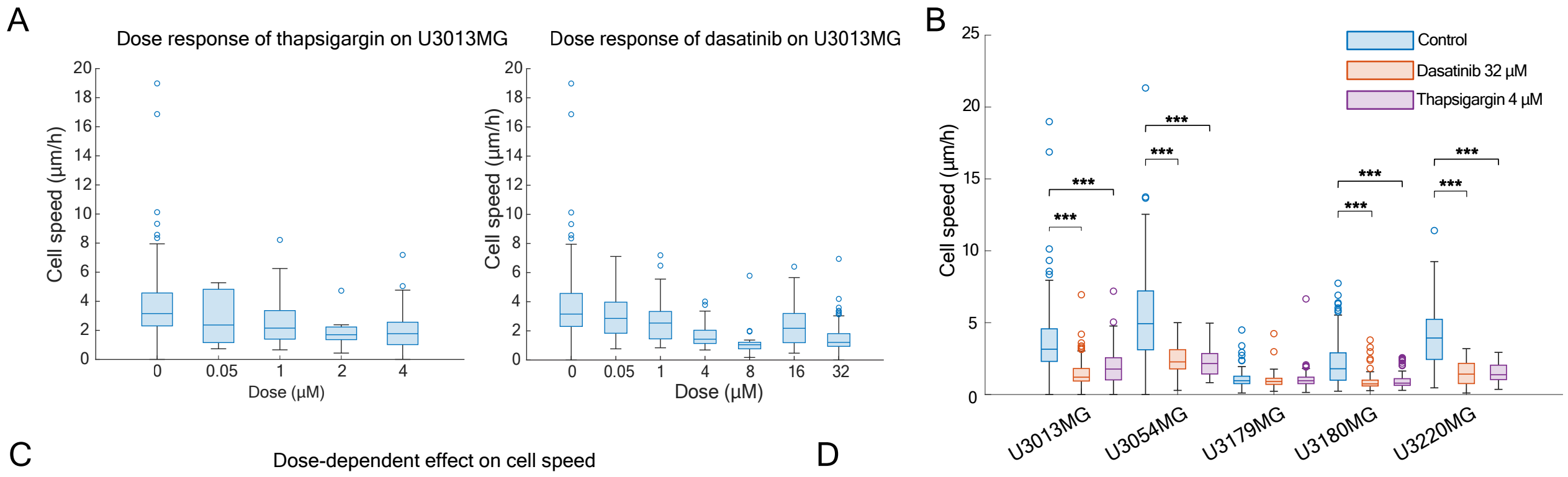

C

Dose-dependent effect on cell speed

HGCC	Treatment	Estimate	SE	tStat	p-Value
U3013MG	Dasatinib	-0.069	0.005	-13.75	1.23e-40
	Thapsigargin	-0.423	0.049	-8.79	3.88e-18
U3054MG	Dasatinib	-0.121	0.01	-11.61	2.23e-26
	Thapsigargin	-1.015	0.089	-11.43	9.75e-26
U3179MG	Dasatinib	-0.006	0.003	-2.08	0.039
	Thapsigargin	-0.002	0.022	-0.11	0.914
U3180MG	Dasatinib	-0.019	0.004	-4.82	1.69e-6
	Thapsigargin	-0.115	0.031	-3.65	2.8e-4
U3220MG	Dasatinib	-0.06	0.011	-5.36	2.17e-7
	Thapsigargin	-0.52	0.1	-5.18	5.13e-7

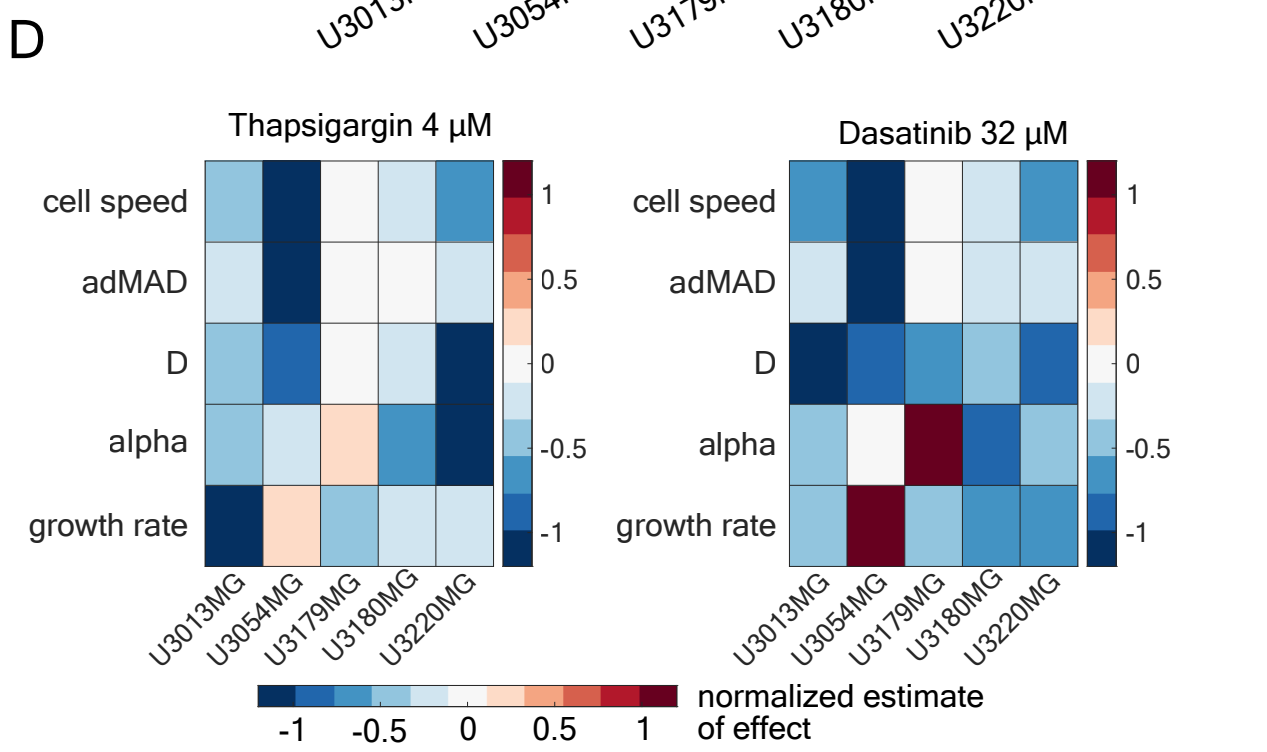

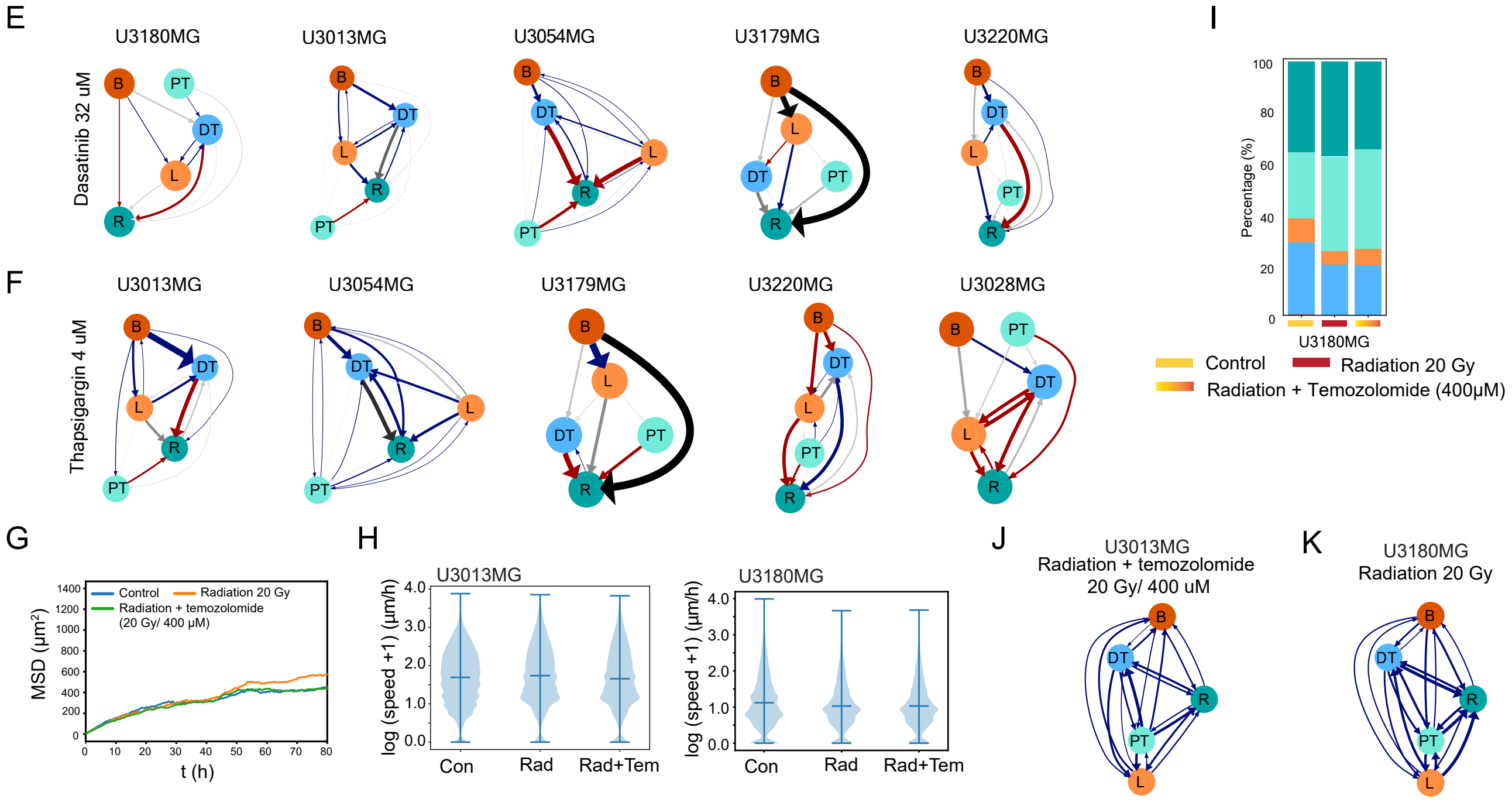

Supplementary figure 14. Treatment effects of dasatinib and thapsigargin on tumor cell migration and invasion in PDCX slices. (A) Quantification of the dose-dependent effects of dasatinib and thapsigargin on cell velocity in U3013MG slices (n = 49 slices from 15 mice). (B) Box plot of cell speed across treatment conditions and PDCX models (n = 102 slices from 33 mice). Differences among groups were assessed separately for each cell line using one-way ANOVA. Post-hoc pairwise comparisons were performed using Tukey's HSD. Results are reported as F(df1, df2), p, eta squared and 95% CI. For U3013MG, $F(2,749)=125.009$, $p = 1.42e-47$, $\eta^2=0.250$, 95% CI [0.199, 0.299]; for U3054MG, $F(2,281)=60.264$, $p = 1.66e-22$, $\eta^2=0.300$, 95% CI [0.213, 0.376]; for U3179MG, $F(2,276)=1.453$, $p=0.236$, $\eta^2=0.010$, 95% CI [0.000, 0.041]; for U3180MG, $F(2,514)=69.691$, $p = 1.658e-27$, $\eta^2=0.213$, 95% CI [0.153, 0.271]; and for U3220MG, $F(2,177)=37.880$, $p = 2.02e-14$, $\eta^2=0.300$, 95% CI [0.189, 0.393]. (C) Summary of dose-dependent effects of dasatinib and thapsigargin on cell speed across PDCX models, estimated using a linear mixed-effects model (LME). (D) Heatmaps showing drug effects on five measures of movement and growth across PDCX models. Values are normalized LME estimates. (E–F) Differential state-switching hierarchies of indicated PDCX models treated with (E) dasatinib and (F) thapsigargin. Red and blue edges indicate upregulation and downregulation, respectively, of transition rates relative to control. Edge thickness represents relative transition frequency. (G) MSD curves of U3180MG cells comparing control, in vivo–irradiated cells, and in vivo–irradiated plus temozolomide-treated cells. (H) Violin plots showing cell speed for treated versus untreated cells in U3013MG and U3180MG models, respectively. Speed is log(speed + 1) μm/h. (I) Bar plot showing the distribution of morphologies across treated and untreated cells. (J–K) Differential transition hierarchies showing the effect of treatment on transition probabilities. Edge thickness corresponds to transition probability. Box plots show the median (centre line) and 25th–75th percentiles (bounds of box); whiskers extend to the most extreme data points not considered outliers (within 1.5× the interquartile range); outliers are plotted individually. Violin plots show the kernel density estimate of the data distribution. The horizontal line indicates the median; the vertical line spans the minimum to maximum data values. n = 3 biologically independent samples per group. Significant Tukey-adjusted pairwise comparisons are indicated by asterisks. * = p ≤0.05, ** = p ≤0.01, *** = p ≤0.001.

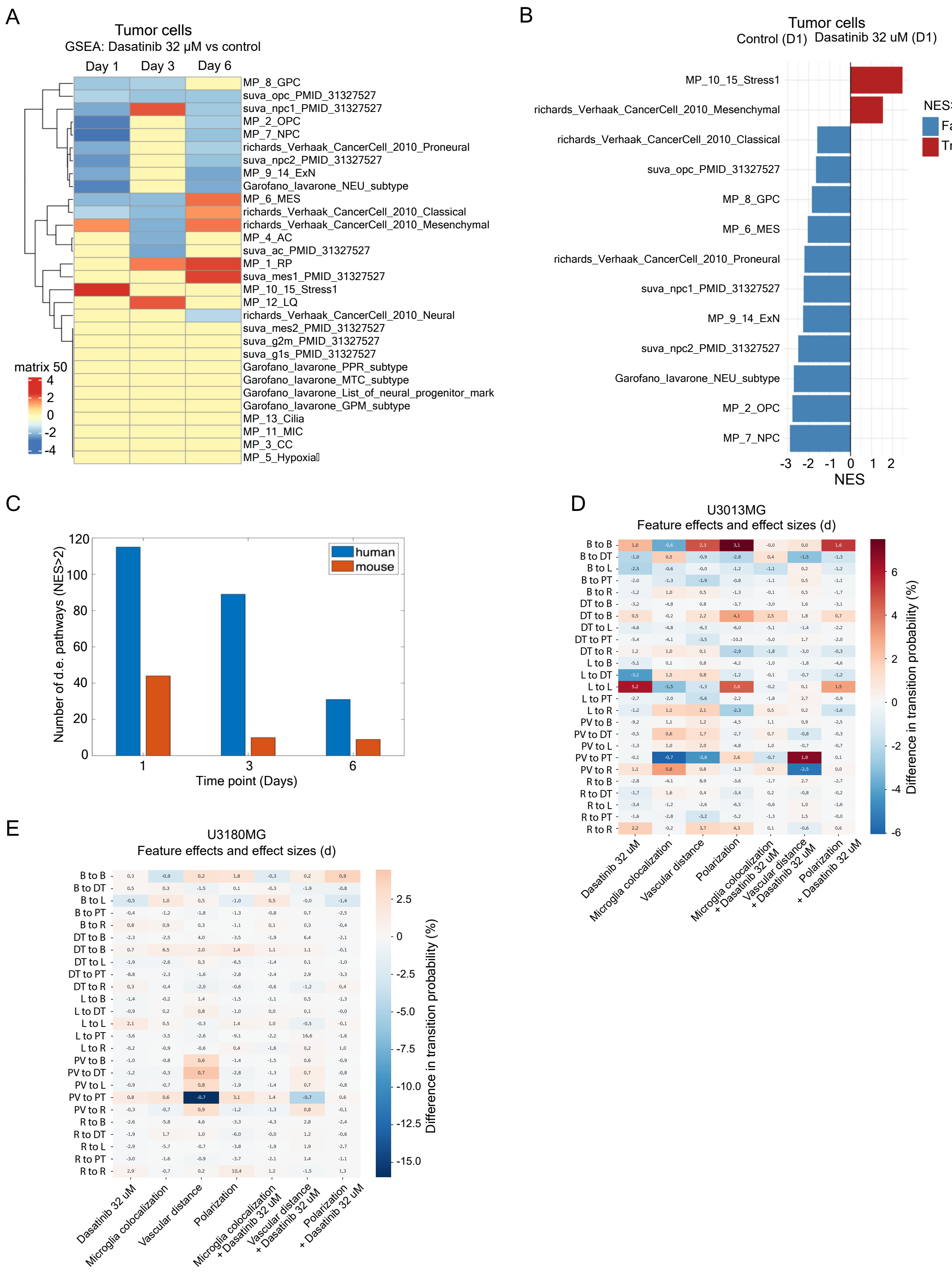
A
Tumor cells
GSEA: Dasatinib 32 μM vs control
Day 1
Day 3
Day 6
MP_8_GPC
suva_opc_PMID_31327527
suva_npc1_PMID_31327527
MP_2_OPC
MP_7_NPC
richards_Verhaak_CancerCell_2010_Proneural
suva_npc2_PMID_31327527
MP_9_14_ExN
Garofano_lavarone_NEU_subtype
MP_6_MES
richards_Verhaak_CancerCell_2010_Classical
richards_Verhaak_CancerCell_2010_Mesenchymal
MP_4_AC
suva_ac_PMID_31327527
MP_1_RP
suva_mes1_PMID_31327527
MP_10_15_Stress1
MP_12_LQ
richards_Verhaak_CancerCell_2010_Neural
suva_mes2_PMID_31327527
suva_g2m_PMID_31327527
suva_g1s_PMID_31327527
Garofano_lavarone_PPR_subtype
Garofano_lavarone_MTC_subtype
Garofano_lavarone_List_of_neural_progenitor_mark
Garofano_lavarone_GPM_subtype
MP_13_Cilia
MP_11_MIC
MP_3_CC
MP_5_Hypoxia
matrix 50
4
2
0
-2
-4
B
Tumor cells
Control (D1)
Dasatinib 32 uM (D1)
MP_10_15_Stress1
richards_Verhaak_CancerCell_2010_Mesenchymal
richards_Verhaak_CancerCell_2010_Classical
suva_opc_PMID_31327527
MP_8_GPC
MP_6_MES
richards_Verhaak_CancerCell_2010_Proneural
suva_npc1_PMID_31327527
MP_9_14_ExN
suva_npc2_PMID_31327527
Garofano_lavarone_NEU_subtype
MP_2_OPC
MP_7_NPC
NES>0
False
True
-3 -2 -1 0 1 2
NES
C
Number of d.e. pathways (NES>2)
120
100
80
60
40
20
0
human
mouse
1
3
6
Time point (Days)
D
U3013MG
Feature effects and effect sizes (d)
B to B
B to DT
B to L
B to PT
B to R
DT to B
DT to B
DT to L
DT to PT
DT to R
L to B
L to DT
L to L
L to PT
L to R
PV to B
PV to DT
PV to L
PV to PT
PV to R
R to B
R to DT
R to L
R to PT
R to R
Dasatinib 32 uM
Microglia colocalization
Vascular distance
Polarization
Microglia colocalization + Dasatinib 32 uM
Vascular distance + Dasatinib 32 uM
Polarization + Dasatinib 32 uM
Difference in transition probability (%)
6
4
2
0
-2
-4
-6
E
U3180MG
Feature effects and effect sizes (d)
B to B
B to DT
B to L
B to PT
B to R
DT to B
DT to B
DT to L
DT to PT
DT to R
L to B
L to DT
L to L
L to PT
L to R
PV to B
PV to DT
PV to L
PV to PT
PV to R
R to B
R to DT
R to L
R to PT
R to R
Dasatinib 32 uM
Microglia colocalization
Vascular distance
Polarization
Microglia colocalization + Dasatinib 32 uM
Vascular distance + Dasatinib 32 uM
Polarization + Dasatinib 32 uM
Difference in transition probability (%)
2.5
0
-2.5
-5.0
-7.5
-10.0
-12.5
-15.0

Supplementary figure 15. Transcriptomics of treated slices. (A) Heatmap of GSEA analysis showing up- or downregulation of published GBM gene-set signatures, comparing treated and untreated slices at each time point. (B) Horizontal bar plot of NES scores for up- and downregulated programs comparing treated and untreated slices at day 1. Red denotes upregulation in treated slices; blue denotes upregulation in control slices. (C) Bar plot of the number of differentially expressed programs with an NES score > 2, comparing human and mouse cells at each time point. (D–E) Heatmaps for U3013MG and U3180MG, respectively, showing the effects of dasatinib and/or environmental features on state transitions. Each row is a state transition, and each column is a covariate that can affect transitions, modeled by the HMM. Red/blue denotes increased/decreased transitions upon treatment and/or presence of the environmental feature. Numbers are Cohen's d.

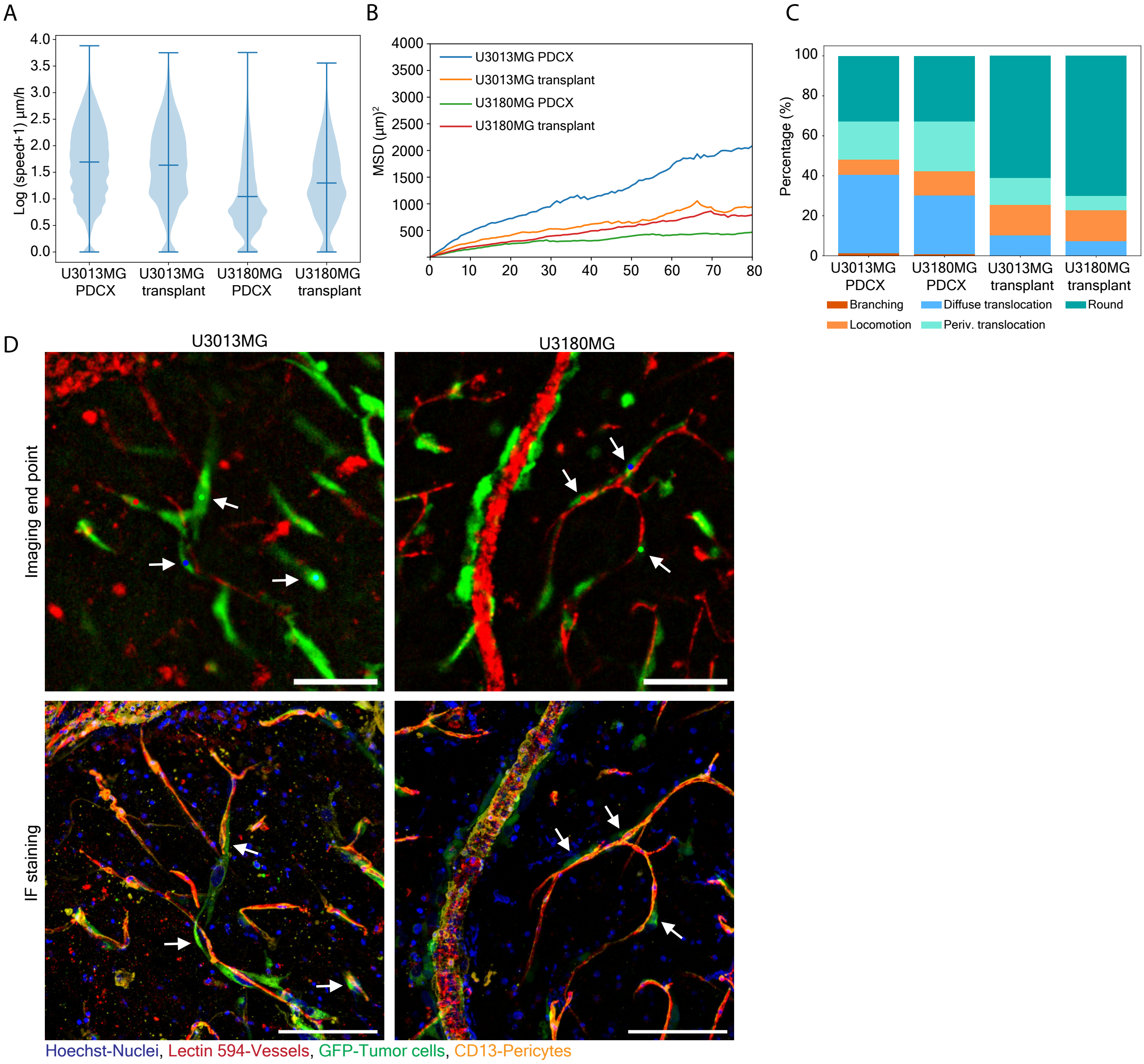
A
Log (speed+1) µm/h
4.0
3.5
3.0
2.5
2.0
1.5
1.0
0.5
0.0
U3013MG PDCX
U3013MG transplant
U3180MG PDCX
U3180MG transplant
B
MSD (µm)²
4000
3500
3000
2500
2000
1500
1000
500
0
10
20
30
40
50
60
70
80
U3013MG PDCX
U3013MG transplant
U3180MG PDCX
U3180MG transplant
C
Percentage (%)
100
80
60
40
20
0
U3013MG PDCX
U3180MG PDCX
U3013MG transplant
U3180MG transplant
Branching
Diffuse translocation
Round
Locomotion
Periv. translocation
D
U3013MG
U3180MG
Imaging end point
IF staining
Hoechst-Nuclei, Lectin 594-Vessels, GFP-Tumor cells, CD13-Pericytes

Supplementary figure 16. Comparison of invasion phenotypes in PDCX-derived tumors and directly transplanted slice cultures. (A) Quantification of migration speed across the two preparation methods, indicating lower average migratory activity in directly transplanted slices. (n=46 slices from n=8 mice). (B) Mean squared displacement (MSD) analysis of tumor cells in PDCX-derived models versus direct transplantation (n=46 slices from n=8 mice). (C) Distribution of morphological invasion phenotypes detected by the GlioTrace classifier in PDCX-derived slices and directly transplanted slice cultures. Directly transplanted slices showed a higher proportion of round cells and reduced representation of invasive morphologies relative to PDCX-derived tumors. (n = 46 slices from 8 mice). (D) Representative example of tumor cells interacting with CD13-positive pericytes and vessels, tracked back to time-lapse imaging. Scale bar: 100 µm. Violin plots show the kernel density estimate of the data distribution. The horizontal line indicates the median; the vertical line spans the minimum to maximum data values. Individual data points are overlaid as jittered dots (red). n = 3 biologically independent samples per group.
