# Supplementary material for: Reconstructing the single-cell spatiotemporal dynamics of glioblastoma invasion
Source: Nat Commun. 2026 Nov 2;17:11245. doi: 10.1038/s41467-026-78908-9 (PMC13728213; doi:10.1038/s41467-026-78908-9)
Supplement: Supplementary file 4 [file 41467_2026_78908_MOESM2_ESM.pdf]

Description of Additional Supplementary Files

File name: Supplementary Dataset 1
Description: Sheet 1, experimental and image-acquisition information for each slice from each mouse brain for all PDCX models. Sheet 2, clinical information for the human glioblastoma patient-derived cell cultures (HGCC biobank) used in this study. Sheet 3, supplementary video annotations. Sheet 4, comparison chart of invasion assays and positioning of GlioTrace.

File name: Supplementary Movie 1.
Description: (A) Visualization of glioblastoma tumor-bearing slices (tumor cells, green) in 3D culture showing vessel staining (red) through bioavailability of lectin 594 supplemented in the culture medium by capillary action through the transmembrane. The left column shows a region of interest containing tumor cells, and the right column shows a normal brain region from the same brain slice. 18 frames. Video rate: 4 frames per second (fps). Imaging interval: 85 minutes over 24 hours. (B) U3013MG PDCX brain slice model (proneural subtype) showing perivascular migration of glioblastoma tumor cells at the invasive edge within the cortical region. Video generated from 79 frames at 12 fps, highlighting the dynamic interaction between tumor cells and the vascular microenvironment during tumor progression. (C) U3028MG PDCX brain slice model (proneural subtype) showing perivascular migration of glioblastoma tumor cells at the tumor-progressing edge near the subventricular zone in the striatum. 69 frames, 12 fps. (D) U3220MG PDCX brain slice model (mesenchymal subtype) showing perivascular migration of glioblastoma tumor cells at the tumor-progressing edge near the subventricular zone in the striatum. 96 frames, 12 fps. (E) U3054MG PDCX brain slice model (mesenchymal subtype), derived from recurrent glioblastoma, showing perivascular migration of glioblastoma tumor cells at the progressing edge of the tumor in cortex. 69 frames, 12 fps. (F) U3180MG PDCX brain slice model (classical subtype) showing perivascular migration of glioblastoma tumor cells in the striatum. 95 frames, 12 fps. (G) U3179MG PDCX brain slice model (classical subtype) showing discrete migration of glioblastoma tumor cells. 48 frames, 12 fps. Glioblastoma tumor cells are green and vessels are red.

File name: Supplementary Movie 2.
Description: (A) A tumor cell demonstrating branching morphology during glioblastoma invasion in PDCX slice culture. (B) A tumor cell demonstrating perivascular translocation during glioblastoma invasion in PDCX slice culture. (C) Classical subtype glioblastoma showing tumor cells with locomotion, round, and vessel-associated migratory morphologies during invasion in PDCX slice culture. (D) A glioblastoma tumor cell demonstrating a migration-pattern switch from translocation to branching and then back to translocation in PDCX brain slice culture. (E) A glioblastoma tumor cell demonstrating invasion-route change from diffuse locomotion to perivascular translocation. (F) A glioblastoma tumor cell demonstrating vessel-guided migration during brain invasion in slice culture. 92 frames. Videos at 12 fps. Glioblastoma tumor cells are green and vessels are red.

Filename: Supplementary Movie 3.
Description: (A) U3013MG tumor cells disorganizing vessels at the tumor-invasive edge during brain invasion in PDCX brain slices. 69 frames, 2.5 h interval. (B) U3028MG tumor cells disorganizing vessels at the tumor-invasive edge during brain invasion in PDCX brain slices. 69 frames, 0.95 h interval. (C) U3220MG tumor cells disorganizing vessels at the tumor-invasive edge during brain invasion in PDCX brain slices. 96 frames, 1.03 h interval. (D) U3054MG tumor cells disorganizing vessels at the tumor-invasive edge during brain invasion in PDCX brain slices. 71 frames, 1.58 h interval. (E) The vascular network remains unaffected during brain invasion by diffusely growing

U3180MG tumor cells irrespective of vessel contact. 69 frames, 0.95 h interval. (F) Diffusely growing U3179MG cells exhibiting some vessel contacts without overt disruption of vascular integrity. 48 frames, 1.53 h interval. (G) U3013MG tumor cell colocalizing with brain-resident microglia during the interaction period. In this example, the associated microglial cells divide and the tumor cells subsequently undergo cell death. In the video, microglial cells are recognized as dense, motile red cells with irregular or round morphology. 63 frames, 1.42 h interval. Videos at 12 fps. Glioblastoma tumor cells are green; vessels and microglia are red.

Filename: Supplementary Movie 4.
Description: (A) U3013MG slices treated with DMSO, dasatinib, or thapsigargin. Dasatinib and thapsigargin reduce tumor cell migration and brain invasion. 79 frames, 1.5 h interval. Video at 12 fps. (B) U3180MG slices treated with DMSO, dasatinib, or thapsigargin. Dasatinib and thapsigargin reduce tumor cell migration and brain invasion. 87 frames, 1.5 h interval. Video at 12 fps. (C) U3054MG slices treated with DMSO, dasatinib, or thapsigargin. Dasatinib and thapsigargin reduce tumor cell migration and brain invasion. 70 frames, 1.73 h interval. Video at 12 fps. (D) U3220MG slices treated with DMSO, dasatinib, or thapsigargin. Dasatinib and thapsigargin reduce tumor cell migration and brain invasion. 96 frames, 1.03 h interval. Video at 12 fps. (E) U3179MG slices treated with DMSO, dasatinib, or thapsigargin. Dasatinib and thapsigargin reduce tumor cell migration and brain invasion. 74 frames, 1.55 h interval. Videos at 12 fps.

Filename: Supplementary Movie 5.
Description: Representative videos from cortex, corpus callosum, and striatum of U3013MG PDCX slices treated with clodronate for selective depletion of the myeloid compartment, including microglia. 157 frames, 0.77 h interval. 10 fps. Glioblastoma tumor cells are green and vessels are red.

Filename: Supplementary Movie 6.
Description: Comparative video demonstrating integration of tumor cells in mouse brain slices for U3013MG and U3180MG PDCX models compared with tumor cells transplanted onto healthy mouse brain slices. 122 frames, 0.93 h interval. 10 fps.

Filename: Supplementary Movie 7.
Description: Qualitative examples of U3013MG and U3180MG tumor cells migrating along vessels and interacting with CD13-positive pericytes and vascular endothelial cells. Hoechst, nuclei; GFP, tumor cells; lectin 594, vessels; CD13, pericytes. Time-lapse images: U3013MG, 122 frames, 10 fps; U3180MG, 178 frames. Z stacks: U3013MG, 150 z-planes; U3180MG, 180 z-planes; 0.2 µm z-step. Arrows indicate representative individual cells tracked back in the time-lapse imaging that showed physical interaction with CD13-positive pericytes.

Filename: Supplementary Movie 8.
Description: Videos showing tracking and state morphology dynamics and distribution after running GlioTrace on an external dataset from Hansen and Hippenmeyer, 2024, STAR Protocols. Colors denote morphological state at each time point. The left panel shows all channels overlaid; the right panel shows the channel used for cell detection and tracking. The rightmost key displays the real-time distribution of morphologies.
